# Epigenetic machine learning: utilizing DNA methylation patterns to predict spastic cerebral palsy

**DOI:** 10.1186/s12859-018-2224-0

**Published:** 2018-06-21

**Authors:** Erin L. Crowgey, Adam G. Marsh, Karyn G. Robinson, Stephanie K. Yeager, Robert E. Akins

**Affiliations:** 1 Nemours Biomedical Research, Nemours - Alfred I. duPont Hospital for Children, 1600 Rockland Rd, Wilmington, DE 19803 USA; 2 Genome Profiling LLC, 4701 Ogletown Stanton Rd #4300, Newark, DE 19713 USA; 3 Center for Bioinformatics and Computational Biology and The School of Marine Science and Policy, University of Delaware, Newark, DE 19713 USA

**Keywords:** Cerebral palsy, Epigenetic biomarkers, DNA methylation, Genomics, Computational statistics

## Abstract

**Background:**

Spastic cerebral palsy (CP) is a leading cause of physical disability. Most people with spastic CP are born with it, but early diagnosis is challenging, and no current biomarker platform readily identifies affected individuals. The aim of this study was to evaluate epigenetic profiles as biomarkers for spastic CP. A novel analysis pipeline was employed to assess DNA methylation patterns between peripheral blood cells of adolescent subjects (14.9 ± 0.3 years old) with spastic CP and controls at single CpG site resolution.

**Results:**

Significantly hypo- and hyper-methylated CpG sites associated with spastic CP were identified. Nonmetric multidimensional scaling fully discriminated the CP group from the controls. Machine learning based classification modeling indicated a high potential for a diagnostic model, and 252 sets of 40 or fewer CpG sites achieved near-perfect accuracy within our adolescent cohorts. A pilot test on significantly younger subjects (4.0 ± 1.5 years old) identified subjects with 73% accuracy.

**Conclusions:**

Adolescent patients with spastic CP can be distinguished from a non-CP cohort based on DNA methylation patterns in peripheral blood cells. A clinical diagnostic test utilizing a panel of CpG sites may be possible using a simulated classification model. A pilot validation test on patients that were more than 10 years younger than the main adolescent cohorts indicated that distinguishing methylation patterns are present earlier in life. This study is the first to report an epigenetic assay capable of distinguishing a CP cohort.

**Electronic supplementary material:**

The online version of this article (10.1186/s12859-018-2224-0) contains supplementary material, which is available to authorized users.

## Background

Cerebral palsy (CP) is a complex group of conditions that are difficult to diagnose [1–4]. Together, the CPs represent the most common physical disability in childhood, with a prevalence of 1 in 323 children [5]. Spastic CP is the most frequent type accounting for 77% of cases [5]. Spastic CP affects movement and posture with accompanying activity restrictions and disability that impart a very high burden on patients, families, and society. Medicaid data show that annual medical costs are 10–26 times higher for children with CP [6], and problems continue into adulthood with poor access to care, lower employment, absenteeism, disability, and premature death among CP patients having significant impacts [7].

CP arises from a disturbance in the brain, which in most cases occurs prenatally between 24 weeks gestation and birth [2, 8]. Intervention during the early postnatal phases of neuromotor maturation could significantly decrease CP’s profound effects, but despite advances in developmental monitoring, screening, and medical evaluation, the diagnosis of CP remains a considerable challenge [1–4]. In addition, although CP starts in the brain, brain imaging data do not correlate well with CP. Studies indicate that 20–33% of infants who developed CP lacked detectable brain abnormalities on cranial ultrasound evaluation, and MRI and CT imaging data suggest that at least 17% of known CP patients have no detectable brain malformation [9, 10]. Many children exhibit brain imaging indicative of CP even though they do not develop CP [11], and although imaging approaches have shown promise as prognostic tools for children with CP, imaging may not be a reliable indicator of CP [12].

The potential benefits of biomarkers for CP are well-recognized; unfortunately, several promising biomarker studies investigating umbilical cord serum concentrations of S100B, neuron-specific enolase, the total soluble form of the receptor for advanced glycation end-products [13], IL-8, IL-1β, and TNF-α [14], and umbilical cord blood magnesium concentration [15] were unable to distinguish CP from non-CP patients within statistical significance [13–15]. Genetic approaches have also received attention, but genomic studies indicate significant heterogeneity in CP with genetic variants having a burden of 2–14% [16, 17], indicating gene sequence analysis focused solely on variants may be of limited use in the diagnosis of CP.

The onset of spastic CP has been associated with hypoxia, infection, inflammation, and growth restriction [18–20]. Current research indicates that hypoxic exposure, bacterial infection, inflammation, growth restriction, and early life trauma or stress are associated with alterations in DNA methylation patterns [21–28]. Furthermore, sustained epigenetic pattern differences have been identified after prenatal exposure to famine [29], supporting the notion that stress on a developing fetus can alter DNA methylation patterns long-term. Based on these observations, we hypothesized that children and adolescents with spastic CP have specific methylation patterns that distinguish them from a non-CP cohort.

Typical DNA methylation sequencing platforms depend on harsh chemical treatments to mutate unmethylated cytosine bases, such as bisulfite oxidation, which when applied to NGS for analysis leads to computational complexities in aligning mutated sequence reads back to a reference genome. Array based hybridization methods are also commonly used because of their lower cost, but arrays are restrictive in interrogating only a limited number of regions of interest by targeted probes. Here we utilize a non-biased, methyl-sensitive restriction enzyme approach coupled with rigorous statistical modeling for reconstructing cytosine methylation status from NGS data files to identify unique genome-wide DNA methylation patterns associated with a diagnosis of spastic CP.

## Method

### Subject enrollment and sample processing

Twenty-two subjects with a diagnosis of spastic CP and 21 control subjects were enrolled in an IRB-approved study at the Nemours - Alfred I. duPont Hospital for Children after informed consent/assent. Samples were collected and banked from 2014 to 2016 by the Neuro-Orthopedic Tissue Repository at Nemours. The control cohort comprised children with an idiopathic condition, a diagnosis that was unrelated to CP, or an injury. Subjects with a chromosomal disorder, degenerative neurological disease, or muscular dystrophy were excluded.

Blood collected in K_2_EDTA-containing Vacutainer tubes (BD, Franklin Lakes, NJ) was centrifuged at 1300×g for 10 min and the entire white blood cell layer was collected and stored at − 80 °C. Total white blood cell populations without further cell purification were used for DNA isolation. Genomic DNA was isolated from the collected cells using Gentra Puregene Kits (Qiagen, Valencia, CA). DNA quantity and purity were assessed using a NanoDrop ND-1000 spectrophotometer (NanoDrop Technologies, Wilmington, DE) and an Agilent 4200 Tapestation (Santa Clara, CA). All samples had a DNA Integrity Number ≥ 8.5 indicating substantially intact DNA.

### Library preparation and DNA methylation next generation sequencing

DNA libraries were prepared from methyl-sensitive restriction endonuclease (MSRE) [30, 31] fragmented genomic DNA (gDNA) using HpaII, which recognizes C (CpG) G sites. A standard sequencing protocol was then performed including randomized shearing (Covaris, Woburn, MA) and synthesis of a gDNA fragment library using Illumina TruSeq Nano library synthesis kits (San Diego, CA). Next generation sequencing (NGS) was performed on an Illumina ×10 platform by Macrogen USA (Rockville, MD). The protocol generated single end reads (150 bp) with >20× coverage of the regions captured. FASTQ data files were processed to calculate the probability of methylation at individual CpG sites through a commercial bioinformatics pipeline and software platform (Genome Profiling, Newark, DE). For convenience, the term “CpG” in this paper refers to “C (CpG) G” HpaII restriction sites. Validation of the HpaII approach was carried out using “spike in” DNA sequences synthesized with known methyl-CpG composition (see Additional file 1: Figure S1).

### Statistical analysis

Analysis focused on methylation at individual CpG sites. Metrics were derived from the sequencing output files (FASTQ) and utilized to derive a score proportional to the probability that a specific CpG site was methylated within the mixed population of peripheral blood cells collected for each subject. Differential methylation patterns between the groups were identified using a non-metric multidimensional scaling (NMDS) ordination analysis [32]. Comparisons across samples were used in a retrospective analysis to identify patterns that were highly conserved within each group while also diverging between groups. The open-source statistical environment “R” (https://www.r-project.org/) was used for analysis including the module *vegan*, which contains robust ordination routines.

To identify the CpG sites contributing most significantly to differences in methylation, we employed algorithms designed for differential gene expression using a hybrid of modules from the R-packages, *edgeR* and *limma* [33, 34] to execute pairwise comparisons (control vs. CP) for each CpG site. The smaller response scale of the methylation data is well within the operational boundaries of the data distributions (log-scale) of gene expression data sets and the well-developed false-discovery rate calculations in these expression packages are quite robust for the normally distributed linear data of methylation scores. Methylation differences were compared for single CpG sites using tagwise dispersion for site-specific false discovery rate correction applied to each pairwise comparison. Statistical significance was evaluated using a Likelihood Ratio Test with a one-way ANOVA-like contrast (LRT-ANOVA).

To evaluate methylation profile shifts across higher genome structure scales, differential methylation load (∆ML) was first calculated as CpG site specific differences between groups. These methylation differences were summed across 1 Mbp intervals and normalized by the total number of CpG sites present. Positive ∆ML values indicated more methylation in the control group; negative values indicate more methylation in the CP group.

### Gene annotations

Gene annotations were derived from the ENSEMBL database with UniProt gene identifiers with their defined promoters, 5’ UTRs, exons, introns, and CpG Islands (accessed through the UCSC Genome Browser at https://genome.ucsc.edu/index.html). Annotated sites were assigned to functional groups and pathways using hierarchical levels defined in KEGG biological pathway and GO gene ontology classification schemes. Here we report primary results using the UniProtKB gene identifiers with ∆ML values calculated across the fully defined gene body domain (inclusive of 2 kb upstream promoter domains). Gene-level bioinformatics analysis of the datasets were carried out using the system biology tools Cytoscape v3.3.0 [35] and the reactomeFI plugin (database 2015) [36].

## Results

Methylation patterns were analyzed in 32 genomic DNA samples from peripheral blood cells: 16 subjects with a diagnosis of spastic CP (13 males, 3 females; age = 14.7 ± 3.3) and 16 controls (15 males, 1 female; age = 15.0 ± 2.2). There were no significant differences in differential counts for nucleated blood cells between the CP and control cohorts (p >> 0.05). Whole genome methylation patterns were acquired by NGS after methylation sensitive restriction endonuclease (HpaII) digestion. The hg19 reference genome assembly from the University of California Santa Cruz (UCSC) includes 2.29 M HpaII target C (CpG) G motifs, which represent ~ 15% of the 14 M CpG sites in the haploid hg19 genome. When the HpaII restricted sites in our 32 samples were aligned, 1,468,477 sites were in common across all subjects.

**Fig. 1:**
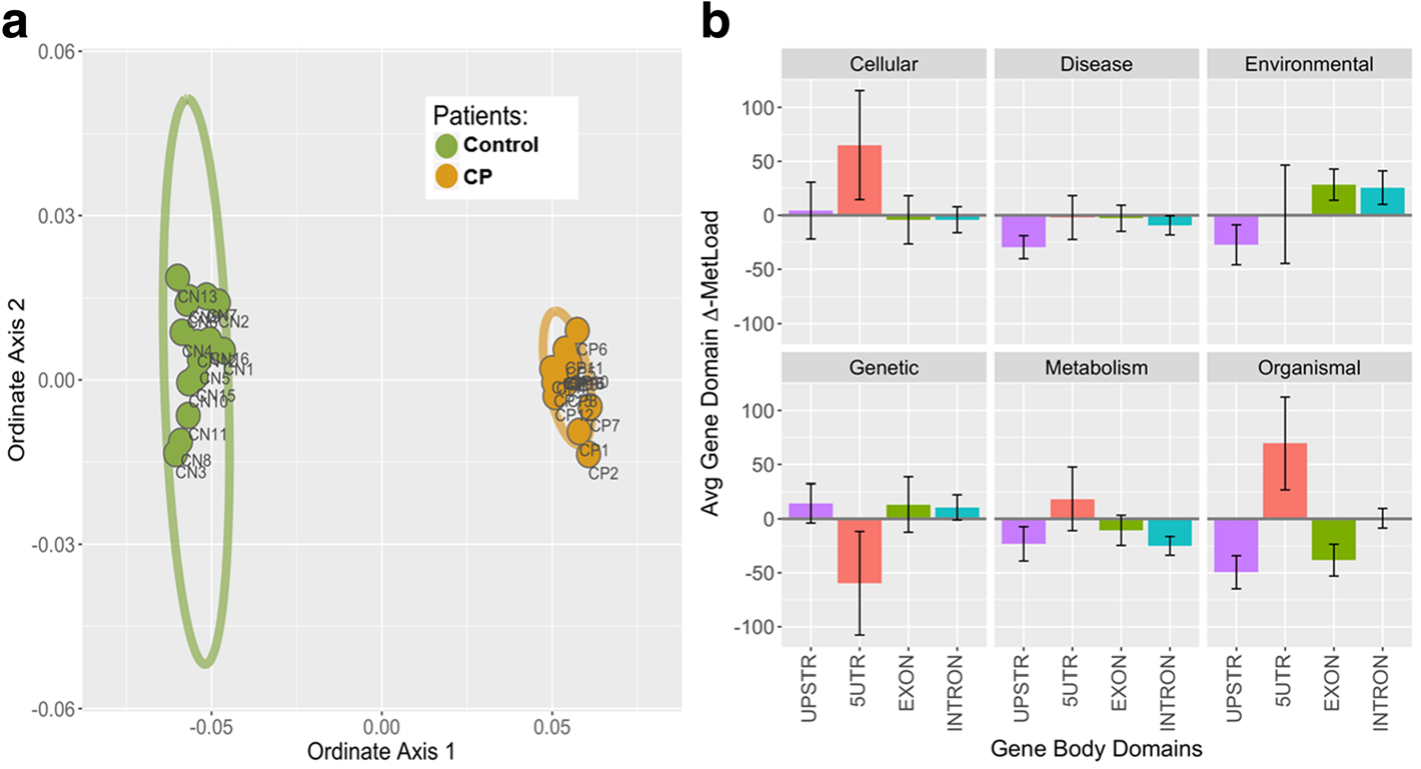
Statistical Methylation Patterns. **a** Non-Metric multidimensional scaling to identify discriminating cytosine methylation patterns between CP and non-CP cohorts. The first two component axes were plotted to locate the individual subject points in a relative 2D plane. Each point represents the similarity position of a subject based on all potentially informative CpG sites (*n* = 61,278). CP = orange points; controls = green points. Ellipses represent 90% confidence intervals. The complete segregation of the two cohorts indicates that DNA methylation patterns fundamentally differ between the cohorts. **b** Comparison of differential methylation load by KEGG functional classification and domain structure. ∆ML (mean difference between CP and control groups) was calculated across the defined length of the gene body structure for six top-level KEGG Pathway Map Classifications: Cellular Processes, Human Diseases, Environmental Information Processing, Genetic Information Processing, Metabolism, and Organismal Systems. Positive ∆ML numbers indicate higher methylation in control subjects and negative numbers indicate higher methylation in CP subjects. Assessing ∆ML score demonstrated a prevalence of altered methylation in 5’ UTR regions for three of the hierarchical KEGG functional categories. Values plotted are means +/− SEM across the number of genes scored in each category

To assess if discrimination between the CP and non-CP groups was possible, an ordinate analysis technique of non-metric multidimensional scaling (NMDS) was performed. NMDS is an iterative, rank-based approach that collapses complex, multi-dimensional datasets into a small number of components that represent the differential relationships within the original data. NMDS allows visualization of patterns that are conserved within groups but that diverge between groups. All potentially informative CpG sites (*n* = 61,278 out of the 1,468,477), which were defined a priori as having between group methylation score differences of at least 10%, were integrated as one pattern. The two primary ordinate axes showed a strong discrimination between the CP and non-CP subjects (Fig. 1a).

To determine whether the methylation pattern differences between the groups were associated with specific genetic regions, ΔML scores, which were calculated as the mean difference between CP and control groups, were mapped to high-level KEGG functional classifications and gene structure categories (Fig. 1b). There was a striking prevalence of altered methylation in 5′ untranslated regions (UTR) and in upstream promoter domains.

**Fig. 2:**
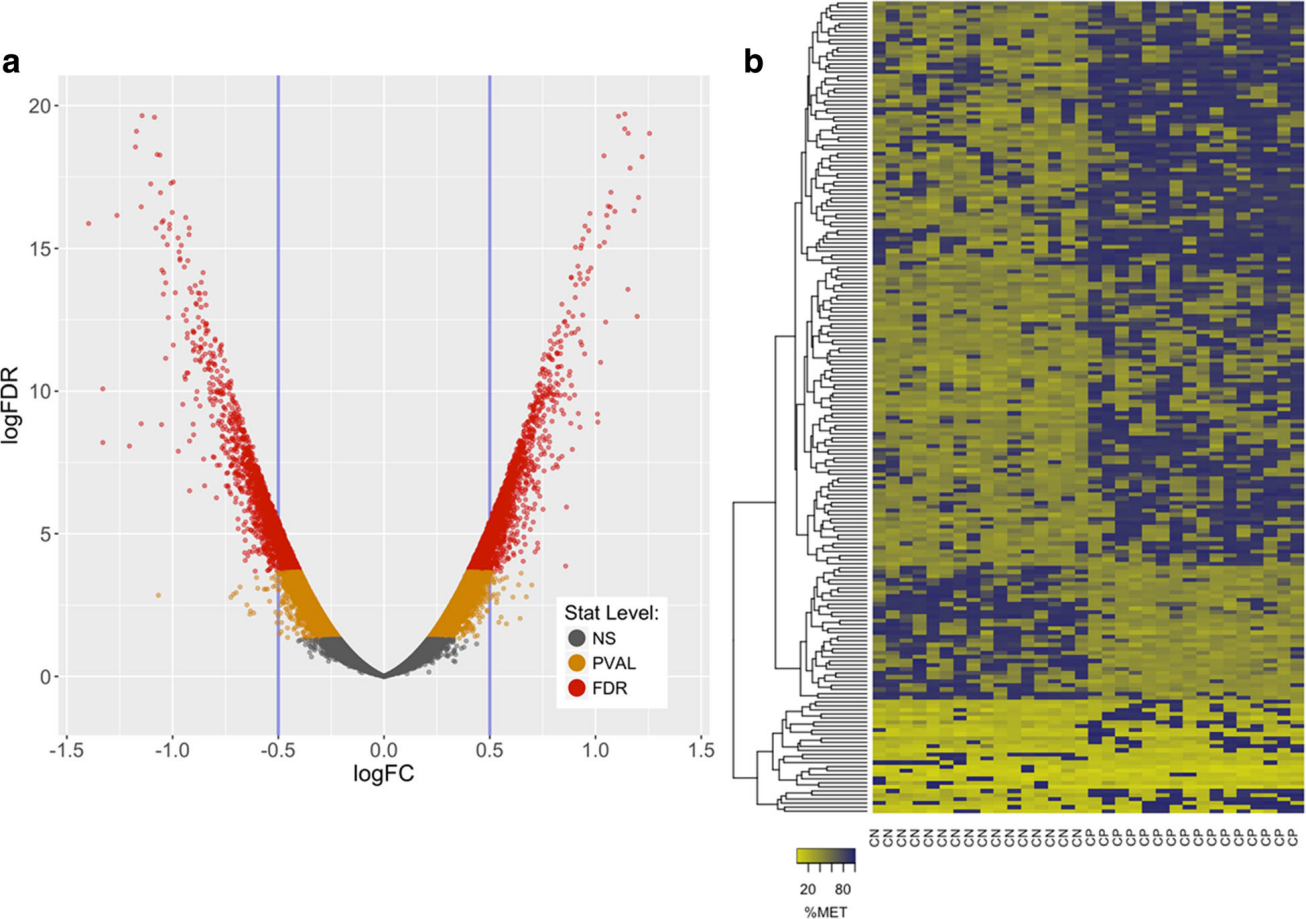
CpG Statistical Comparisons Based on a Likelihood-Ratio-Test of a One Way ANOVA contrast. **a** Volcano plot with the frequency profile of *p*-values. Data from the 1.47 million CpG sites in common across all samples are plotted. The x-axis is the log value for the fold-change (ratio) of CP to non-CP CpG site methylation values and the y-axis is the log FDR value (gray = not significant, orange = *p*-value significant, and red = *p*-value after FDR significant). **b** Heatmap Clustering of the top 200 CpG sites selected based on statistical *p*-value. There were 6588 CpG sites that were significantly different (*p* < 0.05 after false-discovery rate correction). Hierarchical clustering based on % methylationwas employed using the 200 CpG sites with the lowest *p*-values. Quantitative differences in CpG site methylation by diagnosis were apparent. Each row represents the score for a single CpG site across all subjects

A volcano plot (Fig. 2a) shows the distribution of the 1.47 M CpG sites plotted as the log_2_ of the fold-difference between CP and control versus the negative log of the false discovery rate (FDR) corrected *p*-values for each CpG site comparison (− 1*log_2_ [FDR]). Pairwise comparison of individual CpG sites using a Likelihood-Ratio Test (LRT-ANOVA) revealed 6588 differentially methylated CpG sites with significant *p*-values after FDR correction (p [FDR] < 0.05); 2809 hypo-methylated and 3779 hyper-methylated sites in the CP cohort. The 200 top-ranked CpG sites (Additional file 1: Table S1) were subjected to hierarchical clustering and visualized using a heat map plot based on the % methylation for each site (Fig. 2b), which revealed sets of CpG sites with concordant methylation patterns. These results support the notion that differences in DNA methylation patterns between the two cohorts had distinguishable levels of organization (see also Additional file 1: Figure S2). Of the 6588 differentially methylated CpG sites, 2903 were located within annotated gene bodies and 3685 were un-annotated (relative to the UCSC Genome Browser defined gene bed file for hg19).

**Fig. 3:**
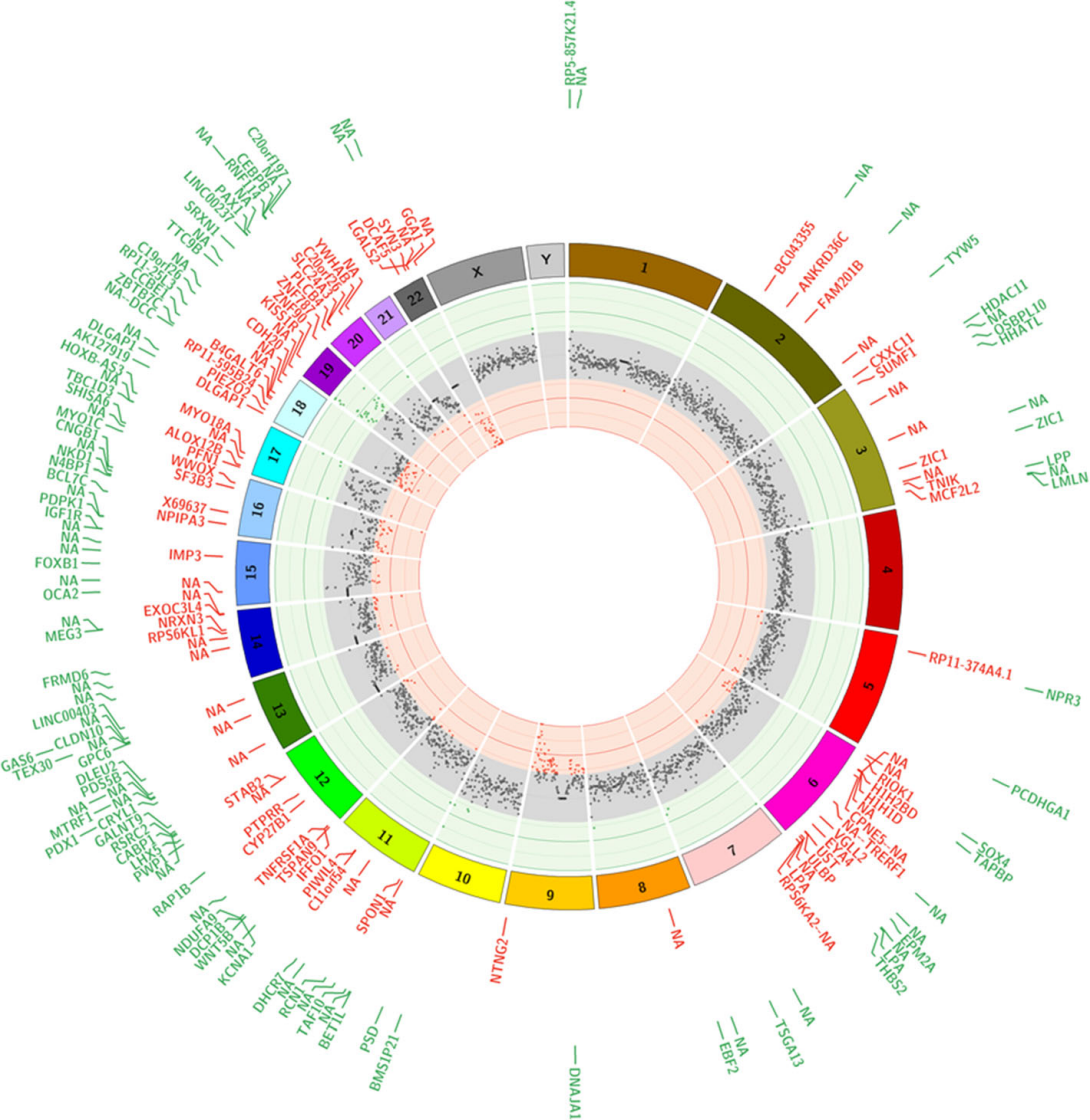
CpG Methylation Load Ideogram Comparing CP and Control Cohorts. Mean differences in CpG methylation scores (∆ML; control minus CP) were used to calculate a summation methylation load score at 1 Mbp intervals. ∆ML is presented as the inside track using a scatter plot to show higher methylation in controls (green), higher methylation in CP (red), and equivalent methylation in both (gray; abs|∆ML| is less than twice the average ∆ML for the whole genome). The particular “hotspots” that appear in chromosomes 9, 18, 19, and 22 could indicate allelic compositional differences and potential gene targets for future functional and validation studies. The chromosomal locations of the top 200 CpG sites are indicated in two rings by tick marks labeled with the gene name (or “NA” if there is no annotation) in which the CpG site is located. Those CpG sites that had significantly higher methylation levels in the controls are in green. Those CpG sites that had significantly higher methylation levels in the CP subjects are in red. The distribution of sites appears skewed toward chromosomes 11 to 22

For additional visualization of high-level, genome-wide methylation, Fig. 3 shows differential methylation loads (ΔML) across 1 Mbp segments mapped to chromosomal locations for the top 200 individual CpG sites. Accentuated ΔML differences were noted in chromosomes 9, 18, 19, and 22. In addition, at the level of individual CpG sites, there was a distinct and apparently non-random distribution of the top 200 sites concentrated in chromosomes 11 to 22.

Genes with demonstrable differences in methylation load scores are presented in Additional file 1: Table S2. Because the primary methylation signal being quantified pertains to circulating peripheral blood cells, there is a prominent functional signal in the number of cytokine signaling pathway components, which are categorized as ‘olfactory signal pathways’ for genes originally identified in small-molecule sensory transduction pathways. The majority of gene level methylation changes can be traced to cell-surface signal reception and transduction genetic components. Methylation loads at higher levels of gene organization were assessed using methylation densities, and pathway enrichment analysis of the top 100 hypo and hyper differentially-methylated genes identified six significantly (*p*-value < 0.05; FDR < 0.01) impacted cellular signaling pathways: G-protein-coupled receptors (GPCR) downstream signaling, olfactory transduction, PI3K-Akt signaling pathway, signaling by NOTCH1, signal recognition particle-dependent co-translational protein targeting to membrane, and protein export.

Given the strong separation signal evident in Fig. 1**,** and the statistically significant differences seen in CpG site methylation at multiple scales, we evaluated the potential predictive value of differential CpG methylation as an epigenetic biomarker of spastic CP. An iterative, bootstrap approach was constructed to sequentially evaluate CpG combinations using linear discriminant analysis (LDA) under the guidance of a machine-learning algorithm. We developed an ML strategy that is similar to the way a random forest walk would explore a response space, but with the included dimension of an ensemble model approach to integrate a multitude of predictive equations. Our goal was to randomly select CpG sites for use in training and test set samples from the main cohort (*n* = 32, 16 CP and 16 non-CP) and to then execute a series of directed prediction models that would be repeated sufficiently to saturate the possible model response space.

For each model, a random set of between 15 and 40 CpG sites was selected from the top 200 CpG sites as ranked by statistical significance in the LRT-ANOVA pairwise tests (see Fig. 2). The 200th rank cutoff was identified as a depth to which the most likely ‘best-predictors’ would be found and to which the computational complexity could be executed in a reasonable amount of time (< 24 h) via distributed processing on a 36-core server. CpG sites deeper in the list may still be relevant for understanding the mechanism of action of the immune system’s response to disease stress, but the predictive power of sites lower than the 200 cutoff would be unlikely to improve the diagnostic power we have found just using the top 200 sites. Eight controls and 8 CP subjects were randomly chosen from the 32 adolescent subjects to use as a training set for an LDA determination. The remaining samples were used as the validation set for scoring.

To factor out subject bias in the training and test set selection, the LDA for each model was repeated 20 times with different random subject selections. If the aggregate sensitivity and specificity across all 20 replicates was > 98% and > 90%, respectively, that model set of CpG sites was scored as a “good” model. At intervals (approx. every 10,000 model evaluations), the performance of CpG sites in the “good” models were ranked to increase the selection of those sites in future tests. In this way, potentially predictive models were developed from the cohort of 32 adolescents. Overall, 6.8 million LDA runs were evaluated on different CpG model sets. Despite the potential confounding variables, we are confident that the statistical approach primarily discriminated CP vs non-CP differences because the accuracy of identifying CP from non-CP subjects was not correlated with sex or any other demographic variables available to us.

The approach yielded 252 sets of CpG sites that acted as high-performing models with near-perfect internal accuracy among the adolescent subjects in our original cohort. These machine-learning results strongly indicated that diagnostic tests for spastic CP were possible using a relatively small set (< 40) of CpG sites. To test the predictive value of these CpG biomarker sets in clinically-relevant samples, the LDAs for the 252 best performing models were employed to “vote” on the identity of blinded samples from significantly younger subjects closer to the age of typical diagnosis. 11 subjects, 6 CP patients (5 males, 1 female; age = 3.7 ± 1.5) and 5 control patients (4 males, 1 female; age = 4.2 ± 1.4), were evaluated.

**Fig. 4:**
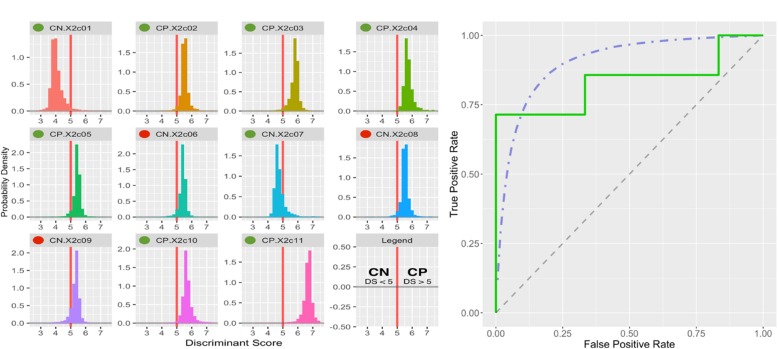
Receiver Operator Curves for Model Classification Test. A bootstrap classification model was executed using a linear discriminant analysis (LDA) guided by a machine-learning algorithm. **a** A test group comprising young children (approx. 4 years old; *n* = 11) was used with discriminant scores from each LDA normalized to a center point of 5. The majority of model “votes” either < 5 or > 5 was used to classify each sample. Green and red marks indicate correct and incorrect identifications, respectively. **b** Receiver operator characteristic (ROC) curves for an iterative theoretical yield (blue dashed line) and the actual yield from the classification tests of the 1–5 yo group (green line). Here, overall accuracy was 73% with a sensitivity of 100%, specificity of 40%, and an area under the curve (AUC) of 0.691. The performance of this dynamic classification analysis suggests that there is high discrimination power that could be developed for diagnostic detection of spastic CP

A total of 5040 classification scores (20 LDA determinations for each of 252 “good” CpG sets) were determined for the blinded samples. The probability distributions of the polling LDA scores are shown in Fig. 4a. In Fig. 4b, receiver operating characteristic (ROC) curves are plotted from the actual true-positive and false-positive rates observed in the patient classification results of the younger cohort. A maximum theoretical curve generated from saturated bootstrap sampling of the older cohorts is also shown.

## Discussion

Currently, the diagnosis of CP is based on the disruption of normal development and movement and is therefore limited in the ability to provide opportunities for early intervention. A biomolecular screening assay that can measure biomarkers in the blood, preferably collected at the time of birth rather than the time of typical diagnosis, could allow for earlier diagnosis and intervention. A major challenge with identifying such systemic markers is the need to develop data-driven approaches that can function without a priori knowledge. High throughput genomic analyses provide opportunities for such approaches, but studies of CP cohorts have indicated significant heterogeneity between subjects, and have reported that genetic variants have a burden of 2–14% depending on the study [16, 17]. Ultimately, gene sequence heterogeneity has limited the effective use of genomic mutations as an early diagnostic of CP. To date, DNA methylation assays have not been broadly applied in the study of CP biomarkers; however, recent studies suggest that differences in the prevalence of CP in monozygotic twins may be associated with alterations in DNA methylation [37, 38], and numerous studies have demonstrated that various stress stimuli, such as hypoxia, infection, and inflammation, cause a long-lasting change to DNA methylation patterns [22]. Utilizing a novel epigenetic biomarker assay, this is the first report demonstrating a retrospective model capable of identifying CP samples based on blood DNA methylation patterns.

Our approach utilized two primary analytic paths: ordinate based NMDS analysis of methylation patterns in total, and site-paired, LRT-ANOVA-based contrasts for individual CpG locations. In a retrospective, pattern-level analysis, NMDS (Fig. 1 Panel a) revealed a strong discrimination between the two cohorts, indicating that DNA methylation patterns were fundamentally different between the spastic CP and non-CP groups. The LRT-ANOVA analysis complemented those results by identifying site-specific CpG % methylation changes that drove the pattern differentiation between the groups (Fig. 2). Plotting of the top 200 CpG sites (Fig. 2 Panel b), revealed a distinct pattern between the cohorts analyzed and demonstrated consistent quantitative differences in CpG site methylation among related samples. The volcano plot (Fig. 2 Panel a) shows the full distribution of observations in the data set with all comparisons for the full set of 1.47 M CpG sites. The statistically-significant CpG sites after false discovery rate correction (*n* = 62,357; shown in red) include the top 200 sites ranked by *p*-value that appear in the methylation heatmap (Fig. 2 Panel b). These combined results indicate that CpG methylation differences can be resolved to individual CpG sites and that these may be key in understanding how methylation patterns in CP subjects may be associated with the presence of a disease or stress state not present in control subjects. Understanding the cellular and biological antecedents of these epigenetic changes could hold the key to utilizing epigenetic biomarkers in early disease diagnoses.

Assessing ∆ML score (mean difference between CP and control groups) demonstrated a prevalence of altered methylation in 5’ UTR regions for three of the hierarchical KEGG functional categories (Fig. 1 Panel b). Of interest, genetic information processing was one of the three functional categories, suggesting that aberrant DNA methylation patterns detected in the CP cohort are disrupting fundamental processes, including transcription, translation, and DNA replication and repair. The chromosomal localization of the top 200 CpG site differences (Fig. 3) shows a skewed distribution of differentially methylated sites concentrated across chromosomes 11 to 22. The particular “hotspots” that appear in 9, 18, 19, and 22 could indicate allelic compositional differences and reveal potential genes to target for future functional and validation studies. The approach of putting CpG methylation events into both gene and chromosomal contexts will likely help focus efforts to understand why some epigenetic sites may be effective biomarkers of spastic CP based on specific mechanisms of action linked to disease onset, progression, or response to disease stressors.

A simulated classification analysis, utilizing an iterative boot-strap classification approach, strongly demonstrated the power of methylation analysis with high sensitivity and specificity (Fig. 4). Despite the small sample size and > 10 year difference in subject ages between the training sets and the validation set, the positive predictive value (PPV) for the CpG-methylation biomarker test was 67%, which outperformed the PPVs reported in studies linking CP to movement assessment (24%), brain imaging (32%), or combined movement plus imaging measures (54%) [39]. In addition, a distinct classification signal persisted with an overall accuracy of 73% and a sensitivity of 100%. The sensitivity and overall accuracy of the test were surprisingly strong given that the LDA-based assessments were based entirely on much older subjects despite potential effects of age on DNA methylation patterns [40]. Thus, although the validation testing on the younger cohort was not perfect and had low specificity (40%) and an area under the ROC curve of only 0.691, further refinement of the DNA methylation pattern approach to account for age differences is expected to improve the specificity and the diagnostic capability of the test.

Linking DNA methylation patterns in blood cells to specific health risks or diseases is an area of active research. Recent studies describe blood cell epigenetic markers for: pediatric cardiac risk [41], immune stress associated with sclerosis [42], infant cerebral palsy markers in cord blood [38], regulatory T cell imprinting as a marker for therapeutic efficacy [43], and immuno-profiling for specific disease / stress states [44]. These types of studies demonstrate that information about past environmental exposures and health/disease status are present in blood cell methylation patterns and suggest that such alterations are present in the hematopoietic stem cell populations that give rise to circulating myeloid and lymphoid cells.

In the long-term, we seek to determine whether robust and stable DNA methylation patterns exist in peripheral blood that identify individuals with spastic CP early in life. Such patterns in unfractionated blood cells would allow for development of straightforward diagnostic approaches that do not involve complex cell fractionation. Although not yet definitive, the current results strongly support the possibility that a DNA methylation signal for spastic CP exists. The methylation patterns detected in the peripheral blood samples used in the study likely arose from a combination of early-life stress and the accumulation of other changes affecting hematopoietic stem cell populations over time. In particular, blood stem and progenitor cells depend on methylation for function but can also carry methylation tags as a type of “epigenetic memory” [45]. We hypothesize that the DNA methylation differences detected in the CP cohort compared to the control cohort, resulted from inflammatory events associated with the onset of CP. Thus, we suspect that the DNA-methylation fingerprint found in the spastic CP cohort studied here was present earlier in life and may represent a sustained biomarker for spastic CP. This idea requires further investigation.

A limitation of the current study is our reliance of blood samples from older children and adolescents. These samples were chosen for several reasons: the availability of age matched controls presenting to the hospital for surgery, the reliability of diagnosis in the cohort, and the limited access to early-in-life blood samples. One of our future goals is to screen subjects at time of birth using our developed method and mathematical modeling. Furthermore, although this study was focused on spastic CP, analysis of a broader range of CP phenotypes as well as samples from subjects with other neuromotor and developmental conditions is needed to assess the utility of the technical approach in broad clinical settings. Overall, however, the described approach provides the first blood-based test to distinguish reliably a cohort of subjects with spastic CP.

## Conclusions

Overall, our early results strongly indicate that potentially-diagnostic DNA methylation pattern differences may exist for spastic CP and that these differences may persist across patient ages. A biomolecular screening assay based on blood biomarkers, preferably collected at the time of birth rather than the time of typical diagnosis, could allow for earlier diagnosis and intervention than is currently possible. Although a full diagnostic test will require significant development, our data indicate that such a test may be possible based on the analysis of peripheral blood cells utilizing advanced epigenetic analyses.

Importantly, our genome-wide methylation analysis of adolescent samples indicated that diagnostic models with high specificity and sensitivity are possible and provided models that were able to distinguish young children with spastic CP from controls. Implementation of a clinical assay capable of distinguishing a subject at high risk for CP at time of birth will enable earlier intervention, which will hopefully impact disease progression. Furthermore, the novel method described here for data generation and analysis can be broadly applied to other diseases. Implementing machine learning approaches for high-scale genomics data is becoming essential, and will drive our ability to robustly classify subjects into disease and risk-stratification groups.

## Additional files

Additional file 1: **Figure S1.** %Methylation Scoring. Distribution of calculated %Methylation scores for known spike-in standards. Boxplots show the distribution of scores for each methylation standard assessed. **Figure S2.** Hierarchical Clustering of Top Discriminating CpG Sites. Clustering of the CpG methylation profiles for individual CpG sites that are correlated together. Red boxes indicate cluster branches that were present in 100% of the iterative boostrap models executed. **Table S1.** The top 200 CpG sites ranked by statistical significance in % methylation. **Table S2.** Top 200 Differentially Methylated Genes. (PDF 1730 kb).

## Abbreviations

AUC: Area under the curve
CP: Cerebral palsy
FDR: False discovery rate
gDNA: Genomic DNA
LDA: Linear discriminant analysis
LRT-ANOVA: One-way ANOVA-like contrast
MSRE: Methyl-sensitive restriction endonuclease
NGS: Next generation sequencing
NMDS: Non-metric multidimensional scaling
PPV: Positive predictive value
ROC: Receiver operating characteristic
UCSC: University of California Santa Cruz
UTR: Untranslated regions
ΔML: Differential methylation loads
