# Supplementary material for: Epigenetic machine learning: utilizing DNA methylation patterns to predict spastic cerebral palsy
Source: BMC Bioinformatics. 2018 Jun 21;19:225. doi: 10.1186/s12859-018-2224-0 (PMC6011336; doi:10.1186/s12859-018-2224-0)
Supplement: Supplementary file 1 — Figure S1. %Methylation Scoring. Distribution of calculated %Methylation scores for known spike-in standards. Boxplots show the distribution of scores for each methylation standard assessed. Figure S2. Hierarchical Clustering of Top Discriminating CpG Sites. Clustering of the CpG methylation profiles for individual CpG sites that are correlated together. Red boxes indicate cluster branches that were present in 100% of the iterative boostrap models executed. Table S1. The top 200 CpG sites ranked by statistical significance in % methylation. Table S2. Top 200 Differentially Methylated Genes. (PDF 1730 kb). [file 12859_2018_2224_MOESM1_ESM.pdf]

SUPPLEMENTAL INFORMATION

Epigenetic Machine Learning: Utilizing DNA Methylation Patterns to Predict Spastic Cerebral Palsy

Authors: Erin L. Crowgey, Ph.D., Adam G. Marsh, Ph.D., Karyn G. Robinson, M.S., Stephanie K. Yeager, M.S., and Robert E. Akins, Ph.D.

Introduction

Supplemental information is provided in four key areas: validation of the CpG methylation scoring approach (Figure S1); clustering of the top discriminating CpG sites (Figure S2); a list of the top differentially-methylated individual CpG sites (Table S1); a list of the top differentially-methylated genes (Table S2). Please note that Tables S1 and S2 are formatted to be larger than standard printer paper.

There are challenges to using methyl-sensitive restriction endonuclease (MSRE) approaches when potential digest cut sites are located in close proximity to one another (i.e., within the length of a sequence read). To address this concern, 10 kb amplicons from the genome of a model plant system, *Arabidopsis thaliana*, were used to construct %methylation standards for use as “spike-in” controls to validate the CpG methylation scoring in the MSRE approach. Each amplicon contained between 40-45 CCGG HpaII restriction sites. For each amplicon, 100% methylated controls were commercially synthesized (Austrian Institute of Technology, Vienna, Austria), and mixtures were made for 20%, 40% and 60% methylation standards. These spike-in amplicons were then added to gDNA samples from four human reference genomes (HG001-HG004, Coriel Institute Biorepository, USA), and the combined

samples were processed by MSRE and Next-Generation Sequencing with post sequencing analysis of the FASTQ files as in the main text. The plant reference sequences were treated as extrachromosomal elements during alignment. In **Figure S1**, the distribution of %methylation score values are plotted against the known control spike-in %methylation standard values. After applying a filter for coverage (> 30X) and structural complexity, the overall percent error of the 234 CpG values scored (3 amplicons x 4 NGS samples) was +/- 13.4% (median). The correlation between Observed vs. Expected %methylation was 0.7792 and highly significant ($p < 2E-16$). Scoring performance was highest on the 60% standard.

To look for potential associations between specific CpG sites, the top ranked sites with the highest contributions to spatial group separation in the NMDS ordinate analysis were isolated and compared for similarity in methylation patterns across all individuals. Hierarchical clustering with iterative boot-strap analysis (1,000 repetitions) using the R package *pv-clust* identified subgroup relationships and established confidence levels of support for each associative branch. Clusters surrounded by a red box indicate groups of CpG sites with >97% support. These clusters are shown in **Figure S2.**

Two tables are presented to provide both the top CpG sites and the genes with the most significant methylation differences. In **Table S1**, the top 200 CpG sites are ranked by statistical significance in % methylation between cohorts. Columns provide information on UniProt/Hugo gene annotations (NA = no annotation), the chromosomal position of each CpG site referencing the cytosine position on the + strand (hg19), the $\log_2$ of the fold change between group means (ratio CP:non-CP), the p-value from the pairwise Likelihood-Ratio Test, the false discovery rate corrected p-value, and a directional Up or Down methylation change indicator where -1 (green)

indicates CP < CN and +1 (red) indicates CP > CN. In **Table S2**, the top differentially-methylated genes in control vs spastic cerebral palsy subjects are provided. Genes with positive MethyLoad scores had total methylation that was greater in the control subjects; those with negative MethyLoad scores had methylation greater in spastic CP. The top 100 genes that were hypermethylated in the control cohort and the top 100 genes hypermethylated in spastic CP are ranked by differential methylation load scores.

Figure S1. %Methylation Scoring. Distribution of calculated %Methylation scores for known spike-in standards. Boxplots show the distribution of scores for each methylation standard assessed.

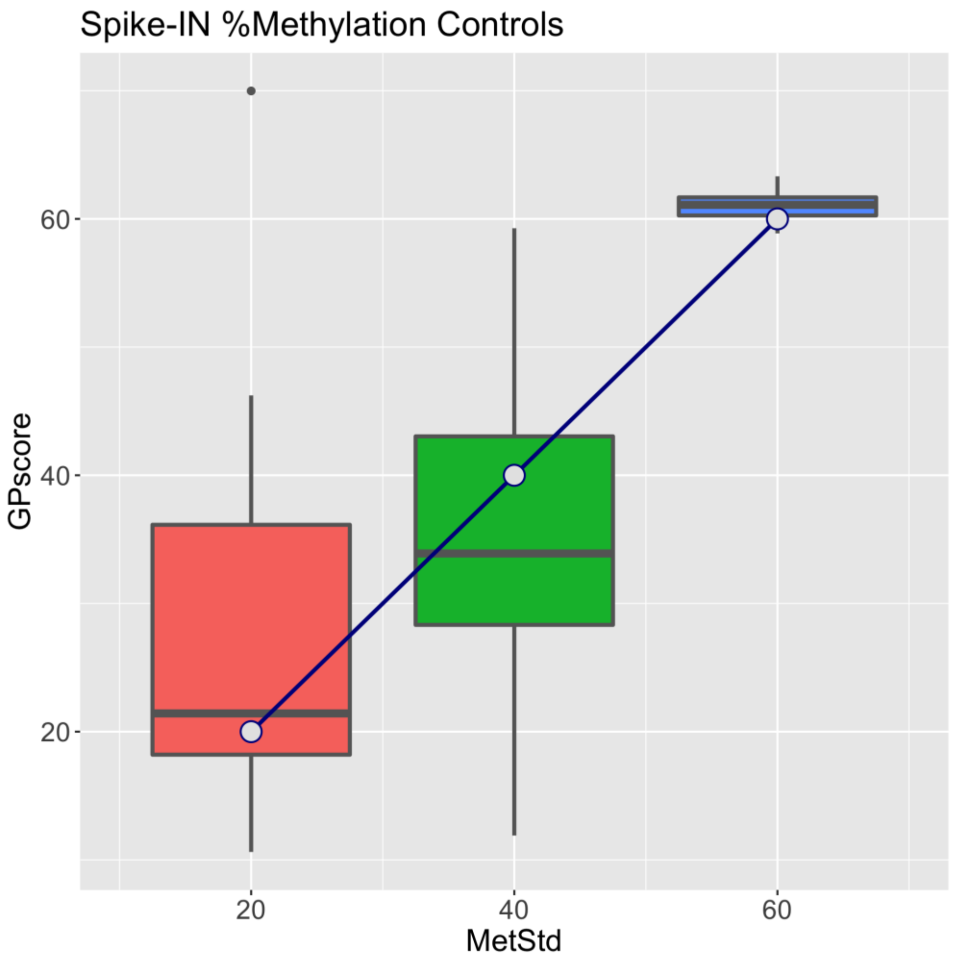

Figure S2. Hierarchical Clustering of Top Discriminating CpG Sites. Clustering of the CpG methylation profiles for individual CpG sites that are correlated together. Red boxes indicate cluster branches that were present in 100% of the iterative boostrap models executed.

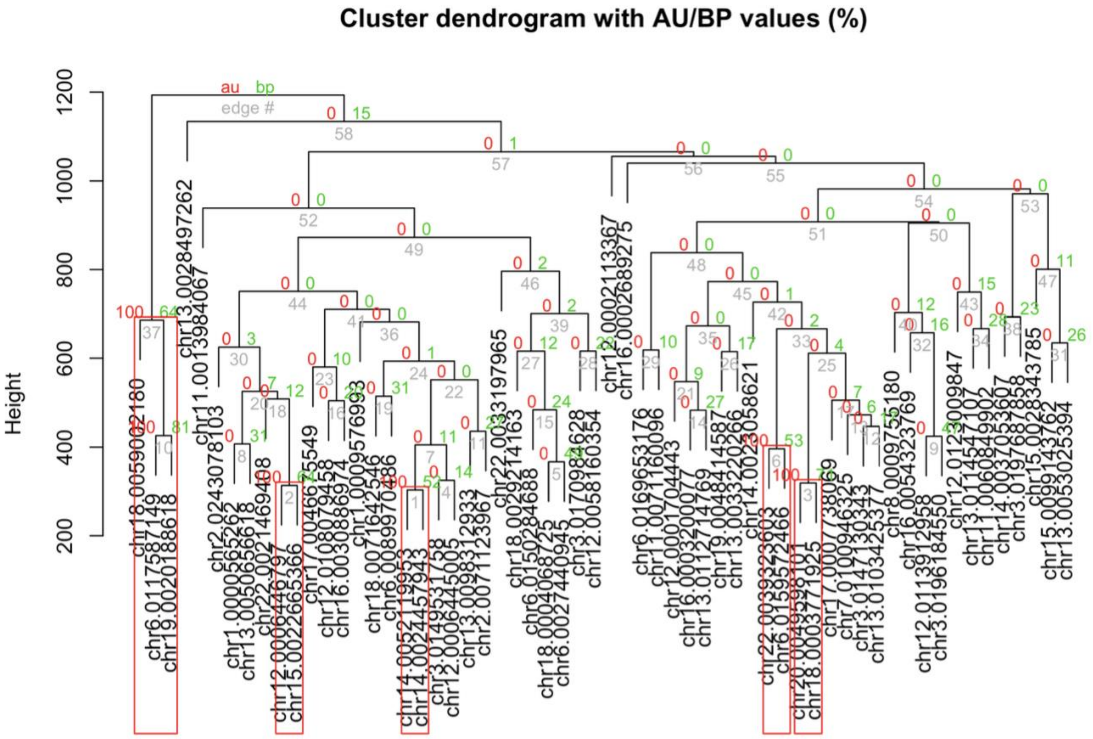

Table S1. The top 200 CpG sites ranked by statistical significance in % methylation.

Gene Annotation	Chromosome Position	Log Fold Change	p-value	p(FDR)	Methylation Up/Down in CP	Control																CP															
						xx039	x053	x056	x057	x370	x380	x414	x416	x425	x431	x436	x466	x489	x527	x563	x584	x010	x028	x045	x050	x375	x376	x417	x440	x444	x533	x568	x573	x583	x597	x600	x606
TNIK	chr3.0170984628	1.722	3.62E-39	5.54E-33	+1	11.44	20.05	21.63	12.33	17.68	16.48	16.00	17.65	15.54	13.77	19.44	14.56	15.57	18.67	21.34	13.91	14.10	92.25	17.44	91.96	11.76	94.44	95.48	12.74	93.11	19.34	17.35	16.78	95.78	95.10	20.34	94.90
LHX5	chr12.0113912958	-1.486	1.65E-31	1.26E-25	-1	97.12	96.70	15.96	96.90	24.51	18.00	16.24	14.64	95.18	96.68	23.49	91.05	92.04	16.22	97.49	20.12	15.69	20.90	21.13	11.62	27.10	19.10	24.83	29.30	19.63	26.83	11.13	18.09	16.10	17.10	19.92	26.26
CDH20	chr18.0059002180	1.221	2.42E-25	7.40E-20	+1	35.29	31.44	42.82	31.37	32.03	27.58	28.79	23.75	35.58	42.61	22.23	41.82	30.66	25.71	30.90	23.90	25.90	35.23	37.34	90.71	92.17	90.54	91.98	96.74	91.14	92.76	91.72	91.36	92.70	36.37	92.13	35.39
NA	chr1.0009576993	-1.521	1.14E-24	2.90E-19	-1	96.00	14.02	12.44	96.89	6.44	95.98	96.40	12.10	11.33	8.39	14.37	10.50	9.74	12.46	10.34	13.61	14.21	9.26	6.91	10.45	8.24	10.08	9.76	15.44	12.82	12.14	8.26	14.88	15.51	16.57	10.51	5.41
NA	chr16.0054323769	-1.212	4.19E-24	8.36E-19	-1	88.98	90.33	9.10	92.41	92.24	13.67	89.31	16.12	92.20	92.03	13.21	90.51	91.84	14.11	91.45	88.82	91.28	10.89	9.84	10.79	13.74	13.59	16.23	15.28	92.08	14.83	11.78	8.86	15.35	15.87	93.87	24.82
DCAF5	chr22.0021469408	2.017	4.37E-24	8.36E-19	+1	0.05	7.24	0.86	5.49	0.97	12.72	15.41	0.60	6.23	8.70	3.31	0.76	0.05	0.05	1.95	0.05	0.05	0.68	13.57	26.63	13.75	98.44	96.77	0.89	9.58	4.09	0.05	0.05	2.31	0.05	0.05	0.05
NA	chr14.0023058621	-1.257	9.76E-24	1.66E-18	-1	90.90	15.54	12.18	24.65	17.85	92.47	95.81	20.76	15.55	18.50	93.67	97.07	91.37	10.72	94.76	94.65	48.97	17.82	9.50	14.01	11.00	95.31	21.48	14.63	10.66	13.56	18.69	14.13	14.17	21.51	22.49	22.23
PDPK1	chr16.0002689275	-1.314	5.77E-23	8.83E-18	-1	90.53	91.19	9.81	8.10	93.73	2.79	12.40	92.82	4.49	6.07	4.01	90.84	91.77	10.88	92.43	1.39	2.08	4.28	9.14	5.56	4.99	3.55	5.91	11.39	6.81	9.18	92.01	11.81	7.00	93.62	9.19	5.49
TNFRSF1A	chr12.0006446797	1.832	6.91E-23	9.62E-18	+1	8.85	12.31	4.04	1.36	9.68	0.34	5.69	3.48	1.00	9.64	2.91	3.07	0.47	6.06	5.51	8.24	3.00	24.08	9.56	99.20	100.05	5.35	5.70	4.79	2.81	5.70	6.93	5.75	3.15	2.59	7.28	13.40
OCA2	chr15.0028343785	-1.241	7.02E-22	8.96E-17	-1	96.18	10.71	96.09	9.57	95.30	10.33	93.74	96.08	17.33	10.91	11.70	12.82	8.56	13.31	95.55	94.55	7.71	95.85	8.97	11.20	8.07	9.84	16.48	8.40	95.27	5.88	13.19	7.33	11.04	9.04	7.35	10.39
DLGAP1	chr18.0004068725	1.295	1.43E-21	1.60E-16	+1	23.56	18.99	18.00	14.71	13.76	21.09	11.73	15.80	11.42	15.44	11.29	12.32	20.03	11.52	23.23	20.26	93.11	96.05	17.71	21.09	21.40	11.18	19.42	16.83	10.46	16.72	11.81	93.99	16.58	90.43	18.73	93.92
LPA	chr6.0161033995	1.101	1.46E-21	1.60E-16	+1	100.05	0.43	7.49	13.76	4.71	98.45	6.00	99.66	5.68	100.05	5.91	6.29	4.38	7.21	100.05	9.76	14.62	5.29	5.60	100.05	100.05	6.72	99.50	100.05	98.84	100.04	99.96	98.49	98.56	99.47	99.69	99.20
WNT5B	chr12.0001704443	-1.235	1.85E-21	1.89E-16	-1	36.39	28.62	19.64	30.46	96.01	22.52	95.35	96.69	21.08	95.66	23.17	22.75	17.65	25.74	92.27	29.80	19.33	22.95	24.46	18.06	24.49	19.81	20.92	18.23	17.59	19.17	22.82	14.43	18.44	19.27	18.09	21.37
SPON1	chr11.0013984067	1.246	4.72E-21	4.52E-16	+1	13.25	9.21	8.71	14.19	16.90	16.59	97.30	13.30	10.71	12.35	15.65	14.70	9.83	14.25	14.60	12.85	6.54	14.93	97.81	13.56	95.54	97.44	7.08	97.26	10.81	10.84	8.14	15.34	20.04	97.17	98.13	10.94
CXXC11	chr2.0243078103	1.489	1.56E-20	1.40E-15	+1	7.17	10.58	10.11	7.12	6.93	7.83	6.19	9.36	7.88	11.67	10.38	8.82	10.52	10.41	9.20	7.79	97.62	5.27	5.27	1.49	7.10	8.79	8.33	97.50	8.80	12.62	98.27	11.81	11.52	11.91	7.08	9.36
ZNF90	chr19.0020188618	1.139	1.97E-20	1.68E-15	+1	33.18	25.62	26.81	21.02	23.95	24.38	23.86	12.51	24.15	31.48	24.29	27.40	25.63	36.11	22.17	30.38	17.60	33.65	91.98	90.91	17.71	91.00	23.05	20.92	91.57	20.50	90.95	94.41	21.61	24.01	92.23	90.81
THBS2	chr6.0169653176	-1.144	2.25E-20	1.81E-15	-1	32.20	26.20	29.06	23.21	93.65	90.17	26.71	19.14	93.94	92.65	31.28	27.09	91.19	90.43	92.88	28.78	23.34	22.87	42.12	28.66	23.40	17.51	21.10	19.37	19.70	26.99	21.13	30.51	17.14	17.84	33.55	36.16
RIOK1	chr6.0007394523	1.109	2.38E-20	1.82E-15	+1	19.15	24.93	19.56	18.44	25.51	23.03	17.87	12.62	86.01	15.78	19.86	36.04	19.78	21.29	17.94	89.16	24.32	21.86	90.55	87.24	88.97	20.36	90.30	87.68	88.93	22.85	23.80	87.30	81.36	85.49	90.62	18.44
NA	chr14.0037053607	-1.086	2.56E-20	1.87E-15	-1	92.17	92.43	93.33	19.56	94.81	90.54	91.25	17.14	18.00	94.23	93.31	93.90	92.04	13.49	14.31	94.04	18.58	14.51	17.07	19.25	22.71	21.55	10.99	17.30	92.83	16.09	29.04	11.94	94.86	93.92	17.05	22.23
NA	chr11.0009635202	1.138	6.55E-20	4.56E-15	+1	2.56	10.59	100.05	4.81	12.44	4.48	4.81	5.62	2.09	14.07	98.53	8.94	2.65	8.11	10.92	98.81	99.25	19.62	99.20	99.35	9.90	98.54	5.21	10.85	5.84	99.58	9.62	4.88	98.87	3.48	99.90	95.93
NA	chr19.0048414587	-1.171	7.97E-20	5.31E-15	-1	22.87	22.66	21.24	93.13	22.58	24.63	97.03	11.67	17.75	94.82	94.59	18.26	95.63	17.43	95.15	15.47	6.88	13.97	17.92	15.64	8.88	9.28	17.78	12.02	21.70	15.66	21.92	20.17	24.33	92.35	20.73	19.80
VGLL2	chr6.0117587149	1.155	9.40E-20	5.81E-15	+1	27.66	17.53	21.78	21.28	14.59	17.77	19.80	18.53	24.64	23.71	22.96	31.40	20.22	25.87	26.36	23.53	94.77	17.53	19.10	22.80	32.65	96.06	23.13	23.40	95.82	20.47	92.46	94.99	14.25	24.74	93.96	33.41
CYP27B1	chr12.0058160354	1.255	9.49E-20	5.81E-15	+1	22.45	30.09	7.41	15.02	14.29	11.89	16.02	14.80	9.68	21.71	9.65	14.84	15.28	14.22	14.62	18.66	13.02	19.97	25.33	8.47	17.10	98.56	97.24	97.98	14.72	17.69	16.02	13.51	14.52	22.65	27.10	97.95
PDS5B	chr13.0033220266	-1.176	2.83E-19	1.67E-14	-1	10.44	94.00	11.81	94.38	24.48	96.54	18.87	94.06	19.62	93.96	91.61	13.91	13.17	16.49	8.91	6.60	16.16	17.00	10.76	12.41	7.21	15.83	11.61	13.88	18.67	19.16	7.53	11.19	16.00	95.35	18.59	21.76
DCP1B	chr12.0002113367	-1.073	5.20E-19	2.95E-14	-1	14.58	96.70	10.35	16.87	97.92	97.67	98.37	97.89	13.74	96.98	8.15	96.30	15.84	21.12	96.20	96.87	13.12	14.30	96.74	13.16	9.07	97.68	12.31	95.94	11.16	12.39	14.23	15.40	9.74	18.52	14.92	14.61
GAS6	chr13.0114547107	-1.060	5.48E-19	2.99E-14	-1	92.27	95.48	29.36	31.95	92.96	40.46	95.75	36.37	94.09	43.48	91.11	93.46	36.56	30.07	28.54	92.20	24.53	34.50	26.58	24.84	28.81	33.83	25.60	32.31	35.09	27.42	32.88	37.93	28.42	29.77	37.34	30.64
NA	chr6.0157748725	1.040	5.78E-19	3.05E-14	+1	37.91	17.28	32.01	24.99	89.20	21.33	24.99	22.78	32.29	89.95	22.94	27.06	24.39	21.95	28.75	22.94	84.65	40.57	26.08	28.70	87.48	84.44	89.38	87.68	86.76	87.60	27.74	87.30	88.76	91.47	29.75	86.75
NA	chr6.0027440945	1.221	6.21E-19	3.17E-14	+1	19.31	22.53	17.55	14.38	16.46	13.99	17.87	14.64	12.65	14.00	14.57	20.38	12.28	16.18	15.75	15.25	16.35	14.74	20.27	17.97	22.90	11.80	23.13	12.80	17.87	97.62	27.02	96.18	18.22	96.79	15.41	95.23
NA	chr15.0022665366	1.581	1.15E-18	5.66E-14	+1	6.27	8.60	6.04	10.21	2.80	4.60	3.87	9.53	12.87	8.91	10.65	0.05	0.05	5.47	9.24	0.05	7.99	13.16	6.15	96.63	5.26	9.99	12.12	2.81	8.36	6.84	3.31	15.75	3.61	2.78	7.70	98.60
B4GALT6	chr18.0029214163	1.164	1.56E-18	7.46E-14	+1	27.21	11.78	17.43	17.12	17.37	18.17	11.06	20.57	21.35	17.78	14.44	21.30	22.14	19.18	28.09	15.62	15.40	91.47	92.34	18.27	92.17	22.05	25.04	25.71	14.35	17.64	14.77	22.38	94.03	90.88	26.69	13.22
NA	chr11.0060849902	-0.997	4.78E-18	2.22E-13	-1	91.05	33.91	33.12	89.97	38.04	91.83	91.62	89.64	35.32	53.27	87.46	92.34	86.33	92.74	91.63	85.20	34.07	37.95	40.78	34.72	38.27	37.95	40.20	32.37	35.90	30.98	33.64	44.68	25.81	36.07	48.36	40.99
NA	chr8.0009755180	-1.007	5.31E-18	2.39E-13	-1	89.91	34.39	22.01	92.37	89.86	18.17	90.01	90.90	90.66	89.31	24.19	90.91	90.44	19.52	92.29	92.46	22.98	24.14	19.75	16.16	24.38	21.40	20.05	17.47	22.66	91.80	91.20	91.34	22.25	24.44	16.60	28.93
DHCR7	chr11.0071160096	-1.103	5.61E-18	2.45E-13	-1	93.64	15.55	16.30	17.00	22.53	96.18	20.18	15.96	96.14	95.71	26.34	18.83	15.91	96.05	21.72	94.60	14.28	23.41	21.90	28.61	14.73	14.59	14.74	12.33	19.65	20.88	11.79	95.58	21.74	12.76	15.05	12.29
MCF2L2	chr3.0182928999	1.073	1.10E-17	4.68E-13	+1	30.58	25.71	27.29	26.14	20.78	22.08	29.92	18.28	24.23	31.69	21.05	17.75	23.17	23.26	18.35	26.68	21.06	20.94	22.89	28.75	93.29	92.20	21.57	94.91	93.11	92.83	93.02	28.73	27.55	26.43	27.37	32.68
NA	chr22.0039323603	-1.057	1.14E-17	4.72E-13	-1	29.46	16.62	27.95	91.74	14.36	93.86	93.81	29.42	92.22	23.52	27.03	90.88	92.68	26.47	90.35	21.37	34.79	23.58	20.70	29.58	21.61	22.26	26.14	32.62	17.14	18.15	29.16	37.69	26.16	26.58	24.01	23.33
ULBP1	chr6.0150284688	1.203	1.68E-17	6.75E-13	+1	16.47	9.27	19.86	15.16	18.68	10.28	7.69	11.93	15.62	13.25	10.94	21.94	14.37	14.77	18.12	15.41	95.61	95.56	13.29	13.18	93.35	10.72	17.97	11.30	19.70	16.23	15.62	8.51	10.73	10.78	13.85	94.89
NA	chr6.0028715621	1.059	3.38E-17	1.33E-12	+1	32.20	19.94	29.06	30.03	25.29	20.13	32.61	20.52	22.70	17.87	19.09	19.22	19.66	19.89	23.41	32.26	90.45	16.74	94.25	91.83	92.22	25.39	88.38	29.24	22.68	29.64	93.09	34.05	17.97	18.10	24.81	33.94
BCL7C	chr16.0030886974	-1.148	3.56E-17	1.35E-12	-1	12.05	89.10	12.78	14.81	13.37	10.29	21.81	15.36	91.24	12.51	90.54	90.04	13.79	12.73	12.99	89.27	7.72	13.28	11.22	8.77	15.02	11.81	9.92	91.29	18.03	15.73	13.18	7.28	15.62	9.07	6.56	16.57
C11orf54	chr11.0093475384	1.070	3.62E-17	1.35E-12	+1	26.77	21.82	18.56	27.85	24.04	20.51	13.03	16.32	12.88	24.27	27.93	26.93	17.25	31.28	23.44	32.64	11.14	25.41	33.14	96.56	31.13	67.32	27.46	28.85	18.08	96.85	67.42	28.41	68.27	71.56	66.81	31.92
ALOX12B	chr17.0007975795	1.183	4.90E-17	1.79E-12	+1	17.62	15.75	12.75	12.19	12.90	8.23	10.37	12.37	20.51	11.59	12.19	10.86	22.00	26.11	16.34	15.66	15.55	7.49	32.13	95.02	93.75	93.94	2.74	13.09	7.83	9.28	8.27	15.07	17.15	94.32	27.32	8.78
RPS6KA2	chr6.0167001337	1.092	5.19E-17	1.85E-12	+1	23.62	14.29	27.47	22.43	24.25	18.15	18.62	17.27	18.53	22.44	13.35	28.89	13.37	21.64	19.65	23.71	92.93	92.71	92.62	21.30	23.79	13.62	19.56	92.05	29.50	22.91	22.64	22.14	15.80	21.02	27.10	91.75
NA	chr15.0099143762	-1.002	5.63E-17	1.96E-12	-1	91.84	92.90	91.03	27.29	92.86	15.11	90.20	93.89	92.44	19.46	21.30	26.45	20.59	18.01	91.91	91.58	17.25	18.58	17.92	24.41	24.85	21.37	22.97	20.31	25.35	15.86	92.66	23.66	95.60	24.81	18.37	23.35
GGA1	chr22.0038022735	0.974	6.12E-17	2.08E-12	+1	31.45	26.77	41.68	36.70	22.38	27.85	89.73	26.21	39.47	23.82	25.60	33.31	32.86	41.97	36.30	25.99	88.47	88.81	89.31	33.38	89.58	33.98	36.13	90.69	88.89	91.96	92.96	90.73	36.08	33.15	87.08	35.66
NA	chr20.0056056314	1.053	7.10E-17	2.33E-12	+1	29.88	16.66	13.59	26.92	11.62	17.15	21.68	15.69	19.89	72.21	34.16	28.48	17.34	20.00	24.47	8.04	71.97	97.17	33.59	14.83	27.67	12.47	25.99	30.80	73.86	96.38	95.71	68.56	17.34	19.82	28.36	72.36
PWP1	chr12.0108079458	-1.263	7.14E-17	2.33E-12	-1	17.83	96.32	8.46	9.49	9.68	10.21	13.25	5.92	98.78	12.55	8.88	11.91	96.84	12.42	12.10	15.62	10.73	13.07	12.25	12.63	12.69	13.14	12.61	7.72	18.67	12.90	9.33	10.27	9.36	4.50	7.90	14.61
BET1L	chr11.0000188098	-0.938	8.48E-17	2.70E-12	-1	100.05	95.65	92.86	24.19	98.12	13.73	99.87	93.76	99.55	99.89	86.43	97.96	8.06	98.48	92.76	100.05	5.27	0.05	12.33	18.50	0.67	7.73	99.01	3.47	98.79	14.23	99.18	100.05	17.65	4.20	97.91	100.05
ZIC1	chr3.0147130343	-1.044	1.09E-16	3.41E-12	-1	25.62	94.69	19.74	91.54	16.22	91.82	22.72	95.03	20.62	18.49	92.09	36.16	23.88	22.91	93.86	24.50	32.27	18.05	29.40	21.46	25.05	25.94	22.26	20.07	19.97	22.29	34.76	24.53	25.44	18.22	23.50	19.34
NA	chr20.0049598101	-1.050	1.26E-16	3.85E-12	-1	7.96	19.76	14.85	94.49	22.30	95.17	29.54	17.07	95.62	17.53	98.36	94.81	17.34	27.00	96.65	17.73	15.34	18.97	13.61	15.93	20.67	20.74	15.93	15.09	23.09	18.35	94.82	10.30	19.77	18.90	19.27	28.88
DLEU2	chr13.0050656618	-1.397	1.37E-16	4.13E-12	-1	16.53	3.69	100.05	7.20	10.01	13.78	9.85	5.33	6.96	95.67	14.44	2.13	13.55	11.47	14.13	6.60	6.63	8.57	15.53	5.50	6.79	4.41	3.13	12.36	5.39	5.11	5.25	5.60	9.68	10.51	12.21	7.97
LMLN	chr3.0197687858	-1.014	1.48E-16	4.36E-12	-1	92.33	95.59	20.83	8.72	96.13	93.52	93.37	15.04	14.17	95.55	93.27	14.87	93.07	10.49	15.86	18.22	14.33	16.53	16.00	20.40	94.01	22.59	10.29	16.46	16.00	19.41	18.21	16.42	16.75	13.75	93.46	25.96
UST	chr6.0149354426	0.950	1.69E-16	4.88E-12	+1	32.39	23.21	25.86	25.86	21.86	16.57	24.73	92.62	92.99	24.85	23.80	27.48	21.40	19.93	28.79	92.46	92.41	92.92	92.03	91.33	92.02	29.92	30.41	21.15	94.41	27.16	24.65	90.81	92.98	94.34	93.96	91.40
H1F3	chr6.0026235050	1.060	1.87E-16	5.30E-12	+1	20.18	8.20	14.85	16.69	14.85	92.99	25.69	9.54	18.14	23.57	16.35	21.94	16.60	14.25	17.43	14.10	17.57	19.31	18.19	13.18	93.21	16.05	92.86	10.88	95.44	16.32	19.08	14.81	12.46	95.31	95.72	92.95
SHISA6	chr17.0011145564	-0.921	1.97E-16	5.45E-12	-1	93.60	91.27	92.78	91.25	20.61	91.37	93.58	91.97	92.89	22.32	92.96	91.13	94.83	92.91	92.03	91.33	22.68	20.31	21.61	17.37	90.21	19.62	20.93	27.34	31.61	95.87	92.81	92.31	15.51	30.59	17.44	89.93
NA	chr16.0003200077	-1.079	1.99E-16	5.45E-12	-1	16.51	20.09	21.36	15.86	20.28	19.07	32.52	91.27	18.49	89.73	88.18	23.99	88.09	21.30	22.82	87.31	25.87	18.87	25.84	16.90	22.68	15.44	13.96	26.87	18.64	17.41	17.90	26.94	12.86	16.39	19.49	23.67
NA	chr13.0053025394	-1.015	2.10E-16	5.64E-12	-1	86.05	84.43	90.91	21.21	22.32	14.65	88.45	16.03	17.77	91.12	87.72	18.05	14.44	13.02	92.13	90.57	17.57	17.24	18.16	18.22	89.04	14.55	12.48	23.39	20.36	17.56	87.31	14.32	18.68	17.17	19.92	13.45
NA	chr18.0032013488	0.968	2.41E-16	6.36E-12	+1	30.17	39.30	22.91	22.38	20.60	19.16	46.21	31.28	23.03	91.21	29.00	29.64	38.02	30.45	30.38	22.09	90.87	93.05	29.28	92.87	31.17	23.69	90.12	29.24	92.30	92.31	24.68	90.58	90.81	90.88	28.80	40.07
DNAJA1	chr9.0033025770	-0.922	3.27E-16	8.48E-12	-1	92.09	91.60	93.42	93.90	24.83	93.60	25.01	92.94	24.73	94.15	91.95	93.19	89.90	89.26	91.69	91.09	24.15	31.92	93.19	25.32	26.50	92.11	22.83	24.58	22.54	28.54	92.53	21.83	24.84	27.86	21.65	91.77
NA	chr17.0007738069	-1.044	3.97E-16	1.01E-11	-1	91.98	87.04	23.61	92.07	20.00	90.27	21.65	24.74	19.03	15.69	25.00	32.70	94.58	41.55	24.86	26.13	27.74	18.26	22.11	16.19	27.53	22.90	17.96	17.97	20.50	17.76	24.95	26.09	23.43	25.89	19.37	25.30
RSRC2	chr12.0123009847	-0.974	4.24E-16	1.06E-11	-1	93.01	18.55	94.87	21.99	41.15	30.33	35.61	53.60	28.61	48.81	18.91	91.05	94.66	96.01	96.36	95.75	97.17	33.32	21.62	21.15	26.16	25.23	27.45	28.85	23.38	26.90	22.53	20.73	37.15	21.86	36.61	17.57
IMP3	chr15.0075932985	0.941	4.69E-16	1.16E-11	+1	96.05	96.40	11.83	10.21	95.38	9.73	4.35	5.22	12.28	9.83	9.79	11.10	5.17	96.57	97.25	10.20	98.10	12.02	97.21	15.64	95.87	12.39	8.88	95.42	96.03	8.64	98.04	96.36	98.23	95.72	96.18	94.62
PIWIL4	chr11.0094276724	1.042	6.15E-16	1.49E-11	+1	16.50	24.26	26.59	25.24	24.97	18.82	18.35	18.12	16.67	14.52	19.23	24.26	18.55	18.44	34.09	28.98	16.57	93.21	29.00	21.25	32.34	93.32	17.94	23.15	17.78	20.17	19.64	91.57	93.29	89.59	29.37	30.18

Gene Annotation	Chromosome Position	Log Fold Change	p-value	p(FDR)	Methylation Up/Down in CP	Control																CP															
						xx039	x053	x056	x057	x370	x380	x414	x416	x425	x431	x436	x466	x489	x527	x563	x584	x010	x028	x045	x050	x375	x376	x417	x440	x444	x533	x568	x573	x583	x597	x600	x606
DLGAP1	chr18.0003771925	-1.025	7.44E-16	1.78E-11	-1	23.55	15.94	26.05	92.33	33.72	91.18	17.87	12.25	14.54	12.89	94.47	91.78	14.43	94.25	96.97	19.22	22.18	22.80	16.91	13.03	13.28	15.18	15.40	19.70	11.27	18.12	94.32	16.58	12.43	34.88	19.48	23.47
NA	chr22.0050977744	0.934	7.71E-16	1.81E-11	+1	23.14	15.53	19.25	92.88	16.43	21.97	14.35	11.96	9.89	16.09	16.46	92.11	93.61	21.67	92.69	23.85	93.33	95.08	18.53	14.00	93.55	20.11	96.06	92.71	14.67	91.79	94.97	94.45	93.90	94.56	16.17	91.20
TSGA13	chr7.0130353922	-0.959	7.81E-16	1.81E-11	-1	90.35	30.23	89.49	34.07	36.70	30.68	88.06	34.82	26.86	90.46	89.41	90.28	89.94	35.50	87.48	39.33	27.41	28.64	34.49	29.45	25.84	35.95	25.61	34.94	33.41	30.25	40.53	31.28	32.86	26.09	28.07	40.74
NA	chr6.0027585764	1.019	8.42E-16	1.92E-11	+1	29.26	17.44	24.58	28.86	26.94	21.22	23.83	18.69	31.66	26.49	23.46	29.23	20.63	21.89	17.43	14.10	26.95	28.51	25.02	91.83	54.66	21.57	27.08	91.66	91.77	25.49	91.47	21.61	21.97	91.79	33.55	19.37
NA	chr14.0067879115	0.906	9.13E-16	2.05E-11	+1	31.98	27.95	20.63	93.46	28.96	23.41	21.03	25.79	27.07	92.57	92.03	30.72	28.26	92.59	23.21	25.39	93.75	93.34	90.44	89.48	92.40	91.98	94.40	91.55	91.94	30.36	91.49	93.96	32.89	92.28	28.72	87.84
NA	chr6.0170452742	0.930	9.79E-16	2.17E-11	+1	39.64	38.30	28.53	27.23	41.79	27.98	37.11	29.68	92.99	23.98	31.84	36.26	22.60	37.87	40.93	27.12	93.06	25.30	91.70	91.33	43.91	93.82	91.87	28.78	91.46	94.52	30.60	91.95	91.62	32.48	90.62	31.95
NA	chr3.0196184550	-0.969	1.32E-15	2.89E-11	-1	92.33	96.17	18.74	95.56	96.69	21.13	20.98	12.66	94.74	95.18	18.00	29.28	92.59	9.03	95.46	15.33	19.48	19.10	19.64	93.34	12.54	24.48	13.94	19.88	21.82	23.32	16.65	94.57	21.79	13.25	26.76	20.30
NA	chr17.0036581506	-0.965	2.26E-15	4.88E-11	-1	88.81	90.27	28.69	20.92	21.09	91.56	92.39	91.01	92.64	26.81	30.33	90.65	30.98	35.01	24.64	30.72	30.62	27.63	33.44	18.01	22.21	24.85	23.73	26.29	24.31	30.10	29.14	38.52	27.37	42.23	23.56	31.66
NA	chr6.0159572466	-0.965	2.58E-15	5.49E-11	-1	87.95	12.90	21.72	94.24	24.20	94.83	93.49	14.22	95.83	18.93	11.37	93.32	94.04	12.78	91.86	14.74	16.76	12.98	16.65	20.50	23.40	18.28	17.62	92.40	16.64	93.79	25.22	20.64	19.82	15.79	18.87	19.21
NA	chr3.0019188810	-0.928	2.69E-15	5.64E-11	-1	17.62	92.17	94.68	14.46	18.57	92.96	93.12	20.82	97.24	15.71	92.80	90.35	90.68	94.77	16.09	96.75	32.22	18.60	95.63	23.18	19.12	92.46	11.31	15.61	22.44	13.20	22.39	21.36	24.96	20.94	18.48	93.87
RP11-595B24.2	chr18.0019674041	0.917	4.22E-15	8.73E-11	+1	33.83	23.36	40.77	35.37	38.49	39.45	43.46	38.07	25.28	23.37	20.37	24.43	22.32	23.19	41.96	87.22	90.87	92.65	72.69	88.70	88.05	32.49	90.44	91.64	92.87	23.39	32.40	35.55	25.70	31.38	87.22	85.43
CABP1	chr12.0121082579	-0.944	4.47E-15	9.13E-11	-1	94.80	31.17	21.74	27.64	29.96	31.53	35.20	34.95	23.76	93.78	95.00	25.54	92.70	95.44	94.15	95.54	23.25	30.21	37.82	27.68	29.11	31.33	27.75	35.47	27.08	33.52	22.95	31.44	25.43	28.36	31.03	36.75
NA	chr14.0052732100	0.978	4.85E-15	9.77E-11	+1	17.78	14.73	93.04	20.16	95.88	15.85	15.15	15.60	13.93	20.70	16.93	18.67	10.52	11.78	12.98	9.58	95.54	13.54	10.66	93.52	14.93	19.80	13.03	11.83	95.30	19.98	92.40	96.30	14.17	92.00	19.94	94.12
TEX30	chr13.0103425377	-1.050	5.76E-15	1.15E-10	-1	24.29	98.96	21.86	24.40	17.30	97.48	13.12	14.21	13.15	16.60	16.31	97.33	19.12	13.41	97.93	22.75	15.40	15.91	19.05	20.06	18.06	15.91	13.57	19.39	20.56	11.84	27.25	14.32	22.07	16.68	19.78	23.10
NA	chr3.0041159255	0.966	6.49E-15	1.27E-10	+1	90.33	18.10	20.42	17.32	28.66	25.65	23.66	20.56	16.10	23.36	23.51	22.36	20.42	22.03	19.19	26.15	25.01	19.84	23.62	35.59	19.98	93.75	92.94	32.26	88.85	20.38	90.42	92.27	29.15	93.99	35.57	25.56
RAP1B	chr12.0069005196	-0.856	7.09E-15	1.36E-10	-1	92.04	97.55	96.43	94.51	94.90	94.71	93.87	28.07	93.88	95.66	95.86	94.63	94.66	95.25	94.87	97.25	23.68	33.44	15.43	12.63	93.85	26.47	94.00	96.11	93.42	20.38	30.88	97.34	19.76	16.44	95.32	34.18
NA	chr7.0100946325	-1.042	7.13E-15	1.36E-10	-1	91.04	18.59	27.53	17.73	23.02	92.11	21.67	93.77	15.51	8.27	17.68	18.83	30.51	22.73	91.53	25.68	17.31	9.77	11.37	19.28	13.80	18.37	12.89	26.54	29.46	20.44	25.83	17.53	14.77	15.55	19.97	25.77
TRERF1	chr6.0042418413	0.925	7.64E-15	1.44E-10	+1	93.61	16.60	21.72	16.69	16.18	15.48	18.60	19.35	15.62	92.65	16.94	23.23	17.21	14.77	93.06	19.19	18.98	94.94	21.76	94.35	93.49	95.82	17.18	93.18	93.69	19.10	93.96	12.00	13.39	20.17	95.85	94.52
NA	chr13.0095202503	0.885	1.03E-14	1.93E-10	+1	88.98	18.84	24.34	20.51	18.65	30.06	21.64	27.64	15.30	93.28	24.17	92.72	24.76	15.48	19.14	94.41	21.37	19.91	94.69	92.32	92.90	94.69	93.70	94.60	96.13	93.70	95.36	25.75	25.87	95.16	37.68	92.56
NA	chr6.0087319684	0.885	1.07E-14	1.97E-10	+1	87.22	27.68	65.95	30.52	26.80	29.60	31.61	91.47	26.91	43.02	32.79	22.50	23.95	38.94	29.43	19.19	89.69	91.98	20.22	90.75	41.92	23.95	93.01	41.60	91.75	90.66	92.00	89.15	86.05	90.34	45.10	84.10
FAM201B	chr2.0133110495	0.962	1.16E-14	2.09E-10	+1	12.64	13.40	18.19	95.90	15.94	14.59	16.03	15.44	11.61	10.50	14.36	13.82	96.12	21.84	19.16	19.88	11.24	15.68	13.39	12.90	11.82	96.24	97.19	12.55	97.03	97.32	96.36	97.02	93.53	20.71	12.79	15.01
MYO18A	chr17.0027482390	0.928	1.16E-14	2.09E-10	+1	88.23	41.05	21.11	26.21	28.77	34.70	13.35	22.82	23.99	34.74	27.77	13.81	20.08	24.67	37.55	27.44	89.84	24.70	39.90	85.59	86.35	26.30	86.83	88.56	19.47	28.54	11.76	35.84	86.69	87.36	41.51	88.37
NDUFA9	chr12.0004758511	-0.864	1.55E-14	2.76E-10	-1	92.04	89.59	90.22	37.33	90.11	86.96	91.67	91.72	91.22	88.85	88.60	89.08	25.62	91.17	33.29	94.70	37.00	34.55	30.57	31.15	35.59	89.20	36.55	37.33	46.40	27.04	32.54	45.56	33.89	91.15	48.96	41.06
LINC00403	chr13.0112714769	-1.032	1.61E-14	2.83E-10	-1	23.37	16.55	19.85	11.49	18.78	20.53	97.27	95.39	21.35	23.46	92.06	93.98	15.41	16.49	21.18	17.64	22.26	13.21	20.90	24.87	21.82	11.82	18.69	18.72	22.55	13.97	11.27	24.69	26.84	10.51	17.84	14.96
SYN3	chr22.0033197965	0.945	1.76E-14	3.06E-10	+1	16.40	15.59	15.69	11.95	20.76	22.00	17.96	95.92	14.23	12.18	15.46	20.77	19.42	93.76	21.72	17.94	94.95	95.89	16.87	97.13	15.16	14.81	26.96	95.92	15.48	95.93	17.50	21.27	95.41	15.47	18.91	96.36
KISS1R	chr19.0000920756	0.906	1.89E-14	3.24E-10	+1	37.37	44.03	34.64	28.92	36.51	31.70	31.98	25.62	29.13	37.88	32.31	35.89	33.90	29.75	33.63	24.19	89.91	93.21	36.55	29.00	23.37	28.62	92.71	33.20	30.56	92.23	91.44	93.23	93.06	30.44	92.55	40.99
TBC1D3	chr17.0036286187	-0.893	2.01E-14	3.42E-10	-1	33.61	95.80	24.70	96.01	25.98	95.96	30.18	94.74	27.76	27.80	94.82	93.60	96.00	96.07	25.64	94.50	18.03	11.97	33.57	23.60	21.91	22.72	93.93	24.92	95.83	25.93	16.85	30.16	34.98	68.97	31.16	12.51
NA	chr13.0027075176	-0.918	2.51E-14	4.22E-10	-1	91.57	13.91	22.33	18.37	96.25	22.74	20.35	93.86	95.24	28.21	90.24	92.35	20.82	16.97	94.10	93.78	22.15	23.79	16.07	26.53	17.80	16.94	14.78	22.13	93.94	19.05	25.25	19.71	94.12	19.15	26.78	23.44
ANKRD36C	chr2.0096657644	1.153	2.73E-14	4.54E-10	+1	7.85	9.91	13.15	11.70	13.90	9.85	1.86	10.21	10.62	8.90	7.30	17.25	16.28	16.03	15.64	17.27	7.58	6.72	14.39	6.79	9.51	8.74	12.09	95.78	98.21	3.06	14.86	96.46	11.26	12.99	11.46	10.82
PCDHGA1	chr5.0140782367	-0.909	3.48E-14	5.72E-10	-1	93.33	20.66	22.22	92.70	93.85	92.36	24.78	20.37	94.55	93.39	23.98	91.99	24.36	22.38	92.33	23.02	16.47	23.91	23.25	23.75	22.58	23.19	24.22	20.73	20.23	94.88	91.39	19.60	17.35	13.76	21.85	35.77
NA	chr13.0100610016	-0.987	3.58E-14	5.83E-10	-1	19.14	96.97	16.21	11.66	18.78	20.57	96.57	14.53	95.38	22.09	93.51	14.48	15.41	16.97	21.25	97.12	17.10	18.25	96.12	23.45	18.82	16.94	12.01	21.76	19.12	11.71	10.50	15.82	17.28	7.96	15.76	14.96
SOX4	chr6.0021594873	-0.879	3.64E-14	5.86E-10	-1	91.15	92.53	94.52	93.25	25.84	91.31	92.42	26.19	31.34	24.26	31.84	95.05	88.32	20.15	89.54	90.68	30.55	25.50	37.38	33.26	30.62	26.73	93.28	28.07	24.08	28.86	88.90	27.75	27.33	26.95	27.55	29.21
C20orf197	chr20.0058801846	-0.846	3.79E-14	6.04E-10	-1	24.29	93.40	94.76	96.47	97.06	95.62	97.28	95.54	91.03	27.14	95.43	96.36	95.43	16.53	96.45	95.69	21.89	24.99	11.29	96.09	24.68	20.53	17.15	95.17	21.76	94.79	16.57	96.81	26.31	21.83	95.24	42.48
NA	chr13.0028371410	-0.871	3.86E-14	6.09E-10	-1	91.05	11.80	95.01	18.57	96.83	18.41	20.65	94.25	15.48	92.18	94.09	94.76	93.44	93.26	96.18	93.78	33.40	18.25	24.32	96.26	94.45	17.17	14.15	25.46	17.60	13.18	20.26	17.09	18.42	13.03	95.56	92.88
GPC6	chr13.0093896533	-1.044	4.03E-14	6.29E-10	-1	92.87	5.66	22.38	22.80	14.08	96.54	8.16	10.30	4.22	18.22	17.83	19.35	11.16	19.02	95.96	93.05	12.10	22.72	19.92	20.63	11.49	7.74	15.44	15.77	15.53	13.06	16.90	8.85	17.46	18.43	28.08	22.77
NPIPA3	chr16.0014818378	0.904	5.29E-14	8.18E-10	+1	19.90	17.94	21.72	24.70	22.99	16.08	24.28	25.42	22.17	62.09	91.78	61.69	24.84	18.71	21.73	14.28	16.28	92.54	23.34	21.99	61.89	60.47	18.19	91.12	92.39	94.49	91.26	91.78	29.59	24.30	94.00	16.76
CNGB1	chr16.0058000211	-0.871	6.21E-14	9.50E-10	-1	86.37	24.86	88.09	87.46	88.45	22.68	87.12	88.42	17.84	89.07	88.33	88.37	88.09	19.35	25.09	83.33	30.67	20.78	87.26	24.79	27.31	21.31	21.98	30.68	21.77	90.70	25.28	33.74	24.23	21.26	88.62	15.65
TTC9B	chr19.0040723542	-0.889	8.47E-14	1.28E-09	-1	93.40	90.58	26.83	88.39	26.65	88.57	36.84	16.05	91.15	27.35	33.47	88.93	90.87	30.33	29.23	90.05	22.73	29.63	25.89	26.08	23.84	27.51	28.12	26.67	29.30	34.95	33.23	28.34	24.49	88.28	25.70	36.99
BC043355	chr2.0056193463	0.841	9.10E-14	1.37E-09	+1	29.77	29.01	91.96	29.74	30.63	29.46	31.06	34.59	33.61	31.34	35.19	34.52	92.43	32.90	32.34	92.90	31.80	38.38	32.51	93.60	92.38	90.93	35.36	91.76	91.17	92.45	93.29	92.03	91.76	91.26	92.42	90.25
GALNT9	chr12.0132855014	-0.887	9.33E-14	1.39E-09	-1	34.16	32.41	92.80	95.28	92.86	93.17	34.31	92.12	28.97	34.93	46.85	83.18	31.34	91.17	36.33	31.13	27.67	32.79	38.59	33.66	33.45	32.99	27.08	39.87	37.00	27.33	18.31	31.20	29.43	29.84	34.74	40.01
NKD1	chr16.0050582980	-0.842	9.77E-14	1.44E-09	-1	83.22	26.20	88.09	90.01	88.00	27.38	86.87	90.10	89.67	90.69	87.67	86.55	87.90	87.36	27.93	84.76	17.50	21.43	88.10	20.16	88.02	87.04	21.37	22.39	87.36	26.84	23.39	21.98	24.97	88.00	17.24	26.06
NA	chr18.0043335933	-0.865	1.16E-13	1.69E-09	-1	20.57	93.17	93.63	16.32	93.09	22.99	93.65	94.47	16.66	95.23	12.51	92.66	93.45	94.86	95.99	18.92	22.91	18.28	27.18	94.85	22.01	19.68	20.60	15.83	25.29	30.82	22.70	16.58	27.39	25.53	93.59	91.51
NA	chr6.0105851521	-0.935	1.41E-13	2.04E-09	-1	91.15	22.39	29.06	17.25	28.86	26.15	30.22	26.50	33.97	24.48	18.18	95.63	18.49	93.17	91.39	94.18	25.29	24.54	19.88	27.37	23.40	25.68	25.47	19.37	17.03	29.96	23.46	17.57	30.09	30.01	23.31	24.54
IFFO1	chr12.0006663682	0.873	1.75E-13	2.50E-09	+1	41.66	45.92	34.27	34.14	29.26	43.80	32.82	35.20	24.83	24.94	30.76	34.25	30.02	26.39	25.37	33.47	94.19	37.18	27.40	23.14	91.76	95.85	94.20	34.09	92.78	24.19	29.68	91.73	27.81	92.11	20.65	91.55
C20orf26	chr20.0020258001	0.939	2.19E-13	3.10E-09	+1	21.05	20.13	22.23	17.77	23.55	21.58	28.42	18.35	18.49	24.74	25.87	30.81	15.73	23.60	34.99	19.70	92.58	32.82	95.04	34.27	25.97	19.53	24.75	21.21	96.14	20.77	21.43	25.24	35.52	32.77	35.69	92.65
IGF1R	chr15.0099344316	-0.948	2.20E-13	3.10E-09	-1	91.49	24.55	15.25	18.27	91.92	94.72	22.97	18.01	20.05	18.62	17.72	93.65	92.79	23.74	18.25	19.03	14.86	11.80	20.72	16.94	13.58	19.26	22.07	17.03	92.49	17.41	18.47	13.96	18.90	14.46	25.12	15.44
PFN1	chr17.0004850081	0.893	2.42E-13	3.35E-09	+1	25.71	30.34	23.77	24.72	27.46	30.22	21.01	32.05	24.61	23.28	32.25	27.06	32.76	45.14	25.87	32.79	92.53	27.10	32.41	20.04	90.57	26.30	25.33	91.95	93.19	22.48	15.48	25.11	24.82	89.11	87.83	90.87
YWHAB	chr20.0043513649	1.197	2.43E-13	3.35E-09	+1	3.71	6.24	2.70	2.11	0.05	3.73	4.71	100.05	1.55	0.05	5.32	0.05	0.05	6.20	0.05	9.90	1.18	2.79	4.76	99.42	0.05	100.05	3.27	100.05	1.91	0.70	9.21	0.55	2.12	0.97	2.57	9.18
PDX1	chr13.0028497262	-0.879	2.64E-13	3.60E-09	-1	14.26	14.51	98.39	98.05	98.03	18.41	98.91	14.53	14.24	22.09	95.38	97.87	97.02	97.69	18.51	10.97	18.69	15.29	12.66	99.19	10.02	7.73	14.74	13.36	21.75	19.72	18.14	15.57	97.45	97.92	17.84	13.87
PAX1	chr20.0021689463	-1.020	2.70E-13	3.65E-09	-1	96.00	13.20	21.85	13.14	97.18	10.90	22.34	16.34	15.91	95.69	27.70	25.47	25.67	10.67	20.72	12.49	14.41	20.88	14.38	19.14	24.70	15.30	12.39	13.87	20.19	13.69	12.59	10.80	16.15	22.06	14.37	13.20
CEBPB	chr20.0048919181	-0.935	3.41E-13	4.57E-09	-1	18.44	19.76	18.03	22.09	18.12	95.17	95.18	93.44	18.93	21.19	93.60	93.41	23.25	14.87	25.08	23.19	21.84	18.46	27.55	26.04	20.67	17.94	22.75	19.33	28.37	21.31	21.76	21.42	22.07	23.04	24.60	25.20
LGALS2	chr22.0037977772	0.855	3.64E-13	4.84E-09	+1	24.31	91.35	23.87	18.34	19.49	22.57	21.43	22.11	15.28	25.25	13.47	17.43	92.47	28.76	91.06	22.68	26.32	22.83	93.81	93.51	91.85	20.58	95.18	22.72	93.36	92.74	95.64	24.75	16.90	92.65	88.67	25.55
NA	chr2.0134363040	-0.856	3.67E-13	4.84E-09	-1	89.22	90.78	36.62	33.90	37.55	89.71	88.94	33.84	32.50	30.30	90.66	90.19	33.57	89.60	30.35	91.30	32.05	33.81	42.51	37.18	29.40	36.24	39.47	29.82	38.84	26.37	33.77	26.35	34.02	41.05	31.17	33.94
NA	chr18.0039694525	1.048	3.85E-13	4.99E-09	+1	16.53	14.75	16.87	20.75	13.06	7.66	14.67	12.57	12.29	14.37	9.64	20.20	15.91	13.72	12.18	12.62	8.58	93.92	93.64	95.50	16.22	14.28	16.00	17.03	20.31	22.58	9.67	14.76	9.98	13.23	16.02	11.65
ZIC1	chr3.0147128690	0.852	3.85E-13	4.99E-09	+1	32.59	27.65	92.23	19.78	20.01	90.63	24.66	24.73	31.80	32.94	24.26	25.85	25.41	32.41	22.97	30.64	92.13	90.46	38.11	88.66	22.52	27.55	92.56	92.27	91.38	17.02	32.84	91.00	31.38	19.97	91.53	91.27
FOXB1	chr15.0060302054	-0.841	4.30E-13	5.53E-09	-1	89.24	36.59	38.41	13.82	87.44	89.96	86.20	29.32	87.61	33.33	87.23	88.95	87.61	86.19	31.95	87.88	17.98	18.58	28.36	34.24	15.78	83.36	89.98	26.67	23.14	21.61	28.42	16.97	90.11	20.15	51.54	25.60
NA	chr15.0084114534	-0.840	4.57E-13	5.83E-09	-1	88.62	93.36	27.64	22.38	24.81	94.08	91.36	93.10	91.22	93.35	25.16	92.18	21.42	90.66	92.80	19.88	28.61	93.21	25.77	28.56	16.99	22.07	23.94	21.74	23.36	93.05	27.32	20.44	26.45	20.15	28.43	92.97
SF3B3	chr16.0070557879	0.866	5.26E-13	6.62E-09	+1	19.37	28.59	21.22	28.19	23.38	19.61	90.07	20.80	20.55	20.97	89.04	29.45	27.84	23.64	17.19	18.39	19.66	88.74	89.27	88.86	24.37	90.88	18.74	90.42	90.75	93.01	25.84	23.77	89.73	22.61	21.95	32.39
PSD	chr10.0104179528	-0.844	5.27E-13	6.62E-09	-1	29.40	28.49	95.80	93.91	95.17	92.64	28.52	14.74	27.89	21.05	22.09	93.84	93.83	95.54	94.50	93.51	23.24	18.18	11.05	21.61	14.00	94.94	20.25	17.91	25.98	27.48	29.36	22.26	29.68	93.05	95.26	24.15
SUMF1	chr3.0004508116	0.826	5.81E-13	7.21E-09	+1	36.25	96.17	31.34	29.28	24.90	94.47	37.20	32.36	23.93	29.78	39.38	26.50	40.11	23.09	32.25	38.97	31.37	28.80	26.99	94.65	94.17	94.28	92.35	31.20	40.61	30.34	91.14	95.38	95.49	94.19	92.79	96.12
TAF10	chr11.0006633681	-0.875	5.84E-13	7.21E-09	-1	15.62	31.17	96.35	95.16	95.74	34.73	95.25	15.96	17.44	22.76	96.33	94.08	12.34	18.98	26.80	95.06	12.51	26.54	21.13	22.81	17.53	19.66	16.33	18.41	93.61	20.34	94.70	19.54	18.27	23.51	20.75	25.19
PLCB4	chr20.0009100637	0.918	6.75E-13	8.26E-09	+1	16.66	23.48	19.16	10.85	17.77	14.03	95.46	15.43	17.37	20.34	26.73	21.22	17.37	21.02	21.09	14.08	18.22	96.44	21.33	95.03	92.49	29.37	13.78	21.01	24.89	16.69	15.16	25.01	28.31	96.06	91.96	19.19
C19orf26	chr19.0001238477	-0.832	7.63E-13	9.26E-09	-1	90.43	41.18	90.42	88.55	31.40	90.70	92.43	30.26	34.69	34.44	88.89	90.16	90.44	35.33	90.66	35.68	29.81	35.02	36.19	90.24	27.42	28.43	29.23	27.30	32.38	35.24	44.58	34.01	31.77	28.80	39.99	42.19

Gene Annotation	Chromosome Position	Log Fold Change	p-value	p(FDR)	Methylation Up/Down in CP	Control																CP															
						xx039	x053	x056	x057	x370	x380	x414	x416	x425	x431	x436	x466	x489	x527	x563	x584	x010	x028	x045	x050	x375	x376	x417	x440	x444	x533	x568	x573	x583	x597	x600	x606
BMS1P21	chr10.0081665235	-0.840	7.73E-13	9.32E-09	-1	36.47	35.54	37.84	91.51	91.54	38.19	39.22	21.67	91.92	90.93	89.94	91.99	46.15	23.58	89.00	91.04	34.44	31.30	33.95	31.52	34.64	32.61	33.94	33.93	36.91	35.64	36.33	40.20	37.63	40.39	33.76	34.59
CRYL1	chr13.0021069344	-0.904	7.80E-13	9.33E-09	-1	21.13	18.84	95.41	95.55	95.75	14.83	96.75	94.94	16.15	20.85	14.79	16.90	15.41	13.53	12.88	96.16	13.97	19.39	20.58	24.87	22.84	24.62	22.26	20.17	17.42	17.57	20.26	93.72	17.83	16.77	26.19	16.44
NA	chr11.0067687636	0.824	8.53E-13	1.01E-08	+1	48.74	34.07	30.11	29.47	43.44	34.11	37.27	39.82	37.79	38.68	42.78	37.70	42.98	36.20	52.94	32.74	31.47	94.22	49.10	48.86	91.42	92.13	93.43	90.28	43.14	45.05	90.97	47.70	93.74	49.63	94.37	42.58
KCNA1	chr12.0005019351	-0.898	8.71E-13	1.03E-08	-1	29.64	28.87	27.74	98.29	21.90	21.58	69.09	25.66	20.33	70.14	68.13	95.94	98.08	23.11	23.95	29.01	27.25	26.68	29.50	27.28	24.11	25.68	23.34	29.35	27.61	19.37	22.97	30.23	20.40	26.08	17.66	25.25
LPA	chr6.0161067521	-0.902	8.90E-13	1.04E-08	-1	24.25	63.08	61.73	63.73	60.43	61.37	97.92	10.92	99.32	16.71	15.36	14.13	13.07	12.12	61.58	62.25	17.53	6.87	5.23	8.32	68.06	12.67	61.30	4.85	61.91	15.65	11.85	17.57	13.59	16.80	62.83	9.13
NA	chr12.0103974064	0.902	8.95E-13	1.04E-08	+1	25.49	23.73	96.28	18.05	20.15	18.87	20.07	21.90	17.12	27.51	19.37	15.07	17.89	15.87	18.68	17.60	94.19	97.48	25.33	93.58	27.43	23.47	22.13	18.77	28.18	25.62	22.31	94.65	18.44	29.99	92.53	23.29
EYA4	chr6.0134210544	0.886	9.49E-13	1.09E-08	+1	22.69	22.39	19.28	18.21	19.79	17.41	23.83	18.69	26.27	17.62	93.92	28.12	17.21	20.35	31.56	26.44	28.83	95.56	93.84	15.47	30.54	24.77	94.33	18.03	24.32	94.17	91.74	15.86	18.37	21.91	93.54	24.66
RP11-25L3.3	chr18.0071642546	-0.929	9.61E-13	1.10E-08	-1	21.51	94.28	94.63	14.71	94.78	91.46	9.80	21.37	15.35	22.38	18.26	13.98	17.94	96.83	15.08	13.32	12.72	19.28	11.59	16.60	20.56	15.76	17.44	11.27	20.31	22.58	7.38	13.65	19.48	20.41	21.95	92.86
NA	chr18.0012287195	-0.842	9.92E-13	1.12E-08	-1	18.57	95.51	15.12	10.61	10.84	95.90	14.22	96.47	94.82	97.34	98.32	95.46	96.59	97.41	13.50	21.33	12.11	10.21	12.65	97.33	96.77	14.00	8.60	15.80	96.85	98.74	11.47	11.34	15.69	13.59	12.87	13.70
NA	chr6.0001396193	0.807	1.04E-12	1.17E-08	+1	17.10	22.39	21.72	14.84	94.09	12.70	23.29	93.85	22.67	18.06	19.53	95.05	91.85	18.51	93.86	21.34	93.33	95.73	17.40	15.27	93.06	96.29	93.54	92.89	93.28	95.33	92.46	92.74	16.94	95.16	17.24	93.91
PTPRR	chr12.0071439895	0.860	1.11E-12	1.24E-08	+1	29.60	23.73	96.58	20.08	23.78	14.25	18.62	18.99	21.08	96.20	16.80	21.78	15.84	19.35	25.61	20.12	23.33	94.75	95.33	90.27	94.51	95.51	17.65	96.11	26.04	26.44	21.64	18.68	27.81	92.11	29.23	28.17
MEG3	chr14.0101308437	-0.931	1.38E-12	1.53E-08	-1	30.66	11.62	22.15	25.49	95.88	24.20	94.72	92.62	16.81	20.26	24.89	27.63	93.78	16.73	14.76	21.76	22.89	21.20	15.04	23.36	25.59	17.28	21.15	20.35	18.66	25.82	20.40	14.04	17.69	16.12	23.20	29.09
HDAC11	chr3.0013541769	-0.799	1.51E-12	1.66E-08	-1	89.81	87.76	88.89	86.56	90.41	91.82	92.85	91.90	34.49	93.35	90.14	90.35	31.62	29.62	88.84	32.44	31.59	46.40	40.30	44.62	32.02	40.05	89.53	31.53	37.39	26.32	32.55	43.31	44.52	88.91	32.81	33.93
LPP	chr3.0188420144	-0.865	1.55E-12	1.69E-08	-1	89.81	29.30	26.26	26.14	19.28	51.27	24.66	94.72	20.16	93.85	34.36	91.82	93.07	22.69	18.10	95.11	32.41	22.13	20.98	28.75	15.86	22.94	94.19	29.35	30.02	18.85	25.35	21.36	25.44	15.80	24.91	27.08
NA	chr15.0075072612	-0.799	1.73E-12	1.88E-08	-1	23.06	95.12	92.51	15.27	13.00	94.86	92.70	92.96	93.23	94.96	21.44	91.88	93.62	93.21	94.02	91.35	16.78	21.09	27.18	26.77	93.35	15.22	19.63	91.44	22.47	10.17	22.71	93.98	95.46	17.18	92.86	19.34
NA	chr22.0037862726	0.786	1.78E-12	1.91E-08	+1	33.08	33.83	39.15	43.26	42.70	31.39	47.59	31.20	38.73	90.34	21.65	37.63	73.72	49.26	47.22	91.87	41.06	92.72	88.83	91.06	91.31	93.42	91.51	90.36	91.81	91.22	92.96	40.25	90.49	32.52	91.27	89.47
CPNE5	chr6.0036737891	0.836	1.78E-12	1.91E-08	+1	89.60	29.30	25.38	24.89	26.06	20.13	28.96	23.94	25.53	19.62	23.46	93.00	25.97	18.94	33.25	25.20	22.44	27.10	90.97	92.29	37.36	26.64	29.46	90.77	28.32	92.34	92.89	89.63	23.65	88.85	30.66	91.02
NRXN3	chr14.0080170888	0.930	1.91E-12	2.03E-08	+1	26.79	20.30	19.54	24.65	22.02	20.95	19.14	18.26	20.51	23.23	19.42	23.29	17.06	13.11	15.39	21.78	18.41	96.07	14.00	18.98	19.17	23.52	18.66	19.48	16.09	25.11	95.01	93.96	94.71	14.98	20.83	33.62
EXOC3L4	chr14.0103575195	0.823	1.96E-12	2.07E-08	+1	42.16	41.98	36.16	35.49	91.35	16.89	34.87	35.52	25.23	32.70	30.43	38.00	24.62	21.76	35.91	28.65	91.43	25.59	36.39	34.70	38.44	92.86	94.40	92.90	91.03	31.25	40.50	39.51	91.43	93.05	27.36	93.35
NA	chr12.0125198038	-0.905	2.06E-12	2.16E-08	-1	14.79	97.85	13.41	21.78	96.78	92.85	19.83	11.18	96.19	15.78	18.94	19.72	22.08	96.66	22.29	21.70	18.80	21.58	96.74	10.20	21.48	17.28	14.93	27.98	15.16	17.94	16.68	14.59	14.70	20.88	15.25	19.63
MYO1C	chr17.0001390159	-0.848	2.26E-12	2.35E-08	-1	34.56	25.57	24.90	22.55	26.81	21.98	95.17	93.45	21.08	92.60	25.07	92.48	93.74	91.99	24.49	88.96	25.61	23.40	39.44	21.15	24.98	24.27	17.37	24.51	24.85	25.74	93.42	26.17	25.35	30.45	27.21	32.34
NA	chr14.0104820706	0.804	2.28E-12	2.36E-08	+1	40.39	35.63	70.25	40.57	35.77	33.89	32.48	67.33	28.23	34.30	47.96	41.10	38.52	29.46	32.73	31.59	70.18	94.30	36.81	90.99	94.40	91.20	29.48	31.03	92.46	39.09	93.59	70.86	68.94	88.70	36.55	91.78
TAPBP	chr6.0033280212	-0.768	2.43E-12	2.50E-08	-1	92.04	85.38	96.52	96.77	91.75	90.17	90.41	26.49	97.15	93.08	95.14	94.84	91.42	93.56	94.15	95.43	20.11	96.61	29.22	33.77	36.94	85.86	15.51	91.66	95.70	96.76	26.87	95.25	29.80	22.22	29.43	30.64
NA	chr13.0021100521	0.935	2.45E-12	2.50E-08	+1	27.41	32.66	15.62	19.42	22.11	21.30	16.37	17.68	13.08	20.83	17.92	13.91	18.27	16.49	16.93	22.16	18.69	14.73	21.97	19.14	92.34	12.41	93.55	21.76	96.28	17.08	24.25	22.75	14.66	17.62	17.84	94.23
FRMD6	chr14.0052119953	-0.997	2.47E-12	2.51E-08	-1	21.36	17.30	93.19	20.00	14.55	26.68	31.75	10.92	7.51	14.81	93.31	17.10	9.05	92.59	9.15	7.48	17.10	15.19	14.67	13.38	11.51	20.36	9.54	25.10	17.24	13.56	16.49	9.07	16.85	14.98	11.42	16.85
OSBPL10	chr3.0032022182	-0.820	2.57E-12	2.59E-08	-1	90.33	29.30	22.65	91.54	12.75	91.55	92.27	92.46	22.17	93.06	32.03	92.95	90.41	89.40	27.81	34.45	20.66	20.49	27.77	90.92	20.88	94.60	23.75	23.74	92.43	16.59	16.65	24.23	30.23	16.53	26.68	23.09
EBF2	chr8.0025900066	-0.889	2.62E-12	2.62E-08	-1	90.07	88.22	21.84	29.04	25.39	19.18	88.34	24.25	90.37	24.98	29.73	24.43	91.11	20.71	21.74	22.92	20.26	27.80	22.71	24.57	17.01	26.29	26.61	20.21	22.81	14.00	23.37	24.61	29.06	28.46	16.47	39.66
NA	chr3.0125094759	-0.773	2.80E-12	2.78E-08	-1	91.62	94.17	93.62	92.84	96.42	92.07	24.66	92.97	96.47	93.35	92.28	93.86	93.22	14.51	94.53	94.86	93.57	93.70	17.17	23.73	93.09	25.94	21.36	14.30	31.61	30.12	21.44	94.73	21.11	92.82	25.74	90.17
NA	chr3.0101232351	0.837	2.88E-12	2.84E-08	+1	92.64	17.19	20.42	21.54	17.89	93.69	15.39	94.56	19.90	19.74	16.51	19.44	15.72	12.92	16.09	12.82	19.10	93.70	96.28	23.18	17.53	94.28	96.48	95.05	16.39	21.49	10.79	22.03	95.78	18.17	92.34	94.66
NA	chr19.0011397313	-0.867	2.90E-12	2.85E-08	-1	38.88	35.61	28.34	89.02	29.04	27.14	93.12	18.91	91.77	91.45	24.67	28.53	90.44	35.16	25.75	30.66	25.26	30.01	31.28	22.98	20.93	25.84	24.34	23.32	24.07	24.44	30.71	31.88	22.38	26.19	27.18	35.41
CCBE1	chr18.0057357804	-0.869	2.92E-12	2.85E-08	-1	87.65	24.65	14.05	92.13	93.64	26.96	25.52	14.15	91.12	24.78	23.24	26.10	92.39	23.82	22.54	89.92	17.94	22.94	20.77	13.57	28.64	21.02	23.04	30.57	16.97	22.04	13.03	19.92	20.14	28.83	30.77	92.55
RNF114	chr20.0048552544	-0.864	3.27E-12	3.17E-08	-1	25.39	91.68	12.58	24.33	23.03	96.59	96.46	15.00	17.15	96.34	98.99	18.58	96.31	27.52	15.47	26.12	13.16	95.57	21.69	15.48	13.81	13.91	18.05	18.59	22.56	18.03	16.24	14.81	21.23	97.94	13.98	13.82
TSPAN9	chr12.0003241735	0.791	3.35E-12	3.22E-08	+1	92.04	38.56	32.47	39.76	39.02	42.42	36.81	37.56	34.23	42.94	37.13	40.88	35.80	39.95	38.77	43.86	32.95	93.03	50.60	92.43	35.47	89.90	44.59	91.67	88.43	93.17	92.50	41.05	40.39	93.08	91.84	94.95
NA	chr2.0071123967	-0.899	3.49E-12	3.33E-08	-1	23.23	18.60	68.25	28.29	30.99	38.76	19.67	14.25	69.81	69.86	34.76	36.34	68.08	94.68	22.20	30.68	22.06	16.78	17.10	28.36	40.36	20.44	28.53	19.91	17.43	14.26	26.49	15.58	18.85	19.81	16.40	35.57
CLDN10	chr13.0096206439	-0.778	3.51E-12	3.33E-08	-1	93.74	97.72	98.39	95.38	94.34	94.52	3.60	94.25	98.03	98.19	95.68	4.31	9.34	98.00	96.58	94.70	98.47	16.05	2.47	97.13	98.11	2.90	2.19	8.33	10.39	94.60	9.62	3.35	100.05	97.45	2.40	94.90
HOXB-AS3	chr17.0046675549	-0.902	3.98E-12	3.76E-08	-1	15.64	18.85	14.92	15.76	15.47	17.80	16.65	20.28	93.13	13.67	93.79	94.35	94.18	94.18	15.94	18.97	19.66	14.42	19.78	10.87	19.08	15.36	14.00	19.79	16.72	16.03	15.27	18.81	12.67	25.44	93.48	17.83
NA	chr12.0033201624	-0.770	4.07E-12	3.82E-08	-1	92.04	47.49	93.55	44.22	93.41	93.46	92.90	90.80	44.83	93.15	87.73	75.03	92.59	92.49	94.64	91.89	92.25	35.97	75.61	40.15	46.35	24.75	32.47	46.36	38.26	51.73	22.06	41.08	90.97	39.02	50.63	46.49
AK127919	chr17.0078944136	-0.876	4.26E-12	3.98E-08	-1	36.74	30.50	23.03	21.51	20.31	22.79	24.24	31.04	89.52	12.34	89.17	88.35	30.98	89.48	24.86	88.54	24.82	19.96	38.98	26.07	25.15	19.14	20.58	27.59	18.73	21.89	27.28	26.98	20.33	29.55	21.63	24.98
X69637	chr16.0032773264	0.818	4.58E-12	4.25E-08	+1	16.18	32.03	90.10	14.35	23.18	25.66	87.58	20.95	22.89	11.61	17.98	20.82	34.26	19.40	21.25	85.78	17.03	89.31	22.07	21.85	30.14	88.63	91.01	88.86	21.63	91.65	20.49	88.69	90.56	89.79	89.77	19.41
EPM2A	chr6.0145853785	-0.824	4.62E-12	4.26E-08	-1	87.22	12.93	20.87	30.65	28.55	91.31	93.65	25.08	26.85	29.78	19.53	90.99	90.14	93.37	92.09	91.83	25.48	24.06	17.44	24.01	28.87	22.85	23.24	28.86	18.21	32.16	26.56	92.51	93.69	23.33	20.71	20.29
NA	chr8.0041168893	0.819	4.75E-12	4.36E-08	+1	31.58	33.62	29.51	88.85	30.09	22.06	26.73	22.53	30.66	30.57	33.49	32.01	33.59	28.74	28.99	35.37	30.65	89.99	31.80	25.61	88.02	88.56	27.23	33.77	34.75	27.58	88.14	88.00	34.07	88.38	88.39	87.23
NA	chr11.0130304917	-0.808	4.83E-12	4.40E-08	-1	92.42	91.64	93.16	33.13	92.58	20.79	27.20	94.19	94.55	22.01	92.84	22.53	90.90	22.64	26.80	91.27	17.59	24.14	21.13	22.30	92.00	27.01	92.26	23.45	28.31	24.57	17.12	26.12	27.15	14.58	28.16	89.81
NA	chr2.0216785021	0.834	4.94E-12	4.48E-08	+1	25.74	30.11	26.36	23.63	29.34	25.68	28.08	20.00	27.80	22.70	24.53	24.00	94.11	29.87	30.55	26.22	94.69	92.19	93.35	28.02	23.69	93.15	26.98	20.45	93.52	23.25	23.36	92.84	26.53	24.82	92.42	24.20
PIEZO2	chr18.0010938440	0.949	5.03E-12	4.52E-08	+1	18.03	14.21	9.42	9.01	11.56	14.95	16.94	11.94	12.82	96.09	8.63	12.35	13.74	13.56	7.63	8.36	11.62	8.93	17.58	95.29	14.05	13.24	96.59	16.25	95.94	11.15	5.67	96.06	13.80	26.51	11.12	7.22
NA	chr13.0068862091	0.820	5.05E-12	4.52E-08	+1	21.74	24.18	96.72	15.93	30.31	30.42	22.12	21.34	93.05	29.21	28.98	26.93	19.10	26.08	21.25	24.63	21.12	93.99	93.14	26.28	92.90	23.04	24.81	25.86	95.62	20.09	26.06	92.06	94.72	92.67	93.27	25.30
NA	chr15.0041695223	-0.847	5.11E-12	4.54E-08	-1	91.49	36.59	21.65	17.03	18.78	93.73	91.62	26.52	92.61	28.10	29.14	94.24	34.70	25.48	24.68	89.59	21.73	25.55	23.19	22.15	91.38	22.07	29.78	23.10	28.87	22.61	25.03	19.33	20.91	22.86	23.58	31.01
NA	chr3.0167100340	0.759	5.31E-12	4.69E-08	+1	87.78	93.27	18.52	24.89	23.61	89.46	92.27	93.42	19.64	94.99	22.72	19.79	26.60	18.08	22.26	74.63	72.42	93.33	93.47	91.72	91.54	93.75	93.45	90.39	92.78	90.71	90.79	93.25	93.89	89.48	29.44	92.93
NA	chr18.0071356718	0.779	5.48E-12	4.80E-08	+1	88.58	30.51	36.02	22.58	24.65	19.53	25.22	33.84	89.50	90.81	30.68	87.93	34.06	28.25	22.20	29.43	91.87	26.49	86.70	26.80	89.72	23.50	92.58	93.52	93.61	91.23	18.84	90.58	91.85	90.14	92.13	93.42
MTRF1	chr13.0041796041	-0.775	5.48E-12	4.80E-08	-1	88.02	22.46	27.94	93.07	94.09	93.19	93.19	92.19	92.81	47.78	91.11	91.50	94.95	92.97	21.41	94.11	92.95	21.73	29.92	94.41	93.15	23.75	18.82	18.87	30.98	93.40	22.31	20.80	23.07	25.34	93.27	16.22
NA	chr6.0136611329	-0.813	6.06E-12	5.27E-08	-1	89.60	90.84	30.14	37.64	92.61	42.57	89.23	24.84	92.04	90.71	38.39	49.11	27.86	31.45	89.54	42.28	28.18	30.93	90.55	30.06	34.72	28.75	28.62	51.14	24.32	23.72	28.28	30.51	27.65	31.56	22.48	33.94
TYW5	chr2.0200819443	-0.802	6.42E-12	5.54E-08	-1	94.26	95.90	93.73	23.92	20.97	94.28	93.54	93.86	24.45	93.58	93.34	25.23	28.17	93.65	22.08	22.86	23.63	21.24	23.77	20.17	25.22	24.18	27.62	24.62	23.13	16.42	94.89	93.95	16.96	21.75	93.80	30.02
RCN1	chr11.0032113404	-0.860	6.44E-12	5.54E-08	-1	27.15	13.73	96.35	22.76	96.08	95.66	13.75	14.50	9.40	15.98	17.73	95.55	92.96	95.19	19.26	25.35	13.60	21.40	19.70	12.64	14.30	17.98	14.36	96.89	15.05	7.96	18.76	13.39	16.48	14.58	22.32	94.67
DCC	chr18.0050319582	0.777	6.51E-12	5.56E-08	+1	38.06	71.28	40.74	37.15	39.61	37.54	45.88	44.56	44.04	44.28	33.20	33.44	37.30	40.43	73.89	31.66	71.68	39.56	94.07	48.26	92.03	69.58	93.28	39.45	92.59	38.70	94.75	48.33	92.70	90.99	90.61	93.42
ZNF787	chr19.0056616939	0.785	6.73E-12	5.72E-08	+1	38.58	53.90	43.14	34.13	37.34	36.37	34.02	29.91	41.81	36.59	37.34	38.84	37.26	32.94	31.44	88.23	87.23	42.40	47.23	40.07	86.09	89.43	88.90	89.02	89.29	90.86	87.53	88.68	27.49	38.66	90.75	41.71
LINC00237	chr20.0021083144	-1.033	7.20E-12	6.09E-08	-1	14.00	18.67	14.00	16.42	17.75	14.03	20.74	97.90	12.37	98.20	13.46	15.58	14.35	6.87	17.78	16.16	12.33	16.04	16.62	10.66	14.01	10.04	9.79	7.39	15.21	12.88	11.94	11.15	14.89	16.39	5.55	13.82
RPS6KL1	chr14.0075389585	0.791	7.69E-12	6.47E-08	+1	34.54	27.97	37.84	40.23	31.53	40.84	38.16	38.09	24.13	36.36	30.72	38.24	86.71	37.38	35.84	36.41	45.24	34.95	87.44	86.56	88.78	88.99	43.96	86.24	41.41	38.41	41.77	33.88	88.27	88.70	87.70	84.15
NTNG2	chr9.0135118027	0.751	7.77E-12	6.50E-08	+1	38.01	32.79	36.41	91.62	28.05	92.22	31.17	42.05	91.94	24.60	92.72	37.87	30.62	90.58	36.97	36.12	92.46	90.46	89.40	93.46	90.73	91.64	93.42	90.75	93.05	33.20	92.11	90.50	92.30	88.14	93.20	92.00
WWOX	chr16.0078675586	0.796	8.18E-12	6.78E-08	+1	25.44	23.01	87.11	26.45	24.44	20.38	31.28	88.23	29.40	27.54	16.22	24.95	89.63	25.52	25.88	22.62	20.65	22.01	88.29	88.36	87.36	87.50	18.58	88.34	88.45	24.67	28.06	87.62	86.20	89.06	87.33	31.22
NA	chr14.0102030873	-0.871	8.19E-12	6.78E-08	-1	92.70	19.25	92.73	26.01	18.44	25.86	23.16	92.70	12.92	19.94	94.46	26.80	15.16	19.12	28.73	91.49	15.75	20.44	15.44	17.59	20.58	23.30	13.18	19.48	13.94	17.98	23.79	20.39	22.30	27.34	18.43	92.10
ZBTB7C	chr18.0045934806	-0.892	8.30E-12	6.83E-08	-1	22.51	14.75	25.08	19.21	94.78	17.30	91.44	23.10	24.30	27.85	35.93	34.16	22.14	28.11	94.28	66.36	29.84	17.08	19.41	23.25	18.98	18.96	12.30	39.20	21.35	13.12	10.49	24.99	25.04	17.93	21.95	31.15
NA	chr11.0001846450	-0.755	8.63E-12	7.06E-08	-1	88.37	87.89	93.70	93.15	93.26	94.85	24.71	92.47	93.36	29.19	91.68	94.88	89.94	92.46	93.39	94.60	31.09	25.44	31.04	30.71	29.63	29.72	94.06	20.54	27.20	33.74	92.76	90.73	42.92	91.32	95.63	32.52
N4BP1	chr16.0048639869	-0.821	9.07E-12	7.39E-08	-1	34.62	30.79	31.74	85.93	86.58	21.40	31.90	32.93	26.11	89.41	26.26	34.15	88.09	88.98	87.58	84.47	29.97	24.96	87.70	23.23	21.47	20.66	23.44	32.03	25.79	28.64	29.30	28.72	29.89	28.78	29.52	34.40
NA	chr22.0050166610	-0.869	9.39E-12	7.60E-08	-1	26.92	93.10	23.83	20.80	14.89	25.95	92.47	19.88	16.78	20.94	92.51	19.33	22.31	26.91	89.52	92.49	32.15	23.34	31.06	26.76	28.23	21.51	31.42	18.37	20.82	13.02	20.54	26.86	28.38	16.12	17.84	25.55

Gene Annotation	Chromosome Position	Log Fold Change	p-value	p(FDR)	Methylation Up/Down in CP	Control																CP															
						xx039	x053	x056	x057	x370	x380	x414	x416	x425	x431	x436	x466	x489	x527	x563	x584	x010	x028	x045	x050	x375	x376	x417	x440	x444	x533	x568	x573	x583	x597	x600	x606
H2BFB	chr6.0026159113	1.024	9.98E-12	8.04E-08	+1	17.32	10.40	4.05	5.63	14.85	12.69	12.72	18.07	16.83	12.31	12.10	16.34	9.43	7.21	11.69	18.37	9.22	10.92	23.87	4.21	25.21	20.18	17.97	16.37	12.23	21.77	91.19	5.90	21.97	93.35	6.49	28.61
NA	chr13.0095359203	-0.833	1.07E-11	8.59E-08	-1	88.52	26.15	24.90	93.92	28.70	21.10	21.02	25.86	36.20	26.33	90.24	25.33	92.81	25.74	94.99	94.11	25.93	33.66	30.96	30.78	30.89	25.15	20.61	24.23	33.50	23.20	28.34	27.72	23.88	32.18	33.23	33.56
SRXN1	chr20.0000633169	-0.826	1.08E-11	8.59E-08	-1	90.18	25.14	90.66	27.98	89.11	29.36	94.87	93.17	25.67	18.47	39.99	38.54	34.22	20.85	93.07	31.31	22.53	29.34	20.64	92.97	25.48	21.41	30.27	26.73	27.05	28.73	27.38	10.32	24.69	29.84	29.69	27.55
NA	chr20.0025371911	-0.809	1.10E-11	8.69E-08	-1	18.44	18.67	24.29	96.47	95.89	96.03	19.99	21.22	16.10	95.33	96.04	95.65	18.91	94.67	18.89	94.56	18.68	16.04	17.82	21.77	20.67	94.05	13.21	18.55	18.81	20.75	20.71	91.87	24.33	15.71	94.38	18.27
NA	chr11.0020364247	-0.802	1.13E-11	8.94E-08	-1	30.13	91.38	19.87	27.47	93.85	22.92	20.71	94.93	94.72	20.44	93.76	92.34	90.90	29.51	95.09	36.72	23.76	27.85	93.65	94.70	28.90	19.28	19.26	29.10	25.31	23.64	33.80	20.77	24.43	22.95	24.84	34.90
NPR3	chr5.0032712315	-0.800	1.27E-11	9.95E-08	-1	94.87	93.49	16.85	16.94	95.02	93.47	13.30	19.41	11.01	12.05	93.80	94.32	95.79	19.63	92.65	94.02	15.22	94.82	8.87	16.78	12.52	11.68	18.78	95.04	8.61	10.11	21.30	15.46	96.53	13.94	95.06	14.56
SLC24A3	chr20.0019192507	0.837	1.38E-11	1.08E-07	+1	27.14	18.67	6.48	19.64	12.66	10.43	17.07	96.02	16.10	14.73	13.90	96.57	22.23	20.34	14.29	28.30	9.89	42.82	96.10	24.87	12.82	17.68	96.74	95.52	15.04	97.46	23.71	95.97	17.60	16.39	20.09	96.54
NA	chr17.0018992459	0.826	1.44E-11	1.12E-07	+1	91.17	24.59	16.56	14.85	21.46	20.80	17.68	17.90	20.38	19.81	16.26	94.01	19.63	27.63	23.79	14.32	18.64	18.14	94.18	91.58	14.44	95.12	9.07	10.33	97.11	23.89	22.12	21.68	94.47	93.07	24.03	91.64
STAB2	chr12.0103980970	0.821	1.45E-11	1.12E-07	+1	27.11	42.47	94.43	25.97	23.60	21.00	25.14	26.90	22.84	29.83	22.43	23.13	24.41	20.00	25.69	20.69	91.81	94.95	27.63	93.58	24.00	95.33	22.88	24.49	29.62	34.40	28.64	94.43	24.87	26.25	94.57	34.41
HHATL	chr3.0042740649	-0.829	1.55E-11	1.19E-07	-1	92.64	19.05	23.46	92.02	25.26	24.75	95.16	20.56	20.16	24.19	92.63	28.65	30.04	92.59	21.95	97.25	17.69	19.64	17.44	93.34	11.59	94.75	14.81	18.83	25.12	14.62	14.68	22.73	19.89	17.34	21.01	26.51
NA	chr20.0055582211	-0.762	1.60E-11	1.22E-07	-1	28.75	94.07	94.51	95.78	93.55	95.62	29.28	27.13	27.43	95.86	32.59	92.99	92.55	93.98	93.46	92.65	28.04	27.16	37.91	26.59	28.78	22.84	93.23	32.61	29.70	27.02	31.39	93.94	40.82	28.86	93.61	53.17
GAS7	chr17.0009939872	-0.750	1.65E-11	1.27E-07	-1	92.62	94.35	94.86	91.58	92.88	92.07	18.96	90.59	93.60	94.34	93.96	91.70	25.27	36.59	88.92	90.87	93.25	92.07	29.81	28.18	91.10	23.76	26.34	25.98	21.64	25.14	25.50	92.31	94.83	31.17	23.16	37.09
ANKARD13A	chr12.0110035773	0.829	1.73E-11	1.33E-07	+1	25.06	30.86	21.13	28.37	31.97	25.01	34.06	32.82	20.04	34.30	25.85	38.15	19.76	29.99	21.27	26.37	83.36	25.64	24.35	32.86	43.00	29.16	27.22	42.62	25.96	27.51	91.82	91.05	21.97	34.72	95.29	95.00

Table S2. Top 200 Differentially Methylated Genes.

Genes Hypermethylated in Control Subjects

		Gene Ontologies						
UniProtID_Link	Protein Name	Function	Component	Process	KEGG Pathway	HUGO Gene Name	OMIM ID	Methylation Score
http://www.uniprot.org/uniprot/Q8NH85	Olfactory receptor 5R1	0038023 : signaling receptor activity; 0005549 : odorant binding	0016020 : membrane	0007154 : cell communication; 0050789 : regulation of biological process; 0050896 : response to stimulus	Olfactory transduction [PATH:hsa04740]219479	OR5R1		1736
http://www.uniprot.org/uniprot/Q8NGC5	Olfactory receptor 6J1	0038023 : signaling receptor activity	0016020 : membrane			OR6J1		1270.2
http://www.uniprot.org/uniprot/Q8NGN2	Olfactory receptor 10S1	0038023 : signaling receptor activity	0016020 : membrane	0007154 : cell communication; 0050789 : regulation of biological process; 0050896 : response to stimulus; 0050877 : nervous system process	Olfactory transduction [PATH:hsa04740]219873	OR10S1		1083.1
http://www.uniprot.org/uniprot/Q9UNA3	Alpha-1,4-N-acetylglucosaminyltransferase	0016740 : transferase activity	0005794 : Golgi apparatus; 0016020 : membrane	0005975 : carbohydrate metabolic process; 0009059 : macromolecule biosynthetic process; 0019538 : protein metabolic process; 0008283 : cell proliferation; 0050789 : regulation of biological process; 0006464 : cellular protein modification process		A4GNT		1064.8
http://www.uniprot.org/uniprot/A6NHA9	Olfactory receptor 4C46	0038023 : signaling receptor activity	0016020 : membrane	0050877 : nervous system process; 0051606 : detection of stimulus; 0007154 : cell communication; 0050789 : regulation of biological process; 0050896 : response to stimulus	Olfactory transduction [PATH:hsa04740]119749	OR4C46		1021.5
http://www.uniprot.org/uniprot/Q8NGE9	Olfactory receptor 9Q2	0038023 : signaling receptor activity; 0005549 : odorant binding	0016020 : membrane	0007154 : cell communication; 0050789 : regulation of biological process; 0050896 : response to stimulus	Olfactory transduction [PATH:hsa04740]219957	OR9Q2		872.4
http://www.uniprot.org/uniprot/Q9Y4X3	C-C motif chemokine 27 precursor	0005515 : protein binding	0005576 : extracellular region	0002376 : immune system process; 0050896 : response to stimulus; 0051704 : multi-organism process; 0007154 : cell communication; 0040011 : locomotion; 0050789 : regulation of biological process; 0031640 : killing of cells of other organism	Cytokine-cytokine receptor interaction [PATH:hsa04060]10850. Chemokine signaling pathway [PATH:hsa04062]10850. Intestinal immune network for IgA production [PATH:hsa04672]10850	CCL27	604833	810.5
http://www.uniprot.org/uniprot/Q9Y4A9	Olfactory receptor 10H1	0038023 : signaling receptor activity	0016020 : membrane		Olfactory transduction [PATH:hsa04740]26539	OR10H1		775.4
http://www.uniprot.org/uniprot/Q9NS67	Probable G-protein coupled receptor 27	0038023 : signaling receptor activity	0016020 : membrane	0007154 : cell communication; 0050789 : regulation of biological process; 0050896 : response to stimulus; 0009914 : hormone transport; 0015031 : protein transport; 0046903 : secretion; 0051641 : cellular localization		GPR27	605187	748.8
http://www.uniprot.org/uniprot/Q8NH60	Olfactory receptor 52J3	0038023 : signaling receptor activity	0016020 : membrane	0007154 : cell communication; 0050789 : regulation of biological process; 0050896 : response to stimulus	Olfactory transduction [PATH:hsa04740]119679	OR52J3		722.9
http://www.uniprot.org/uniprot/Q8TDV5	Glucose-dependent insulinotropic receptor	0038023 : signaling receptor activity; 0008289 : lipid binding; 0043167 : ion binding	0016020 : membrane; 0043235 : receptor complex	0007154 : cell communication; 0009914 : hormone transport; 0015031 : protein transport; 0046903 : secretion		GPR119	300513	693.4
http://www.uniprot.org/uniprot/Q8NGL7	Olfactory receptor 4P4	0038023 : signaling receptor activity	0016020 : membrane	0050877 : nervous system process; 0051606 : detection of stimulus; 0007154 : cell communication; 0050789 : regulation of biological process; 0050896 : response to stimulus	Olfactory transduction [PATH:hsa04740]81300	OR4P4		683.8
http://www.uniprot.org/uniprot/Q9BYW3	Beta-defensin 126 precursor		0009986 : cell surface; 0005576 : extracellular region	0002376 : immune system process; 0050896 : response to stimulus; 0051704 : multi-organism process; 0000003 : reproduction		DEFB126		660.4
http://www.uniprot.org/uniprot/Q9NV35	Nucleotide triphosphate diphosphatase NUDT15	0016787 : hydrolase activity; 0043167 : ion binding	0005737 : cytoplasm	0009117 : nucleotide metabolic process; 0006259 : DNA metabolic process; 0007154 : cell communication; 0050896 : response to stimulus; 0017144 : drug metabolic process; 0007049 : cell cycle; 0006139 : nucleobase-containing compound metabolic process; 0006793 : phosphorus metabolic process; 0006508 : proteolysis; 0009057 : macromolecule catabolic process; 0050789 : regulation of biological process		NUDT15	615792/	646.4

http://www.uniprot.org/uniprot/Q96RI9	Trace amine-associated receptor 9	0038023 : signaling receptor activity	0016020 : membrane	0007154 : cell communication; 0050789 : regulation of biological process; 0050896 : response to stimulus	Neuroactive ligand-receptor interaction [PATH:hsa04080]134860	TAAR9	608282	602.1
http://www.uniprot.org/uniprot/A6NJZ3	Olfactory receptor 6C65	0038023 : signaling receptor activity	0016020 : membrane		Olfactory transduction [PATH:hsa04740]403282	OR6C65		559.8
http://www.uniprot.org/uniprot/Q8IUE6	Histone H2A type 2-B	0003676 : nucleic acid binding; 0005515 : protein binding	0031982 : vesicle; 0043230 : extracellular organelle; 0005634 : nucleus; 0005694 : chromosome; 0000786 : nucleosome	0006351 : transcription, DNA-templated; 0016458 : gene silencing; 0051276 : chromosome organization	Systemic lupus erythematosus [PATH:hsa05322]317772	HIST2H2AB	615014	548.5
http://www.uniprot.org/uniprot/Q9NP92	39S ribosomal protein S30, mitochondrial	0003676 : nucleic acid binding; 0005198 : structural molecule activity	0005739 : mitochondrion; 0016020 : membrane; 0005840 : ribosome	0008150 : biological_process; 0006412 : translation; 0022411 : cellular component disassembly		MRPS30	611991	497.4
http://www.uniprot.org/uniprot/Q8NH72	Olfactory receptor 4C6	0038023 : signaling receptor activity	0016020 : membrane	0050877 : nervous system process; 0051606 : detection of stimulus; 0007154 : cell communication; 0050789 : regulation of biological process; 0050896 : response to stimulus	Olfactory transduction [PATH:hsa04740]219432	OR4C6		457.8
http://www.uniprot.org/uniprot/Q8NH81	Olfactory receptor 10G6	0038023 : signaling receptor activity	0016020 : membrane	0007154 : cell communication; 0050789 : regulation of biological process; 0050896 : response to stimulus; 0050877 : nervous system process		OR10G6		446.3
http://www.uniprot.org/uniprot/Q9BWM5	Zinc finger protein 416	0003676 : nucleic acid binding; 0043167 : ion binding	0005634 : nucleus	0006351 : transcription, DNA-templated; 0050789 : regulation of biological process		ZNF416		444.3
http://www.uniprot.org/uniprot/Q96RB7	Olfactory receptor 5M11	0038023 : signaling receptor activity; 0005549 : odorant binding	0016020 : membrane	0007154 : cell communication; 0050789 : regulation of biological process; 0050896 : response to stimulus	Olfactory transduction [PATH:hsa04740]219487	OR5M11		440.1
http://www.uniprot.org/uniprot/Q9BV90	U11/U12 small nuclear ribonucleoprotein 25 kDa protein		0005737 : cytoplasm; 0005576 : extracellular region; 0005634 : nucleus; 0005681 : spliceosomal complex	0000375 : RNA splicing, via transesterification reactions; 0006396 : RNA processing		SNRNP25		430.3
http://www.uniprot.org/uniprot/Q8NGE5	Olfactory receptor 10A7	0038023 : signaling receptor activity	0016020 : membrane	0007154 : cell communication; 0050789 : regulation of biological process; 0050896 : response to stimulus; 0050877 : nervous system process	Olfactory transduction [PATH:hsa04740]121364	OR10A7		409.2
http://www.uniprot.org/uniprot/Q8NG97	Olfactory receptor 2Z1	0038023 : signaling receptor activity	0016020 : membrane	0007154 : cell communication; 0050789 : regulation of biological process; 0050896 : response to stimulus; 0050877 : nervous system process	Olfactory transduction [PATH:hsa04740]284383	OR2Z1		376.8
http://www.uniprot.org/uniprot/Q96L33	Rho-related GTP-binding protein RhoV	0000166 : nucleotide binding; 0001882 : nucleoside binding; 0043167 : ion binding; 0016787 : hydrolase activity	0005737 : cytoplasm; 0005768 : endosome; 0016020 : membrane	0007154 : cell communication; 0050789 : regulation of biological process; 0050896 : response to stimulus		RHOV		375.3
http://www.uniprot.org/uniprot/Q8IXM3	39S ribosomal protein L41, mitochondrial precursor	0003676 : nucleic acid binding; 0005198 : structural molecule activity	0005739 : mitochondrion; 0016020 : membrane; 0005840 : ribosome	0008150 : biological_process; 0007049 : cell cycle; 0006412 : translation; 0022411 : cellular component disassembly		MRPL41	611846	369.9
http://www.uniprot.org/uniprot/Q8NGP6	Olfactory receptor 5M8	0038023 : signaling receptor activity; 0005549 : odorant binding	0016020 : membrane	0007154 : cell communication; 0050789 : regulation of biological process; 0050896 : response to stimulus	Olfactory transduction [PATH:hsa04740]219484	OR5M8		369.6
http://www.uniprot.org/uniprot/Q9UBV4	Protein Wnt-16 precursor	0005515 : protein binding	0005737 : cytoplasm; 0005576 : extracellular region; 0031012 : extracellular matrix	0048771 : tissue remodeling; 0032501 : multicellular organismal process; 0032502 : developmental process; 0008283 : cell proliferation; 0050789 : regulation of biological process; 0050896 : response to stimulus; 0043170 : macromolecule metabolic process; 0006464 : cellular protein modification process; 0006793 : phosphorus metabolic process; 0007154 : cell communication	Wnt signaling pathway [PATH:hsa04310]51384. Hedgehog signaling pathway [PATH:hsa04340]51384. Melanogenesis [PATH:hsa04916]51384. Pathways in cancer [PATH:hsa05200]51384. Basal cell carcinoma [PATH:hsa05217]51384	WNT16	606267	368.9

http://www.uniprot.org/uniprot/Q8TAT2	Fibroblast growth factor-binding protein 3 precursor	0005515 : protein binding; 0043167 : ion binding	0031012 : extracellular matrix; 0005576 : extracellular region	0007154 : cell communication; 0050789 : regulation of biological process; 0050896 : response to stimulus; 0008015 : blood circulation; 0065008 : regulation of biological quality		FGFBP3		365.3
http://www.uniprot.org/uniprot/Q9GZZ0	Homeobox protein Hox-D1	0003674 : molecular_function; 0003676 : nucleic acid binding	0005634 : nucleus	0032501 : multicellular organismal process; 0032502 : developmental process; 0050877 : nervous system process; 0006351 : transcription, DNA-templated		HOXD1	142987	363.6
http://www.uniprot.org/uniprot/Q8NBF2	NHL repeat-containing protein 2		0005576 : extracellular region; 0005737 : cytoplasm; 0031982 : vesicle; 0043229 : intracellular organelle	0050789 : regulation of biological process; 0065008 : regulation of biological quality; 0016192 : vesicle-mediated transport; 0046903 : secretion		NHLRC2		358.3
http://www.uniprot.org/uniprot/Q9H3M0	Potassium voltage-gated channel subfamily F member 1	0005215 : transporter activity	0016020 : membrane; 0032991 : protein-containing complex	0006811 : ion transport; 0065003 : protein-containing complex assembly; 0050789 : regulation of biological process		KCNF1	603787	337.5
http://www.uniprot.org/uniprot/Q8NGA6	Olfactory receptor 10H5	0038023 : signaling receptor activity	0016020 : membrane		Olfactory transduction [PATH:hsa04740]284433	OR10H5		335.5
http://www.uniprot.org/uniprot/Q8NGB4	Olfactory receptor 4S1	0038023 : signaling receptor activity	0016020 : membrane	0050877 : nervous system process; 0051606 : detection of stimulus; 0007154 : cell communication; 0050789 : regulation of biological process; 0050896 : response to stimulus	Olfactory transduction [PATH:hsa04740]256148	OR4S1		332.5
http://www.uniprot.org/uniprot/Q9UN37	Vacuolar protein sorting-associated protein 4A	0000166 : nucleotide binding; 0008144 : drug binding; 0043167 : ion binding; 0016787 : hydrolase activity; 0005515 : protein binding	0005813 : centrosome; 0005737 : cytoplasm; 0005768 : endosome; 0031982 : vesicle; 0043230 : extracellular organelle; 0030496 : midbody; 0016020 : membrane; 0005773 : vacuole; 0005634 : nucleus; 0005856 : cytoskeleton	0032501 : multicellular organismal process; 0032502 : developmental process; 0006996 : organelle organization; 0007049 : cell cycle; 0051301 : cell division; 0016192 : vesicle-mediated transport; 0051641 : cellular localization; 0016050 : vesicle organization; 0048284 : organelle fusion; 0061024 : membrane organization; 0022411 : cellular component disassembly; 0006869 : lipid transport; 0006914 : autophagy; 0050789 : regulation of biological process; 0000280 : nuclear division; 0007059 : chromosome segregation; 0051234 : establishment of localization; 0051276 : chromosome organization; 0051640 : organelle localization; 0007032 : endosome organization; 0022607 : cellular component assembly; 0006997 : nucleus organization; 0044085 : cellular component biogenesis; 0046903 : secretion; 0016032 : viral process; 0040011 : locomotion; 0015031 : protein transport; 0051179 : localization; 0006508 : proteolysis; 0009057 : macromolecule catabolic process; 0051261 : protein depolymerization	Endocytosis [PATH:hsa04144]27183	VPS4A	609982	331.6
http://www.uniprot.org/uniprot/Q3B726	DNA-directed RNA polymerase I subunit RPA43	0016740 : transferase activity	0005730 : nucleolus; 0030880 : RNA polymerase complex; 0005634 : nucleus	0050896 : response to stimulus; 0043170 : macromolecule metabolic process; 0050789 : regulation of biological process; 0006351 : transcription, DNA-templated; 0006354 : DNA-templated transcription, elongation		TWISTNB	608312	319.3
http://www.uniprot.org/uniprot/Q9H598	Vesicular inhibitory amino acid transporter	0005215 : transporter activity	0044464 : cell part; 0005737 : cytoplasm; 0016020 : membrane; 0031982 : vesicle; 0043229 : intracellular organelle; 0043005 : neuron projection; 0045202 : synapse	0032502 : developmental process; 0006865 : amino acid transport; 0032501 : multicellular organismal process; 0006811 : ion transport; 0006836 : neurotransmitter transport; 0051641 : cellular localization; 0007154 : cell communication; 0046903 : secretion; 0065008 : regulation of biological quality		SLC32A1	616440	316.4
http://www.uniprot.org/uniprot/Q96B33	Claudin-23	0005515 : protein binding; 0005198 : structural molecule activity	0005575 : cellular_component; 0016020 : membrane	0007155 : cell adhesion	Cell adhesion molecules (CAMs) [PATH:hsa04514]137075. Tight junction [PATH:hsa04530]137075. Leukocyte transendothelial migration [PATH:hsa04670]137075. Hepatitis C [PATH:hsa05160]137075	CLDN23	609203	315.2
http://www.uniprot.org/uniprot/Q6UXZ3	CMRF35-like molecule 4 precursor		0016020 : membrane	0002376 : immune system process; 0050789 : regulation of biological process; 0050896 : response to stimulus		CD300LD	616301	312.9

http://www.uniprot.org/uniprot/Q96A08	Histone H2B type 1-A	0003676 : nucleic acid binding; 0005515 : protein binding	0016020 : membrane; 0005634 : nucleus; 0005694 : chromosome; 0000786 : nucleosome	0050896 : response to stimulus; 0002376 : immune system process; 0006928 : movement of cell or subcellular component; 0040011 : locomotion; 0051179 : localization; 0006323 : DNA packaging; 0065003 : protein-containing complex assembly; 0022411 : cellular component disassembly; 0051276 : chromosome organization; 0006508 : proteolysis; 0051604 : protein maturation; 0065009 : regulation of molecular function; 0006464 : cellular protein modification process; 0000003 : reproduction; 0006997 : nucleus organization; 0032501 : multicellular organismal process; 0032502 : developmental process; 0051704 : multi-organism process	Systemic lupus erythematosus [PATH:hsa05322]255626	HIST1H2BA	609904	307
http://www.uniprot.org/uniprot/Q9HB89	Neuromedin-U receptor 1	0038023 : signaling receptor activity; 0042277 : peptide binding	0016020 : membrane; 0044464 : cell part	0065009 : regulation of molecular function; 0006811 : ion transport; 0007154 : cell communication; 0050789 : regulation of biological process; 0050896 : response to stimulus; 0007005 : mitochondrion organization; 0015031 : protein transport; 0051641 : cellular localization; 0006936 : muscle contraction	Neuroactive ligand-receptor interaction [PATH:hsa04080]10316	NMUR1	604153	305.8
http://www.uniprot.org/uniprot/Q6PEY2	Tubulin alpha-3E chain	0000166 : nucleotide binding; 0001882 : nucleoside binding; 0043167 : ion binding; 0016787 : hydrolase activity; 0005198 : structural molecule activity	0005737 : cytoplasm; 0005856 : cytoskeleton; 0005634 : nucleus	0008150 : biological_process	Phagosome [PATH:hsa04145]112714. Gap junction [PATH:hsa04540]112714. Pathogenic Escherichia coli infection [PATH:hsa05130]112714	TUBA3E		305.2
http://www.uniprot.org/uniprot/Q8NGC4	Olfactory receptor 10G3	0038023 : signaling receptor activity	0016020 : membrane	0007154 : cell communication; 0050789 : regulation of biological process; 0050896 : response to stimulus; 0050877 : nervous system process	Olfactory transduction [PATH:hsa04740]26533	OR10G3		304.5
http://www.uniprot.org/uniprot/Q9C0J9	Class E basic helix-loop-helix protein 41	0005515 : protein binding; 0003676 : nucleic acid binding; 0003674 : molecular_function	0005634 : nucleus	0032501 : multicellular organismal process; 0032502 : developmental process; 0008283 : cell proliferation; 0043170 : macromolecule metabolic process; 0048511 : rhythmic process; 0050789 : regulation of biological process; 0006351 : transcription, DNA-templated	Circadian rhythm - mammal [PATH:hsa04710]79365	BHLHE41	606200; 612975	296
http://www.uniprot.org/uniprot/Q9H221	ATP-binding cassette sub-family G member 8	0000166 : nucleotide binding; 0008144 : drug binding; 0043167 : ion binding; 0005215 : transporter activity; 0016787 : hydrolase activity; 0005515 : protein binding	0016020 : membrane; 0045177 : apical part of cell; 0043190 : ATP-binding cassette (ABC) transporter complex; 0043235 : receptor complex	0006869 : lipid transport; 0065008 : regulation of biological quality; 0007588 : excretion; 0007586 : digestion; 0050789 : regulation of biological process; 0006810 : transport	ABC transporters [PATH:hsa02010]64241. Fat digestion and absorption [PATH:hsa04975]64241. Bile secretion [PATH:hsa04976]64241	ABCG8	210250; 605460; 611465	291.6
http://www.uniprot.org/uniprot/Q9UHD9	Ubiquilin-2		0005773 : vacuole; 0005737 : cytoplasm; 0031982 : vesicle; 0043229 : intracellular organelle; 0005634 : nucleus; 0016020 : membrane	0006914 : autophagy; 0016043 : cellular component organization; 0016192 : vesicle-mediated transport; 0050789 : regulation of biological process; 0007154 : cell communication; 0043170 : macromolecule metabolic process; 0050896 : response to stimulus; 0006508 : proteolysis; 0009057 : macromolecule catabolic process; 0007033 : vacuole organization; 0022607 : cellular component assembly	Protein processing in endoplasmic reticulum [PATH:hsa04141]29978	UBQLN2	300264; 300857	291
http://www.uniprot.org/uniprot/A6NM03	Olfactory receptor 2AG2	0038023 : signaling receptor activity	0016020 : membrane	0007154 : cell communication; 0050789 : regulation of biological process; 0050896 : response to stimulus; 0050877 : nervous system process	Olfactory transduction [PATH:hsa04740]338755	OR2AG2		284.8
http://www.uniprot.org/uniprot/Q5J5C9	Beta-defensin 121 precursor		0009986 : cell surface; 0005576 : extracellular region	0050896 : response to stimulus; 0051704 : multi-organism process; 0002376 : immune system process		DEFB121	616075	282.9
http://www.uniprot.org/uniprot/Q8NGS2	Olfactory receptor 1J2	0038023 : signaling receptor activity	0016020 : membrane	0007154 : cell communication; 0050789 : regulation of biological process; 0050896 : response to stimulus	Olfactory transduction [PATH:hsa04740]26740	OR1J2		280.3
http://www.uniprot.org/uniprot/Q8NGF6	Olfactory receptor 10W1	0038023 : signaling receptor activity; 0005549 : odorant binding	0016020 : membrane	0007154 : cell communication; 0050789 : regulation of biological process; 0050896 : response to stimulus		OR10W1		279.7
http://www.uniprot.org/uniprot/Q9BQC6	Ribosomal protein 63, mitochondrial	0005198 : structural molecule activity	0005739 : mitochondrion; 0016020 : membrane; 0005840 : ribosome	0006412 : translation; 0022411 : cellular component disassembly		MRPL57	611997	275.6
http://www.uniprot.org/uniprot/Q8NG41	Neuropeptide B precursor	0005515 : protein binding	0005576 : extracellular region	0007154 : cell communication; 0050789 : regulation of biological process; 0050896 : response to stimulus		NPB	607996	274.9
http://www.uniprot.org/uniprot/Q8N687	Beta-defensin 125 precursor		0009986 : cell surface; 0005576 : extracellular region	0050896 : response to stimulus; 0051704 : multi-organism process; 0002376 : immune system process		DEFB125		269

http://www.uniprot.org/uniprot/Q9Y508	E3 ubiquitin-protein ligase RNF114	0043167 : ion binding; 0005515 : protein binding; 0019787 : ubiquitin-like protein transferase activity	0005737 : cytoplasm; 0044464 : cell part; 0005634 : nucleus; 0016020 : membrane	0032502 : developmental process; 0032501 : multicellular organismal process; 0006508 : proteolysis; 0009057 : macromolecule catabolic process; 0050789 : regulation of biological process; 0006464 : cellular protein modification process; 0000003 : reproduction; 0051704 : multi-organism process		RNF114	612451	265.5
http://www.uniprot.org/uniprot/Q30KQ6	Beta-defensin 114 precursor	0008289 : lipid binding	0005576 : extracellular region	0002376 : immune system process; 0050896 : response to stimulus; 0051704 : multi-organism process; 0007154 : cell communication; 0050789 : regulation of biological process; 0006464 : cellular protein modification process; 0006793 : phosphorus metabolic process; 0065009 : regulation of molecular function; 0001816 : cytokine production; 0015031 : protein transport; 0046903 : secretion		DEFB114	615243	265
http://www.uniprot.org/uniprot/Q08ER8	Zinc finger protein 543	0003676 : nucleic acid binding; 0003674 : molecular_function; 0043167 : ion binding	0005634 : nucleus	0006351 : transcription, DNA-templated; 0050789 : regulation of biological process		ZNF543	616847	257.7
http://www.uniprot.org/uniprot/Q96JB6	Lysyl oxidase homolog 4 precursor	0043167 : ion binding; 0016491 : oxidoreductase activity; 0003674 : molecular_function	0031982 : vesicle; 0043230 : extracellular organelle; 0016020 : membrane; 0043235 : receptor complex			LOXL4	607318	252.4
http://www.uniprot.org/uniprot/Q8NGF7	Olfactory receptor 5B17	0038023 : signaling receptor activity; 0005549 : odorant binding	0016020 : membrane	0007154 : cell communication; 0050789 : regulation of biological process; 0050896 : response to stimulus	Olfactory transduction [PATH:hsa04740]219965	OR5B17		250.7
http://www.uniprot.org/uniprot/Q6P9B9	Integrator complex subunit 5		0005737 : cytoplasm; 0016020 : membrane; 0016591 : DNA-directed RNA polymerase II, holoenzyme; 0005634 : nucleus	0006396 : RNA processing; 0006351 : transcription, DNA-templated		INTS5	611349	245.8
http://www.uniprot.org/uniprot/Q96KN9	Gap junction delta-4 protein		0016020 : membrane; 0032991 : protein-containing complex	0007154 : cell communication; 0001775 : cell activation; 0032501 : multicellular organismal process; 0032502 : developmental process; 0040007 : growth; 0050789 : regulation of biological process; 0050896 : response to stimulus		GJD4	611922	241.9
http://www.uniprot.org/uniprot/Q9UKP6	Urotensin-2 receptor	0038023 : signaling receptor activity	0005768 : endosome; 0016020 : membrane	0008015 : blood circulation; 0007154 : cell communication; 0050789 : regulation of biological process; 0050896 : response to stimulus; 0065008 : regulation of biological quality; 0050878 : regulation of body fluid levels; 0007588 : excretion; 0046903 : secretion; 0032501 : multicellular organismal process; 0032502 : developmental process; 0016043 : cellular component organization; 0016049 : cell growth; 0030431 : sleep; 0048511 : rhythmic process; 0008283 : cell proliferation	Neuroactive ligand-receptor interaction [PATH:hsa04080]2837	UTS2R	600896	235.3
http://www.uniprot.org/uniprot/Q9HCE9	Anoctamin-8	0005215 : transporter activity	0005783 : endoplasmic reticulum; 0016020 : membrane	0019538 : protein metabolic process; 0006811 : ion transport; 0006464 : cellular protein modification process		ANO8	610216	233.5
http://www.uniprot.org/uniprot/Q9BVK6	Transmembrane emp24 domain-containing protein 9 precursor	0005515 : protein binding	0005783 : endoplasmic reticulum; 0016020 : membrane; 0005737 : cytoplasm; 0043231 : intracellular membrane-bounded organelle; 0031982 : vesicle; 0043230 : extracellular organelle; 0005794 : Golgi apparatus; 0043229 : intracellular organelle	0016050 : vesicle organization; 0016192 : vesicle-mediated transport; 0051640 : organelle localization; 0061024 : membrane organization; 0051641 : cellular localization; 0007030 : Golgi organization; 0006996 : organelle organization; 0050789 : regulation of biological process; 0015031 : protein transport		TMED9		223.2
http://www.uniprot.org/uniprot/Q8NGI6	Olfactory receptor 4D10	0038023 : signaling receptor activity	0016020 : membrane	0050877 : nervous system process; 0051606 : detection of stimulus; 0007154 : cell communication; 0050789 : regulation of biological process; 0050896 : response to stimulus	Olfactory transduction [PATH:hsa04740]390197	OR4D10		222.6
http://www.uniprot.org/uniprot/Q6UXV4	MICOS complex subunit MIC27 precursor		0005576 : extracellular region; 0005739 : mitochondrion; 0016020 : membrane; 0032991 : protein-containing complex; 0005737 : cytoplasm; 0031982 : vesicle; 0043229 : intracellular organelle	0007005 : mitochondrion organization; 0061024 : membrane organization; 0016192 : vesicle-mediated transport; 0046903 : secretion		APOOL	300955	221.4
http://www.uniprot.org/uniprot/Q8IYD1	Eukaryotic peptide chain release factor GTP-binding subunit ERF3B	0000166 : nucleotide binding; 0001882 : nucleoside binding; 0043167 : ion binding; 0016787 : hydrolase activity; 0003676 : nucleic acid binding	0005737 : cytoplasm; 0032991 : protein-containing complex	0007049 : cell cycle; 0006412 : translation; 0022411 : cellular component disassembly; 0009057 : macromolecule catabolic process; 0016070 : RNA metabolic process; 0050789 : regulation of biological process	mRNA surveillance pathway [PATH:hsa03015]23708	GSPT2	300418	218.5
http://www.uniprot.org/uniprot/Q9H469	F-box/LRR-repeat protein 15	0019787 : ubiquitin-like protein transferase activity	0005737 : cytoplasm; 0000151 : ubiquitin ligase complex	0032501 : multicellular organismal process; 0032502 : developmental process; 0007049 : cell cycle; 0006351 : transcription, DNA-templated; 0007154 : cell communication; 0050789 : regulation of biological process; 0050896 : response to stimulus; 0006464 : cellular protein modification process; 0006508 : proteolysis; 0009057 : macromolecule catabolic process		FBXL15	610287	210.6
http://www.uniprot.org/uniprot/Q8NGC3	Olfactory receptor 10G2	0038023 : signaling receptor activity	0016020 : membrane	0007154 : cell communication; 0050789 : regulation of biological process; 0050896 : response to stimulus; 0050877 : nervous system process	Olfactory transduction [PATH:hsa04740]26534	OR10G2		208.1

http://www.uniprot.org/uniprot/Q7L7L0	Histone H2A type 3	0003676 : nucleic acid binding; 0005515 : protein binding	0031982 : vesicle; 0043230 : extracellular organelle; 0000786 : nucleosome; 0005634 : nucleus	0006351 : transcription, DNA-templated; 0016458 : gene silencing; 0051276 : chromosome organization; 0022411 : cellular component disassembly; 0006281 : DNA repair	Systemic lupus erythematosus [PATH:hsa05322]92815	HIST3H2A	615015	206.1
http://www.uniprot.org/uniprot/Q9UBT3	Dickkopf-related protein 4 precursor		0005576 : extracellular region	0032501 : multicellular organismal process; 0032502 : developmental process; 0007154 : cell communication; 0050789 : regulation of biological process; 0050896 : response to stimulus; 0042303 : molting cycle	Wnt signaling pathway [PATH:hsa04310]27121	DKK4	605417	203.4
http://www.uniprot.org/uniprot/Q9NX09	DNA damage-inducible transcript 4 protein	0005515 : protein binding	0005737 : cytoplasm; 0044464 : cell part; 0005739 : mitochondrion	0032501 : multicellular organismal process; 0032502 : developmental process; 0008283 : cell proliferation; 0050896 : response to stimulus; 0002376 : immune system process; 0051704 : multi-organism process; 0007154 : cell communication; 0050789 : regulation of biological process; 0005975 : carbohydrate metabolic process; 0006082 : organic acid metabolic process; 0006091 : generation of precursor metabolites and energy; 0009117 : nucleotide metabolic process; 0017144 : drug metabolic process; 0051186 : cofactor metabolic process; 0006464 : cellular protein modification process; 0006793 : phosphorus metabolic process; 0006928 : movement of cell or subcellular component; 0040011 : locomotion; 0051179 : localization; 0008152 : metabolic process	mTOR signaling pathway [PATH:hsa04150]54541	DDIT4	607729	202.7
http://www.uniprot.org/uniprot/Q8NGT7	Olfactory receptor 2A12	0038023 : signaling receptor activity	0016020 : membrane	0007154 : cell communication; 0050789 : regulation of biological process; 0050896 : response to stimulus; 0050877 : nervous system process	Olfactory transduction [PATH:hsa04740]346525	OR2A12		200.1
http://www.uniprot.org/uniprot/Q9H7S9	Zinc finger protein 703	0043167 : ion binding; 0003676 : nucleic acid binding; 0005515 : protein binding	0005737 : cytoplasm; 0005634 : nucleus; 0032991 : protein-containing complex	0022607 : cellular component assembly; 0050896 : response to stimulus; 0032501 : multicellular organismal process; 0032502 : developmental process; 0007155 : cell adhesion; 0050789 : regulation of biological process; 0006351 : transcription, DNA-templated; 0006928 : movement of cell or subcellular component; 0040011 : locomotion; 0051179 : localization; 0008283 : cell proliferation; 0007154 : cell communication; 0007049 : cell cycle		ZNF703		197.3
http://www.uniprot.org/uniprot/Q8NGG8	Olfactory receptor 8B3	0038023 : signaling receptor activity; 0005549 : odorant binding	0016020 : membrane	0007154 : cell communication; 0050789 : regulation of biological process; 0050896 : response to stimulus		OR8B3		188.8
http://www.uniprot.org/uniprot/Q8NES8	Beta-defensin 124 precursor		0009986 : cell surface; 0005576 : extracellular region; 0044464 : cell part	0050896 : response to stimulus; 0051704 : multi-organism process; 0007154 : cell communication; 0050789 : regulation of biological process; 0002376 : immune system process; 0001816 : cytokine production; 0015031 : protein transport; 0046903 : secretion; 0006928 : movement of cell or subcellular component; 0040011 : locomotion; 0051179 : localization		DEFB124		187.2
http://www.uniprot.org/uniprot/Q8TBE9	N-acylneuraminate-9-phosphatase	0016787 : hydrolase activity	0005737 : cytoplasm	0005975 : carbohydrate metabolic process; 0008152 : metabolic process; 0006082 : organic acid metabolic process; 0017144 : drug metabolic process	Amino sugar and nucleotide sugar metabolism [PATH:hsa00520]140838. Metabolic pathways [PATH:hsa01100]140838	NANP	610763	180.3
http://www.uniprot.org/uniprot/Q6PGQ7	Protein aurora borealis	0005515 : protein binding	0005737 : cytoplasm	0051301 : cell division; 0007049 : cell cycle; 0000280 : nuclear division; 0050789 : regulation of biological process; 0006996 : organelle organization; 0051179 : localization		BORA	610510	180.2
http://www.uniprot.org/uniprot/Q9NRH3	Tubulin gamma-2 chain	0000166 : nucleotide binding; 0001882 : nucleoside binding; 0043167 : ion binding; 0016787 : hydrolase activity; 0005198 : structural molecule activity	0005737 : cytoplasm; 0005856 : cytoskeleton; 0000930 : gamma-tubulin complex; 0005813 : centrosome	0006996 : organelle organization; 0051258 : protein polymerization		TUBG2	605785	179.5
http://www.uniprot.org/uniprot/Q9Y4C5	Carbohydrate sulfotransferase 2	0016740 : transferase activity	0005794 : Golgi apparatus; 0016020 : membrane	0005975 : carbohydrate metabolic process; 0050896 : response to stimulus; 0006082 : organic acid metabolic process; 0006790 : sulfur compound metabolic process; 0006807 : nitrogen compound metabolic process; 0009059 : macromolecule biosynthetic process; 0032501 : multicellular organismal process; 0032502 : developmental process; 0008152 : metabolic process	Glycosaminoglycan biosynthesis - keratan sulfate [PATH:hsa00533]9435	CHST2	603798	175.8
http://www.uniprot.org/uniprot/Q7Z7E8	Ubiquitin-conjugating enzyme E2 Q1	0000166 : nucleotide binding; 0008144 : drug binding; 0043167 : ion binding; 0019787 : ubiquitin-like protein transferase activity	0005737 : cytoplasm; 0030175 : filopodium; 0005634 : nucleus	0000003 : reproduction; 0032501 : multicellular organismal process; 0032502 : developmental process; 0051704 : multi-organism process; 0007154 : cell communication; 0009914 : hormone transport; 0015031 : protein transport; 0046903 : secretion; 0051705 : multi-organism behavior	Ubiquitin mediated proteolysis [PATH:hsa04120]55585	UBE2Q1	617429	174.2
http://www.uniprot.org/uniprot/Q8NGU9	Probable G-protein coupled receptor 150	0038023 : signaling receptor activity	0016020 : membrane	0007154 : cell communication; 0050789 : regulation of biological process; 0050896 : response to stimulus		GPR150		172.8

http://www.uniprot.org/uniprot/Q9Y6F9	Protein Wnt-6 precursor	0005515 : protein binding	0009986 : cell surface; 0005737 : cytoplasm; 0016020 : membrane; 0031982 : vesicle; 0043229 : intracellular organelle; 0005783 : endoplasmic reticulum; 0043230 : extracellular organelle; 0005576 : extracellular region; 0005794 : Golgi apparatus; 0031012 : extracellular matrix	0032501 : multicellular organismal process; 0032502 : developmental process; 0050896 : response to stimulus; 0007154 : cell communication; 0043170 : macromolecule metabolic process; 0050789 : regulation of biological process; 0006351 : transcription, DNA-templated	Wnt signaling pathway [PATH:hsa04310]7475. Hedgehog signaling pathway [PATH:hsa04340]7475. Melanogenesis [PATH:hsa04916]7475. Pathways in cancer [PATH:hsa05200]7475. Basal cell carcinoma [PATH:hsa05217]7475	WNT6	604663	172.7
http://www.uniprot.org/uniprot/Q8NGR5	Olfactory receptor 1L4	0038023 : signaling receptor activity	0016020 : membrane	0007154 : cell communication; 0050789 : regulation of biological process; 0050896 : response to stimulus	Olfactory transduction [PATH:hsa04740]254973	OR1L4		169.6
http://www.uniprot.org/uniprot/Q8NBJ7	Sulfatase-modifying factor 2 precursor	0043167 : ion binding	0005783 : endoplasmic reticulum	0006464 : cellular protein modification process		SUMF2 ORFNames	607940	169.6
http://www.uniprot.org/uniprot/Q6V0L0	Cytochrome P450 26C1	0046906 : tetrapyrrole binding; 0048037 : cofactor binding; 0043167 : ion binding; 0004497 : monooxygenase activity; 0019840 : isoprenoid binding; 0031406 : carboxylic acid binding	0005783 : endoplasmic reticulum; 0016020 : membrane	0032501 : multicellular organismal process; 0032502 : developmental process; 0007154 : cell communication; 0050789 : regulation of biological process; 0050896 : response to stimulus; 0048284 : organelle fusion; 0055114 : oxidation-reduction process; 0006082 : organic acid metabolic process; 0006766 : vitamin metabolic process; 0042445 : hormone metabolic process; 0044255 : cellular lipid metabolic process; 0006629 : lipid metabolic process	Retinol metabolism [PATH:hsa00830]340665	CYP26C1	608428; 614974	167.2
http://www.uniprot.org/uniprot/Q6IFH4	Olfactory receptor 6B2	0038023 : signaling receptor activity; 0005549 : odorant binding	0016020 : membrane	0007154 : cell communication; 0050789 : regulation of biological process; 0050896 : response to stimulus	Olfactory transduction [PATH:hsa04740]389090	OR6B2		167.1
http://www.uniprot.org/uniprot/Q96A46	Mitoferrin-2	0005215 : transporter activity	0016020 : membrane; 0005739 : mitochondrion	0006811 : ion transport; 0065008 : regulation of biological quality; 0006810 : transport		SLC25A28	609767	166.3
http://www.uniprot.org/uniprot/Q9Y4Z2	Neurogenin-3	0003676 : nucleic acid binding; 0003682 : chromatin binding; 0005515 : protein binding; 0003674 : molecular_function	0005737 : cytoplasm; 0005634 : nucleus	0032501 : multicellular organismal process; 0032502 : developmental process; 0006351 : transcription, DNA-templated; 0050789 : regulation of biological process; 0065009 : regulation of molecular function; 0032989 : cellular component morphogenesis	Maturity onset diabetes of the young [PATH:hsa04950]50674	NEUROG3	604882; 610370	166.2
http://www.uniprot.org/uniprot/Q6PIU2	Neutral cholesterol ester hydrolase 1	0016787 : hydrolase activity	0005783 : endoplasmic reticulum; 0016020 : membrane	0008152 : metabolic process; 0006629 : lipid metabolic process; 0032501 : multicellular organismal process; 0050789 : regulation of biological process; 0006805 : xenobiotic metabolic process	Bile secretion [PATH:hsa04976]57552	NCEH1	613234	165
http://www.uniprot.org/uniprot/Q96RP8	Potassium voltage-gated channel subfamily A member 7	0005215 : transporter activity	0016020 : membrane; 0032991 : protein-containing complex	0006811 : ion transport; 0065003 : protein-containing complex assembly; 0050789 : regulation of biological process		KCNA7	176268	160.1
http://www.uniprot.org/uniprot/Q96RA2	Olfactory receptor 7D2	0038023 : signaling receptor activity	0016020 : membrane	0050877 : nervous system process; 0051606 : detection of stimulus; 0007154 : cell communication; 0050789 : regulation of biological process; 0050896 : response to stimulus; 0006351 : transcription, DNA-templated	Olfactory transduction [PATH:hsa04740]162998	OR7D2		158.4
http://www.uniprot.org/uniprot/Q9NZK7	Group IIE secretory phospholipase A2 precursor	0043167 : ion binding; 0016787 : hydrolase activity	0005576 : extracellular region	0006811 : ion transport; 0006869 : lipid transport; 0015849 : organic acid transport; 0046903 : secretion; 0050896 : response to stimulus; 0006629 : lipid metabolic process; 0032501 : multicellular organismal process; 0043062 : extracellular structure organization; 0050789 : regulation of biological process; 0006793 : phosphorus metabolic process; 0044255 : cellular lipid metabolic process; 0006807 : nitrogen compound metabolic process	Glycerophospholipid metabolism [PATH:hsa00564]30814. Ether lipid metabolism [PATH:hsa00565]30814. Arachidonic acid metabolism [PATH:hsa00590]30814. Linoleic acid metabolism [PATH:hsa00591]30814. alpha-Linolenic acid metabolism [PATH:hsa00592]30814. Metabolic pathways [PATH:hsa01100]30814. MAPK signaling pathway [PATH:hsa04010]30814. Vascular smooth muscle contraction [PATH:hsa04270]30814. VEGF signaling pathway [PATH:hsa04370]30814. Fc epsilon RI signaling pathway [PATH:hsa04664]30814. Glutamatergic synapse [PATH:hsa04724]30814. Long-term depression [PATH:hsa04730]30814.	PLA2G2E		157.6

					GnRH signaling pathway [PATH:hsa04912]30814. Pancreatic secretion [PATH:hsa04972]30814. Fat digestion and absorption [PATH:hsa04975]30814. Toxoplasmosis [PATH:hsa05145]30814			
http://www.uniprot.org/uniprot/Q9H461	Frizzled-8 precursor	0038023 : signaling receptor activity; 0005515 : protein binding	0005794 : Golgi apparatus; 0016020 : membrane; 0032991 : protein-containing complex	0032501 : multicellular organismal process; 0032502 : developmental process; 0007154 : cell communication; 0050789 : regulation of biological process; 0050896 : response to stimulus; 0006351 : transcription, DNA-templated; 0006464 : cellular protein modification process; 0006793 : phosphorus metabolic process; 0065009 : regulation of molecular function; 0001775 : cell activation; 0002376 : immune system process	Wnt signaling pathway [PATH:hsa04310]8325. Melanogenesis [PATH:hsa04916]8325. Pathways in cancer [PATH:hsa05200]8325. Basal cell carcinoma [PATH:hsa05217]8325	FZD8	606146	157.5
http://www.uniprot.org/uniprot/Q7Z7A3	Cytoplasmic tRNA 2-thiolation protein 1	0016740 : transferase activity; 0003676 : nucleic acid binding	0005737 : cytoplasm; 0005739 : mitochondrion	0006396 : RNA processing; 0043412 : macromolecule modification	Sulfur relay system [PATH:hsa04122]90353	CTU1	612694	156.7
http://www.uniprot.org/uniprot/Q8NGV0	Olfactory receptor 2Y1	0038023 : signaling receptor activity	0016020 : membrane	0007154 : cell communication; 0050789 : regulation of biological process; 0050896 : response to stimulus; 0050877 : nervous system process	Olfactory transduction [PATH:hsa04740]134083	OR2Y1		151.2
http://www.uniprot.org/uniprot/Q9Y6H1	Coiled-coil-helix-coiled-coil-helix domain-containing protein 2	0003676 : nucleic acid binding; 0005515 : protein binding	0005739 : mitochondrion; 0005634 : nucleus	0007005 : mitochondrion organization; 0006351 : transcription, DNA-templated; 0050789 : regulation of biological process; 0050896 : response to stimulus		CHCHD2	616244/	150.3
http://www.uniprot.org/uniprot/Q6ZYL4	General transcription factor IIH subunit 5	0003676 : nucleic acid binding	0000439 : core TFIIH complex; 0005730 : nucleolus; 0005634 : nucleus; 0005667 : transcription factor complex; 0016591 : DNA-directed RNA polymerase II, holoenzyme	0006396 : RNA processing; 0050896 : response to stimulus; 0006281 : DNA repair; 0032392 : DNA geometric change; 0065003 : protein-containing complex assembly; 0050789 : regulation of biological process; 0006351 : transcription, DNA-templated; 0042254 : ribosome biogenesis; 0006354 : DNA-templated transcription, elongation	Basal transcription factors [PATH:hsa03022]404672. Nucleotide excision repair [PATH:hsa03420]404672	GTF2H5	608780; 616395	150.2
http://www.uniprot.org/uniprot/Q8TAE8	Growth arrest and DNA damage-inducible proteins-interacting protein 1		0005739 : mitochondrion; 0005840 : ribosome; 0005634 : nucleus	0006412 : translation; 0022411 : cellular component disassembly; 0007049 : cell cycle; 0050789 : regulation of biological process; 0016032 : viral process		GADD45GIP1	605162	149.8
http://www.uniprot.org/uniprot/Q9UGM1	Neuronal acetylcholine receptor subunit alpha-9 precursor	0005215 : transporter activity; 0038023 : signaling receptor activity	0016020 : membrane; 0043235 : receptor complex; 0005575 : cellular_component	0050877 : nervous system process; 0051606 : detection of stimulus; 0032501 : multicellular organismal process; 0032502 : developmental process; 0065008 : regulation of biological quality	Neuroactive ligand-receptor interaction [PATH:hsa04080]55584	CHRNA9	605116	146.8

Genes Hypermethylated in Spastic Cerebral Palsy Subjects

		Gene Ontologies						
UniProtID_Link	Protein Name	Function	Component	Process	KEGG Pathway	HUGO Gene Name	OMIM ID	Methylation Score
http://www.uniprot.org/uniprot/Q8NH50	Olfactory receptor 8K5	0038023 : signaling receptor activity; 0005549 : odorant binding	0016020 : membrane	0007154 : cell communication; 0050789 : regulation of biological process; 0050896 : response to stimulus	Olfactory transduction [PATH:hsa04740]219453	OR8K5		-2069.8
http://www.uniprot.org/uniprot/Q8NGC2	Olfactory receptor 4E2	0038023 : signaling receptor activity	0016020 : membrane	0050877 : nervous system process; 0051606 : detection of stimulus; 0007154 : cell communication; 0050789 : regulation of biological process; 0050896 : response to stimulus	Olfactory transduction [PATH:hsa04740]26686	OR4E2		-1580.3
http://www.uniprot.org/uniprot/A6NL26	Olfactory receptor 5B21	0038023 : signaling receptor activity; 0005549 : odorant binding	0016020 : membrane	0007154 : cell communication; 0050789 : regulation of biological process; 0050896 : response to stimulus	Olfactory transduction [PATH:hsa04740]219968	OR5B21		-1571.4
http://www.uniprot.org/uniprot/Q8N146	Olfactory receptor 8H3	0038023 : signaling receptor activity; 0005549 : odorant binding	0016020 : membrane	0007154 : cell communication; 0050789 : regulation of biological process; 0050896 : response to stimulus	Olfactory transduction [PATH:hsa04740]390152	OR8H3		-1562.7
http://www.uniprot.org/uniprot/Q9H2C8	Olfactory receptor 51V1	0038023 : signaling receptor activity	0016020 : membrane	0050877 : nervous system process; 0007154 : cell communication; 0050789 : regulation of biological process; 0050896 : response to stimulus	Olfactory transduction [PATH:hsa04740]283111	OR51V1		-1516.8
http://www.uniprot.org/uniprot/Q30KQ7	Beta-defensin 113 precursor		0005576 : extracellular region	0050896 : response to stimulus; 0051704 : multi-organism process; 0002376 : immune system process		DEFB113		-1176.6
http://www.uniprot.org/uniprot/Q8NGS6	Olfactory receptor 13C3	0038023 : signaling receptor activity	0016020 : membrane	0007154 : cell communication; 0050789 : regulation of biological process; 0050896 : response to stimulus; 0050877 : nervous system process	Olfactory transduction [PATH:hsa04740]138803	OR13C3		-1113.9
http://www.uniprot.org/uniprot/Q8NGI4	Olfactory receptor 4D11	0038023 : signaling receptor activity	0016020 : membrane	0050877 : nervous system process; 0051606 : detection of stimulus; 0007154 : cell communication; 0050789 : regulation of biological process; 0050896 : response to stimulus	Olfactory transduction [PATH:hsa04740]219986	OR4D11		-1021.2
http://www.uniprot.org/uniprot/Q8N4P2	Tetratricopeptide repeat protein 30B		0005929 : cilium; 0030990 : intraciliary transport particle	0006996 : organelle organization; 0022607 : cellular component assembly; 0006810 : transport; 0006928 : movement of cell or subcellular component; 0051641 : cellular localization		TTC30B		-1018.8
http://www.uniprot.org/uniprot/Q8NH18	Olfactory receptor 5J2	0038023 : signaling receptor activity; 0005549 : odorant binding	0016020 : membrane	0007154 : cell communication; 0050789 : regulation of biological process; 0050896 : response to stimulus	Olfactory transduction [PATH:hsa04740]282775	OR5J2		-987.9
http://www.uniprot.org/uniprot/Q8NH10	Olfactory receptor 8U1	0038023 : signaling receptor activity; 0005549 : odorant binding	0016020 : membrane	0007154 : cell communication; 0050789 : regulation of biological process; 0050896 : response to stimulus	Olfactory transduction [PATH:hsa04740]219417	OR8U1		-968.2
http://www.uniprot.org/uniprot/P0C0P6	Neuropeptide S precursor		0005576 : extracellular region	0007154 : cell communication; 0050789 : regulation of biological process; 0050896 : response to stimulus; 0065008 : regulation of biological quality; 0032501 : multicellular organismal process; 0048511 : rhythmic process; 0050877 : nervous system process		NPS	609513	-939.3
http://www.uniprot.org/uniprot/Q8NGM8	Olfactory receptor 6M1	0038023 : signaling receptor activity	0016020 : membrane		Olfactory transduction [PATH:hsa04740]390261	OR6M1		-920.3
http://www.uniprot.org/uniprot/Q8NGS3	Olfactory receptor 1J1	0038023 : signaling receptor activity	0016020 : membrane	0007154 : cell communication; 0050789 : regulation of biological process; 0050896 : response to stimulus	Olfactory transduction [PATH:hsa04740]347168	OR1J1		-913.4
http://www.uniprot.org/uniprot/Q8NGD4	Olfactory receptor 4K1	0038023 : signaling receptor activity	0016020 : membrane	0050877 : nervous system process; 0051606 : detection of stimulus; 0007154 : cell communication; 0050789 : regulation of biological process; 0050896 : response to stimulus	Olfactory transduction [PATH:hsa04740]79544	OR4K1		-870
http://www.uniprot.org/uniprot/Q8WZ84	Olfactory receptor 8D1	0038023 : signaling receptor activity; 0005549 : odorant binding	0016020 : membrane	0007154 : cell communication; 0050789 : regulation of biological process; 0050896 : response to stimulus	Olfactory transduction [PATH:hsa04740]283159	OR8D1		-867.2
http://www.uniprot.org/uniprot/C9JJH3	Ubiquitin carboxyl-terminal hydrolase 17-like protein 10	0008233 : peptidase activity	0005783 : endoplasmic reticulum; 0005634 : nucleus	0006464 : cellular protein modification process; 0006508 : proteolysis; 0009057 : macromolecule catabolic process		USP17L10		-790.8
http://www.uniprot.org/uniprot/Q8NGK3	Olfactory receptor 52K2	0038023 : signaling receptor activity	0016020 : membrane	0007154 : cell communication; 0050789 : regulation of biological process; 0050896 : response to stimulus	Olfactory transduction [PATH:hsa04740]119774	OR52K2		-758
http://www.uniprot.org/uniprot/Q8NGD1	Olfactory receptor 4N2	0038023 : signaling receptor activity	0016020 : membrane	0050877 : nervous system process; 0051606 : detection of stimulus; 0007154 : cell communication; 0050789 : regulation of biological process; 0050896 : response to stimulus	Olfactory transduction [PATH:hsa04740]390429	OR4N2		-691.7
http://www.uniprot.org/uniprot/Q8NGH7	Olfactory receptor 52L1	0038023 : signaling receptor activity	0016020 : membrane	0007154 : cell communication; 0050789 : regulation of biological process; 0050896 : response to stimulus	Olfactory transduction [PATH:hsa04740]338751	OR52L1		-691.4

http://www.uniprot.org/uniprot/Q969Q0	60S ribosomal protein L36a-like	0005198 : structural molecule activity	0005737 : cytoplasm; 0005840 : ribosome; 0005783 : endoplasmic reticulum; 0005634 : nucleus; 0016020 : membrane	0006412 : translation	Ribosome [PATH:hsa03010]6166	RPL36AL	180469	-671
http://www.uniprot.org/uniprot/Q8NGL6	Olfactory receptor 4A15	0038023 : signaling receptor activity	0016020 : membrane	0050877 : nervous system process; 0051606 : detection of stimulus; 0007154 : cell communication; 0050789 : regulation of biological process; 0050896 : response to stimulus	Olfactory transduction [PATH:hsa04740]81328	OR4A15		-664.1
http://www.uniprot.org/uniprot/Q5JQS5	Olfactory receptor 2B11	0038023 : signaling receptor activity	0016020 : membrane	0007154 : cell communication; 0050789 : regulation of biological process; 0050896 : response to stimulus; 0050877 : nervous system process	Olfactory transduction [PATH:hsa04740]127623	OR2B11		-650.6
http://www.uniprot.org/uniprot/Q30KQ1	Beta-defensin 133 precursor		0009986 : cell surface; 0005576 : extracellular region	0050896 : response to stimulus; 0051704 : multi-organism process; 0002376 : immune system process		DEFB133		-645.6
http://www.uniprot.org/uniprot/Q8NGE3	Olfactory receptor 10P1	0038023 : signaling receptor activity	0016020 : membrane	0007154 : cell communication; 0050789 : regulation of biological process; 0050896 : response to stimulus; 0050877 : nervous system process	Olfactory transduction [PATH:hsa04740]121130	OR10P1		-631
http://www.uniprot.org/uniprot/Q8NGT1	Olfactory receptor 2K2	0038023 : signaling receptor activity	0016020 : membrane	0007154 : cell communication; 0050789 : regulation of biological process; 0050896 : response to stimulus; 0050877 : nervous system process	Olfactory transduction [PATH:hsa04740]26248	OR2K2		-629.3
http://www.uniprot.org/uniprot/Q96RI8	Trace amine-associated receptor 6	0038023 : signaling receptor activity	0016020 : membrane	0007154 : cell communication; 0050789 : regulation of biological process; 0050896 : response to stimulus	Neuroactive ligand-receptor interaction [PATH:hsa04080]319100	TAAR6	608923	-618.8
http://www.uniprot.org/uniprot/Q8NGC1	Olfactory receptor 11G2	0038023 : signaling receptor activity	0016020 : membrane		Olfactory transduction [PATH:hsa04740]390439	OR11G2		-599.6
http://www.uniprot.org/uniprot/Q96DR8	Mucin-like protein 1 precursor		0005576 : extracellular region; 0005794 : Golgi apparatus; 0016020 : membrane	0006464 : cellular protein modification process; 0009059 : macromolecule biosynthetic process; 0002376 : immune system process; 0007154 : cell communication; 0050789 : regulation of biological process; 0050896 : response to stimulus		MUCL1	610857	-599.1
http://www.uniprot.org/uniprot/Q6EIG7	C-type lectin domain family 6 member A	0030246 : carbohydrate binding	0016020 : membrane	0002376 : immune system process; 0050896 : response to stimulus; 0051704 : multi-organism process; 0001816 : cytokine production; 0015031 : protein transport; 0046903 : secretion; 0050789 : regulation of biological process; 0007154 : cell communication		CLEC6A	613579	-576.3
http://www.uniprot.org/uniprot/Q8NH94	Olfactory receptor 1L1	0038023 : signaling receptor activity	0016020 : membrane	0007154 : cell communication; 0050789 : regulation of biological process; 0050896 : response to stimulus	Olfactory transduction [PATH:hsa04740]26737	OR1L1		-549.1
http://www.uniprot.org/uniprot/Q8NGF8	Olfactory receptor 4B1	0038023 : signaling receptor activity	0016020 : membrane	0050877 : nervous system process; 0051606 : detection of stimulus; 0007154 : cell communication; 0050789 : regulation of biological process; 0050896 : response to stimulus	Olfactory transduction [PATH:hsa04740]119765	OR4B1		-537.7
http://www.uniprot.org/uniprot/Q9UJU5	Forkhead box protein D3	0003674 : molecular_function; 0003676 : nucleic acid binding	0005634 : nucleus; 0005694 : chromosome	0032502 : developmental process; 0032501 : multicellular organismal process; 0006351 : transcription, DNA-templated; 0050789 : regulation of biological process		FOXD3	607836; 611539	-534.7
http://www.uniprot.org/uniprot/Q8NGL1	Olfactory receptor 5D18	0038023 : signaling receptor activity; 0005549 : odorant binding	0016020 : membrane	0007154 : cell communication; 0050789 : regulation of biological process; 0050896 : response to stimulus	Olfactory transduction [PATH:hsa04740]219438	OR5D18		-509.5
http://www.uniprot.org/uniprot/Q8NGY0	Olfactory receptor 10X1	0038023 : signaling receptor activity	0016020 : membrane		Olfactory transduction [PATH:hsa04740]128367	OR10X1		-498.2
http://www.uniprot.org/uniprot/Q96FJ2	Dynein light chain 2, cytoplasmic	0005515 : protein binding; 0003774 : motor activity	0005813 : centrosome; 0005929 : cilium; 0005856 : cytoskeleton; 0032991 : protein-containing complex; 0005737 : cytoplasm; 0016020 : membrane; 0005739 : mitochondrion; 0005634 : nucleus	0002376 : immune system process; 0006996 : organelle organization; 0022607 : cellular component assembly; 0016192 : vesicle-mediated transport; 0051641 : cellular localization; 0006810 : transport; 0006928 : movement of cell or subcellular component; 0006914 : autophagy; 0065009 : regulation of molecular function; 0007005 : mitochondrion organization; 0007154 : cell communication; 0050789 : regulation of biological process; 0050896 : response to stimulus; 0061024 : membrane organization; 0065008 : regulation of biological quality; 0007049 : cell cycle; 0007059 : chromosome segregation; 0051276 : chromosome organization	Vasopressin-regulated water reabsorption [PATH:hsa04962]140735	DYNLL2	608942	-484.1

http://www.uniprot.org/uniprot/Q8IZX4	Transcription initiation factor TFIID subunit 1-like	0016740 : transferase activity; 0005515 : protein binding; 0003674 : molecular_function; 0003676 : nucleic acid binding	0005634 : nucleus; 0005667 : transcription factor complex; 0016591 : DNA-directed RNA polymerase II, holoenzyme	0000003 : reproduction; 0000280 : nuclear division; 0007049 : cell cycle; 0032501 : multicellular organismal process; 0051704 : multi-organism process; 0006351 : transcription, DNA-templated; 0050789 : regulation of biological process; 0007154 : cell communication; 0050896 : response to stimulus; 0006354 : DNA-templated transcription, elongation	Basal transcription factors [PATH:hsa03022]138474	TAF1L	607798	-475.6
http://www.uniprot.org/uniprot/Q9BZM6	UL16-binding protein 1 precursor	0005515 : protein binding	0005856 : cytoskeleton; 0016020 : membrane; 0005737 : cytoplasm; 0005783 : endoplasmic reticulum	0001775 : cell activation; 0002376 : immune system process; 0001906 : cell killing; 0050896 : response to stimulus; 0050789 : regulation of biological process; 0016032 : viral process	Natural killer cell mediated cytotoxicity [PATH:hsa04650]80329	ULBP1	605697	-458.1
http://www.uniprot.org/uniprot/Q8NDX6	Zinc finger protein 740	0043167 : ion binding; 0003676 : nucleic acid binding	0005634 : nucleus	0006351 : transcription, DNA-templated; 0050789 : regulation of biological process		ZNF740		-442.7
http://www.uniprot.org/uniprot/Q8NG98	Olfactory receptor 7D4	0038023 : signaling receptor activity	0016020 : membrane	0050877 : nervous system process; 0051606 : detection of stimulus; 0007154 : cell communication; 0050789 : regulation of biological process; 0050896 : response to stimulus	Olfactory transduction [PATH:hsa04740]125958	OR7D4	611538	-442.6
http://www.uniprot.org/uniprot/Q30KQ8	Beta-defensin 112 precursor		0009986 : cell surface; 0005576 : extracellular region	0050896 : response to stimulus; 0051704 : multi-organism process; 0002376 : immune system process		DEFB112		-440.6
http://www.uniprot.org/uniprot/Q8NHB1	Olfactory receptor 2V1	0038023 : signaling receptor activity	0016020 : membrane	0007154 : cell communication; 0050789 : regulation of biological process; 0050896 : response to stimulus; 0050877 : nervous system process		OR2V1		-436.4
http://www.uniprot.org/uniprot/Q8NGS1	Olfactory receptor 1J4	0038023 : signaling receptor activity	0016020 : membrane	0007154 : cell communication; 0050789 : regulation of biological process; 0050896 : response to stimulus	Olfactory transduction [PATH:hsa04740]26219	OR1J4		-436
http://www.uniprot.org/uniprot/Q9Y5Y4	Prostaglandin D2 receptor 2	0038023 : signaling receptor activity; 0042277 : peptide binding	0016020 : membrane; 0044464 : cell part; 0043005 : neuron projection	0007154 : cell communication; 0050789 : regulation of biological process; 0050896 : response to stimulus; 0040011 : locomotion; 0002376 : immune system process; 0000003 : reproduction; 0008283 : cell proliferation; 0032501 : multicellular organismal process; 0051704 : multi-organism process		PTGDR2	604837	-431.7
http://www.uniprot.org/uniprot/Q8TB52	F-box only protein 30	0019787 : ubiquitin-like protein transferase activity; 0043167 : ion binding	0005737 : cytoplasm	0006464 : cellular protein modification process		FBXO30	609101	-426.2
http://www.uniprot.org/uniprot/Q96PD4	Interleukin-17F precursor	0005515 : protein binding	0005576 : extracellular region	0032501 : multicellular organismal process; 0032502 : developmental process; 0001816 : cytokine production; 0009059 : macromolecule biosynthetic process; 0019538 : protein metabolic process; 0007154 : cell communication; 0050789 : regulation of biological process; 0050896 : response to stimulus; 0015031 : protein transport; 0046903 : secretion; 0006351 : transcription, DNA-templated		IL17F	606496; 613956	-418.2
http://www.uniprot.org/uniprot/Q8NGI7	Olfactory receptor 10V1	0038023 : signaling receptor activity; 0005549 : odorant binding	0016020 : membrane	0007154 : cell communication; 0050789 : regulation of biological process; 0050896 : response to stimulus	Olfactory transduction [PATH:hsa04740]390201	OR10V1		-416.7
http://www.uniprot.org/uniprot/Q6VVB1	E3 ubiquitin-protein ligase NHLRC1	0043167 : ion binding; 0019787 : ubiquitin-like protein transferase activity	0005737 : cytoplasm; 0005783 : endoplasmic reticulum; 0005634 : nucleus	0006914 : autophagy; 0005976 : polysaccharide metabolic process; 0006112 : energy reserve metabolic process; 0009059 : macromolecule biosynthetic process; 0006464 : cellular protein modification process; 0050789 : regulation of biological process; 0006508 : proteolysis; 0009057 : macromolecule catabolic process	Ubiquitin mediated proteolysis [PATH:hsa04120]378884	NHLRC1	254780; 608072	-413.6
http://www.uniprot.org/uniprot/Q96R48	Olfactory receptor 2A5	0038023 : signaling receptor activity	0016020 : membrane	0007154 : cell communication; 0050789 : regulation of biological process; 0050896 : response to stimulus; 0050877 : nervous system process	Olfactory transduction [PATH:hsa04740]393046	OR2A5		-413.3
http://www.uniprot.org/uniprot/Q8NH01	Olfactory receptor 2T11	0038023 : signaling receptor activity	0016020 : membrane	0007154 : cell communication; 0050789 : regulation of biological process; 0050896 : response to stimulus; 0050877 : nervous system process	Olfactory transduction [PATH:hsa04740]127077	OR2T11		-400.7
http://www.uniprot.org/uniprot/A6NL08	Olfactory receptor 6C75	0038023 : signaling receptor activity	0016020 : membrane		Olfactory transduction [PATH:hsa04740]390323	OR6C75		-398.2
http://www.uniprot.org/uniprot/Q8NGS7	Olfactory receptor 13C8	0038023 : signaling receptor activity	0016020 : membrane	0007154 : cell communication; 0050789 : regulation of biological process; 0050896 : response to stimulus; 0050877 : nervous system process	Olfactory transduction [PATH:hsa04740]138802	OR13C8		-380.6

http://www.uniprot.org/uniprot/Q8NG99	Olfactory receptor 7G2	0038023 : signaling receptor activity	0016020 : membrane	0050877 : nervous system process; 0051606 : detection of stimulus; 0007154 : cell communication; 0050789 : regulation of biological process; 0050896 : response to stimulus	Olfactory transduction [PATH:hsa04740]390882	OR7G2		-370.7
http://www.uniprot.org/uniprot/Q8NGN0	Olfactory receptor 4D5	0038023 : signaling receptor activity	0016020 : membrane	0050877 : nervous system process; 0051606 : detection of stimulus; 0007154 : cell communication; 0050789 : regulation of biological process; 0050896 : response to stimulus	Olfactory transduction [PATH:hsa04740]219875	OR4D5		-364.3
http://www.uniprot.org/uniprot/Q30KP9	Beta-defensin 135 precursor		0009986 : cell surface; 0005576 : extracellular region	0050896 : response to stimulus; 0051704 : multi-organism process; 0002376 : immune system process		DEFB135		-363.2
http://www.uniprot.org/uniprot/Q8IWZ4	Tripartite motif-containing protein 48	0043167 : ion binding	0044464 : cell part			TRIM48		-359.9
http://www.uniprot.org/uniprot/Q9H427	Potassium channel subfamily K member 15	0005215 : transporter activity	0016020 : membrane	0008015 : blood circulation; 0050789 : regulation of biological process; 0006811 : ion transport; 0065008 : regulation of biological quality		KCNK15	607368	-359.1
http://www.uniprot.org/uniprot/Q9NYK1	Toll-like receptor 7 precursor	0003676 : nucleic acid binding; 0008144 : drug binding; 0038023 : signaling receptor activity	0005737 : cytoplasm; 0031982 : vesicle; 0043229 : intracellular organelle; 0005768 : endosome; 0005773 : vacuole; 0016020 : membrane; 0005783 : endoplasmic reticulum; 0005794 : Golgi apparatus; 0043235 : receptor complex	0050896 : response to stimulus; 0002376 : immune system process; 0051704 : multi-organism process; 0006464 : cellular protein modification process; 0006793 : phosphorus metabolic process; 0007154 : cell communication; 0050789 : regulation of biological process; 0001775 : cell activation; 0001816 : cytokine production; 0009059 : macromolecule biosynthetic process; 0019538 : protein metabolic process	Toll-like receptor signaling pathway [PATH:hsa04620]51284. Measles [PATH:hsa05162]51284. Influenza A [PATH:hsa05164]51284	TLR7 ORFNames	300365	-359
http://www.uniprot.org/uniprot/Q8NGQ4	Olfactory receptor 10Q1	0038023 : signaling receptor activity; 0005549 : odorant binding	0016020 : membrane	0007154 : cell communication; 0050789 : regulation of biological process; 0050896 : response to stimulus	Olfactory transduction [PATH:hsa04740]219960	OR10Q1		-350.7
http://www.uniprot.org/uniprot/Q6VVX0	Vitamin D 25-hydroxylase	0046906 : tetrapyrrole binding; 0048037 : cofactor binding; 0043167 : ion binding; 0016491 : oxidoreductase activity; 0004497 : monooxygenase activity	0005783 : endoplasmic reticulum; 0016020 : membrane; 0043226 : organelle	0050896 : response to stimulus; 0006629 : lipid metabolic process; 0006766 : vitamin metabolic process	Steroid biosynthesis [PATH:hsa00100]120227. Metabolic pathways [PATH:hsa01100]120227	CYP2R1	600081; 608713	-340.7
http://www.uniprot.org/uniprot/Q8NGA5	Olfactory receptor 10H4	0038023 : signaling receptor activity	0016020 : membrane		Olfactory transduction [PATH:hsa04740]126541	OR10H4		-339.9
http://www.uniprot.org/uniprot/Q9H0U6	39S ribosomal protein L18, mitochondrial precursor	0003676 : nucleic acid binding; 0005198 : structural molecule activity	0005576 : extracellular region; 0005739 : mitochondrion; 0016020 : membrane; 0005840 : ribosome	0006412 : translation; 0022411 : cellular component disassembly; 0015931 : nucleobase-containing compound transport		MRPL18 ORFNames	611831	-338.3
http://www.uniprot.org/uniprot/Q9NZP0	Olfactory receptor 6C3	0038023 : signaling receptor activity	0016020 : membrane	0050877 : nervous system process	Olfactory transduction [PATH:hsa04740]254786	OR6C3		-335
http://www.uniprot.org/uniprot/Q8NGX0	Olfactory receptor 11L1	0038023 : signaling receptor activity; 0005549 : odorant binding	0031982 : vesicle; 0043230 : extracellular organelle; 0016020 : membrane	0007154 : cell communication; 0050789 : regulation of biological process; 0050896 : response to stimulus	Olfactory transduction [PATH:hsa04740]391189	OR11L1		-333.5
http://www.uniprot.org/uniprot/Q30KP8	Beta-defensin 136 precursor		0005576 : extracellular region	0050896 : response to stimulus; 0051704 : multi-organism process		DEFB136		-331.8
http://www.uniprot.org/uniprot/Q8NGZ9	Olfactory receptor 2T10	0038023 : signaling receptor activity	0016020 : membrane	0007154 : cell communication; 0050789 : regulation of biological process; 0050896 : response to stimulus; 0050877 : nervous system process	Olfactory transduction [PATH:hsa04740]127069	OR2T10		-325.2
http://www.uniprot.org/uniprot/Q96G61	Diphosphoinositol polyphosphate phosphohydrolase 3-beta	0016787 : hydrolase activity; 0043167 : ion binding	0005737 : cytoplasm; 0044464 : cell part; 0005634 : nucleus	0009117 : nucleotide metabolic process; 0006066 : alcohol metabolic process; 0006793 : phosphorus metabolic process		NUDT11	300528	-317.2
http://www.uniprot.org/uniprot/Q96L21	60S ribosomal protein L10-like	0003676 : nucleic acid binding; 0005198 : structural molecule activity	0005737 : cytoplasm; 0005840 : ribosome; 0005783 : endoplasmic reticulum; 0016020 : membrane; 0005634 : nucleus; 0005844 : polysome	0006996 : organelle organization; 0042254 : ribosome biogenesis; 0065003 : protein-containing complex assembly; 0000003 : reproduction; 0032501 : multicellular organismal process; 0051704 : multi-organism process; 0006412 : translation	Ribosome [PATH:hsa03010]140801	RPL10L		-317
http://www.uniprot.org/uniprot/Q8NGE8	Olfactory receptor 4D9	0038023 : signaling receptor activity	0016020 : membrane	0050877 : nervous system process; 0051606 : detection of stimulus; 0007154 : cell communication; 0050789 : regulation of biological process; 0050896 : response to stimulus	Olfactory transduction [PATH:hsa04740]390199	OR4D9		-314.6

http://www.uniprot.org/uniprot/Q6IEG0	U11/U12 small nuclear ribonucleoprotein 48 kDa protein	0043167 : ion binding	0005737 : cytoplasm; 0005634 : nucleus; 0005681 : spliceosomal complex	0000375 : RNA splicing, via transesterification reactions; 0006396 : RNA processing		SNRNP48		-304.8
http://www.uniprot.org/uniprot/Q8WXI8	C-type lectin domain family 4 member D	0030246 : carbohydrate binding; 0005515 : protein binding	0005737 : cytoplasm; 0016020 : membrane; 0031982 : vesicle; 0043229 : intracellular organelle	0002376 : immune system process; 0050896 : response to stimulus; 0051704 : multi-organism process; 0007154 : cell communication; 0050789 : regulation of biological process; 0001775 : cell activation; 0016192 : vesicle-mediated transport; 0046903 : secretion; 0032501 : multicellular organismal process; 0032502 : developmental process		CLEC4D	609964	-303.2
http://www.uniprot.org/uniprot/Q9Y259	Choline/ethanolamine kinase	0000166 : nucleotide binding; 0008144 : drug binding; 0043167 : ion binding; 0016740 : transferase activity	0005737 : cytoplasm	0006793 : phosphorus metabolic process; 0006807 : nitrogen compound metabolic process; 0044255 : cellular lipid metabolic process	Glycerophospholipid metabolism [PATH:hsa00564]1120. Metabolic pathways [PATH:hsa01100]1120	CHKB	612395	-297.6
http://www.uniprot.org/uniprot/A6NP11	Zinc finger protein 716	0003676 : nucleic acid binding; 0043167 : ion binding	0005634 : nucleus	0006351 : transcription, DNA-templated; 0050789 : regulation of biological process		ZNF716		-297.2
http://www.uniprot.org/uniprot/Q8NGA1	Olfactory receptor 1M1	0038023 : signaling receptor activity	0016020 : membrane	0007154 : cell communication; 0050789 : regulation of biological process; 0050896 : response to stimulus	Olfactory transduction [PATH:hsa04740]125963	OR1M1		-294.6
http://www.uniprot.org/uniprot/Q96E22	Dehydrodolichyl diphosphate synthase complex subunit NUS1	0016740 : transferase activity	0005783 : endoplasmic reticulum; 0016020 : membrane	0032501 : multicellular organismal process; 0032502 : developmental process; 0065008 : regulation of biological quality; 0006066 : alcohol metabolic process; 0044255 : cellular lipid metabolic process; 0006464 : cellular protein modification process; 0006793 : phosphorus metabolic process; 0009059 : macromolecule biosynthetic process; 0006928 : movement of cell or subcellular component; 0040011 : locomotion; 0050789 : regulation of biological process; 0051179 : localization; 0008152 : metabolic process; 0065009 : regulation of molecular function; 0006869 : lipid transport; 0051641 : cellular localization; 0007154 : cell communication; 0050896 : response to stimulus		NUS1	610463; 617082	-292.7
http://www.uniprot.org/uniprot/Q9H3M9	Ataxin-3-like protein	0008233 : peptidase activity	0005737 : cytoplasm; 0005634 : nucleus	0006464 : cellular protein modification process; 0006508 : proteolysis; 0006351 : transcription, DNA-templated; 0050789 : regulation of biological process	Protein processing in endoplasmic reticulum [PATH:hsa04141]92552	ATXN3L	300920	-292
http://www.uniprot.org/uniprot/Q9Y6L6	Solute carrier organic anion transporter family member 1B1	0005215 : transporter activity	0016020 : membrane	0006811 : ion transport; 0006869 : lipid transport; 0015849 : organic acid transport	Bile secretion [PATH:hsa04976]10599	SLCO1B1	237450; 604843	-287.1
http://www.uniprot.org/uniprot/Q9NPZ5	Galactosylgalactosylxylosylprotein 3-beta-glucuronosyltransferase 2	0016740 : transferase activity; 0043167 : ion binding	0005794 : Golgi apparatus; 0016020 : membrane	0005975 : carbohydrate metabolic process; 0006082 : organic acid metabolic process; 0006790 : sulfur compound metabolic process; 0019538 : protein metabolic process; 0009059 : macromolecule biosynthetic process; 0006807 : nitrogen compound metabolic process; 0043170 : macromolecule metabolic process; 0006464 : cellular protein modification process	Glycosaminoglycan biosynthesis - chondroitin sulfate [PATH:hsa00532]135152. Glycosaminoglycan biosynthesis - heparan sulfate [PATH:hsa00534]135152. Metabolic pathways [PATH:hsa01100]135152	B3GAT2	607497	-286.1
http://www.uniprot.org/uniprot/Q9UQC9	Calcium-activated chloride channel regulator 2 precursor	0005215 : transporter activity; 0043167 : ion binding; 0008233 : peptidase activity	0016020 : membrane; 0045178 : basal part of cell; 0005575 : cellular_component; 0005737 : cytoplasm; 0005576 : extracellular region; 0005634 : nucleus	0007155 : cell adhesion; 0006811 : ion transport	Olfactory transduction [PATH:hsa04740]9635. Pancreatic secretion [PATH:hsa04972]9635	CLCA2	604003	-285.8
http://www.uniprot.org/uniprot/Q9BYN8	28S ribosomal protein S26, mitochondrial precursor	0003676 : nucleic acid binding	0005739 : mitochondrion; 0016020 : membrane; 0015935 : small ribosomal subunit; 0005634 : nucleus	0051606 : detection of stimulus; 0006412 : translation; 0022411 : cellular component disassembly		MRPS26	611988	-281.7
http://www.uniprot.org/uniprot/Q7Z7B7	Beta-defensin 132 precursor		0009986 : cell surface; 0005576 : extracellular region; 0044464 : cell part	0050896 : response to stimulus; 0051704 : multi-organism process; 0002376 : immune system process; 0031640 : killing of cells of other organism		DEFB132		-279.8

http://www.uniprot.org/uniprot/Q8NGX5	Olfactory receptor 10K1	0038023 : signaling receptor activity	0016020 : membrane	0050877 : nervous system process; 0051606 : detection of stimulus; 0007154 : cell communication; 0050789 : regulation of biological process; 0050896 : response to stimulus	Olfactory transduction [PATH:hsa04740]391109	OR10K1		-279.1
http://www.uniprot.org/uniprot/Q8NGD3	Olfactory receptor 4K5	0038023 : signaling receptor activity	0016020 : membrane	0050877 : nervous system process; 0051606 : detection of stimulus; 0007154 : cell communication; 0050789 : regulation of biological process; 0050896 : response to stimulus	Olfactory transduction [PATH:hsa04740]79317	OR4K5		-276.2
http://www.uniprot.org/uniprot/Q30KQ5	Beta-defensin 115 precursor		0009986 : cell surface; 0005576 : extracellular region	0050896 : response to stimulus; 0051704 : multi-organism process; 0002376 : immune system process		DEFB115		-275.9
http://www.uniprot.org/uniprot/Q8NGX8	Olfactory receptor 6Y1	0038023 : signaling receptor activity	0016020 : membrane	0007154 : cell communication; 0050789 : regulation of biological process; 0050896 : response to stimulus; 0050877 : nervous system process	Olfactory transduction [PATH:hsa04740]391112	OR6Y1		-275.5
http://www.uniprot.org/uniprot/Q96RD0	Olfactory receptor 8B2	0038023 : signaling receptor activity; 0005549 : odorant binding	0016020 : membrane	0007154 : cell communication; 0050789 : regulation of biological process; 0050896 : response to stimulus		OR8B2		-269.5
http://www.uniprot.org/uniprot/Q8NGX9	Olfactory receptor 6P1	0038023 : signaling receptor activity	0016020 : membrane	0007154 : cell communication; 0050789 : regulation of biological process; 0050896 : response to stimulus; 0050877 : nervous system process		OR6P1		-268.7
http://www.uniprot.org/uniprot/Q8IV77	Cyclic nucleotide-gated cation channel alpha-4	0000166 : nucleotide binding; 0043167 : ion binding; 0005215 : transporter activity	0005929 : cilium; 0016020 : membrane; 0005794 : Golgi apparatus; 0005737 : cytoplasm; 0031982 : vesicle; 0043229 : intracellular organelle	0065008 : regulation of biological quality; 0050896 : response to stimulus; 0050877 : nervous system process	Olfactory transduction [PATH:hsa04740]1262	CNGA4	609472	-260.2
http://www.uniprot.org/uniprot/Q7RTR8	Taste receptor type 2 member 42	0038023 : signaling receptor activity	0016020 : membrane	0007154 : cell communication; 0050789 : regulation of biological process; 0050896 : response to stimulus; 0050877 : nervous system process	Taste transduction [PATH:hsa04742]353164	TAS2R42	613966	-260
http://www.uniprot.org/uniprot/Q8NGN3	Olfactory receptor 10G4	0038023 : signaling receptor activity	0016020 : membrane	0007154 : cell communication; 0050789 : regulation of biological process; 0050896 : response to stimulus; 0050877 : nervous system process	Olfactory transduction [PATH:hsa04740]390264	OR10G4		-259.8
http://www.uniprot.org/uniprot/Q9UKL4	Gap junction delta-2 protein	0005215 : transporter activity	0016020 : membrane; 0032991 : protein-containing complex	0065008 : regulation of biological quality; 0007154 : cell communication; 0050877 : nervous system process	Gap junction [PATH:hsa04540]57369	GJD2	607058	-258.3
http://www.uniprot.org/uniprot/Q8NGD5	Olfactory receptor 4K14	0038023 : signaling receptor activity	0016020 : membrane	0050877 : nervous system process; 0051606 : detection of stimulus; 0007154 : cell communication; 0050789 : regulation of biological process; 0050896 : response to stimulus	Olfactory transduction [PATH:hsa04740]122740	OR4K14		-257.8
http://www.uniprot.org/uniprot/Q8N4N8	Kinesin-like protein KIF2B	0000166 : nucleotide binding; 0008144 : drug binding; 0043167 : ion binding; 0016787 : hydrolase activity; 0005515 : protein binding; 0003774 : motor activity	0000776 : kinetochore; 0005737 : cytoplasm; 0005576 : extracellular region; 0005856 : cytoskeleton; 0032991 : protein-containing complex; 0005730 : nucleolus	0002376 : immune system process; 0051301 : cell division; 0007049 : cell cycle; 0007059 : chromosome segregation; 0051234 : establishment of localization; 0051640 : organelle localization; 0006996 : organelle organization; 0051261 : protein depolymerization; 0006928 : movement of cell or subcellular component; 0050789 : regulation of biological process; 0016192 : vesicle-mediated transport; 0051276 : chromosome organization		KIF2B	615142	-256.4
http://www.uniprot.org/uniprot/Q2M5E4	Regulator of G-protein signaling 21	0030234 : enzyme regulator activity	0005737 : cytoplasm; 0016020 : membrane	0007154 : cell communication; 0050789 : regulation of biological process; 0050896 : response to stimulus		RGS21	612407	-254.9
http://www.uniprot.org/uniprot/Q9NS69	Mitochondrial import receptor subunit TOM22 homolog	0005215 : transporter activity	0016020 : membrane; 0005739 : mitochondrion; 0032991 : protein-containing complex	0006914 : autophagy; 0050789 : regulation of biological process; 0007005 : mitochondrion organization; 0015031 : protein transport; 0051641 : cellular localization; 0061024 : membrane organization; 0051234 : establishment of localization		TOMM22	607046	-252.9
http://www.uniprot.org/uniprot/Q6IEU7	Olfactory receptor 5M10	0038023 : signaling receptor activity; 0005549 : odorant binding	0016020 : membrane	0007154 : cell communication; 0050789 : regulation of biological process; 0050896 : response to stimulus	Olfactory transduction [PATH:hsa04740]390167	OR5M10		-247.1
http://www.uniprot.org/uniprot/Q96PH6	Beta-defensin 118 precursor		0009986 : cell surface; 0005576 : extracellular region	0002376 : immune system process; 0050896 : response to stimulus; 0051704 : multi-organism process; 0007155 : cell adhesion; 0031640 : killing of cells of other organism; 0044419 : interspecies interaction between organisms; 0000003 : reproduction; 0032501 : multicellular organismal process		DEFB118	607650	-245.3
http://www.uniprot.org/uniprot/Q9BSH4	Translational activator of cytochrome c oxidase 1		0005739 : mitochondrion; 0005634 : nucleus	0006412 : translation; 0050789 : regulation of biological process		TACO1	256000; 612958	-244.8

http://www.uniprot.org/uniprot/Q9UJM8	Hydroxyacid oxidase 1	0016491 : oxidoreductase activity; 0000166 : nucleotide binding; 0043167 : ion binding; 0048037 : cofactor binding; 0005515 : protein binding	0042579 : microbody	0006807 : nitrogen compound metabolic process; 0006082 : organic acid metabolic process; 0044255 : cellular lipid metabolic process; 0055114 : oxidation-reduction process; 0006066 : alcohol metabolic process; 0017144 : drug metabolic process; 0050896 : response to stimulus	Glyoxylate and dicarboxylate metabolism [PATH:hsa00630]54363. Metabolic pathways [PATH:hsa01100]54363. Peroxisome [PATH:hsa04146]54363	HAO1	605023	-243.3
http://www.uniprot.org/uniprot/Q8NGW1	Olfactory receptor 6B3	0038023 : signaling receptor activity; 0005549 : odorant binding	0016020 : membrane	0007154 : cell communication; 0050789 : regulation of biological process; 0050896 : response to stimulus	Olfactory transduction [PATH:hsa04740]150681	OR6B3		-239.1
